# Effect of Cavity Disinfectants on Dentin Bond Strength and Clinical Success of Composite Restorations—A Systematic Review of In Vitro, In Situ and Clinical Studies

**DOI:** 10.3390/ijms22010353

**Published:** 2020-12-31

**Authors:** Ana Coelho, Inês Amaro, Beatriz Rascão, Inês Marcelino, Anabela Paula, José Saraiva, Gianrico Spagnuolo, Manuel Marques Ferreira, Carlos Miguel Marto, Eunice Carrilho

**Affiliations:** 1 Institute of Integrated Clinical Practice, Faculty of Medicine, University of Coimbra, 3000-075 Coimbra, Portugal; ines.amaros@hotmail.com (I.A.); beatriznmfrascao@gmail.com (B.R.); inescarvalhomarcelino@gmail.com (I.M.); anabelabppaula@sapo.pt (A.P.); ze-93@hotmail.com (J.S.); cmiguel.marto@uc.pt (C.M.M.); eunicecarrilho@gmail.com (E.C.); 2 Area of Environment Genetics and Oncobiology (CIMAGO), Coimbra Institute for Clinical and Biomedical Research (iCBR), Faculty of Medicine, University of Coimbra, 3000-548 Coimbra, Portugal; m.mferreira@netcabo.pt; 3 Clinical Academic Center of Coimbra (CACC), 3004-561 Coimbra, Portugal; 4 Department of Neurosciences, Reproductive and Odontostomatological Sciences, University of Naples “Federico II”, 80131 Napoli, Italy; gspagnuo@unina.it; 5 Institute of Dentistry, I. M. Sechenov First Moscow State Medical University, 119435 Moscow, Russia; 6 Institute of Endodontics, Faculty of Medicine, University of Coimbra, 3000-075 Coimbra, Portugal; 7 Institute of Biophysics, Faculty of Medicine, University of Coimbra, 3004-548 Coimbra, Portugal; 8 Institute of Experimental Pathology, Faculty of Medicine, University of Coimbra, 3004-548 Coimbra, Portugal

**Keywords:** cavity disinfection, antimicrobial substances, chlorhexidine, adhesion, bonding, dental caries

## Abstract

Cavity disinfection becomes an important step before a dental restorative procedure. The disinfection can be obtained cleaning the dental cavity with antimicrobial agents before the use of adhesive systems. The aim of this study was to conduct a systematic review on the effect of different cavity disinfectants on restorations’ adhesion and clinical success. A search was carried out through the Cochrane Library, PubMed, and Web of Science. In vitro and in situ studies reporting results on dentin bond strength tests, and clinical studies published until August 2020, in English, Spanish and Portuguese were included. The methodological quality assessment of the clinical studies was carried out using the Revised Cochrane risk-of-bias tool. Chlorhexidine could preserve adhesion to dentin. EDTA and ethanol had positive results that should be further confirmed. Given the significant lack of scientific evidence, the use of lasers, fluoridated agents, sodium hypochlorite, or other products as cavity disinfectants should be avoided. Chlorhexidine is a safe option for cavity disinfection with adequate preservation of adhesion to dentin. Moreover, future researches should be focused on the efficacy of these disinfectants against cariogenic bacteria and their best application methods.

## 1. Introduction

Dental caries is the most prevalent pathology in the oral cavity, affecting most of the world population. Caries results from the interaction between dental structure and microbial biofilm, highly organized and formed on its surface, being characterized by the alternating phenomena of demineralization and remineralization [1,2,3]. Under pathological conditions, demineralization overcomes remineralization, leading to the dissolution of hard tissues of the tooth, degradation of collagen fibers and impairment of the mechanical properties of dentin, resulting in caries [1,2,4].

In situations where remineralization is insufficient to resolve the pathology, the treatment of dental caries consists in the removal of infected tissue and subsequent rehabilitation. However, during the removal of decayed tissue, there is the possibility of remaining viable bacteria in the cavity, which can compromise the success of rehabilitation, causing the appearance of a recurrence. On the other hand, rehabilitation failure may be related to tooth and/or restoration fracture and secondary caries, which often occurs at the interface between restorative material and dentin [5,6,7,8].

Dentin is considered an intrinsically moist and heterogeneous tissue, which makes adhesion to this tissue a more sensitive adhesive technique when compared to enamel [2,9].

Despite the evolution of adhesive systems, it is known that, over time, the hybrid layer suffers degradation, causing loss of adhesive resistance, which influences the longevity of restorations. The degradation of the adhesive interface is related to several factors, such as oral fluids and bacteria present in situ, leading to degradation of polymers and other organic components. Thus, cavity disinfection becomes an important step prior to the restorative procedure. This is described as cleaning the dental cavity with antimicrobial agents before the use of adhesive systems, making it as innocuous as possible [10].

Among the available disinfectants, chlorhexidine is the most used one. However, despite its beneficial effects, its impact on adhesion is still unclear [3,10,11].

The aim of this study was to conduct a systematic literature review, through the analysis of articles on the effect of different methods of cavity disinfection on adhesion and clinical success of restorations. As so, the research questions were developed according to the PICO (Population, Intervention, Comparison, Outcome) framework—Table 1.

## 2. Results

### 2.1. Study Selection

**Figure 1:**
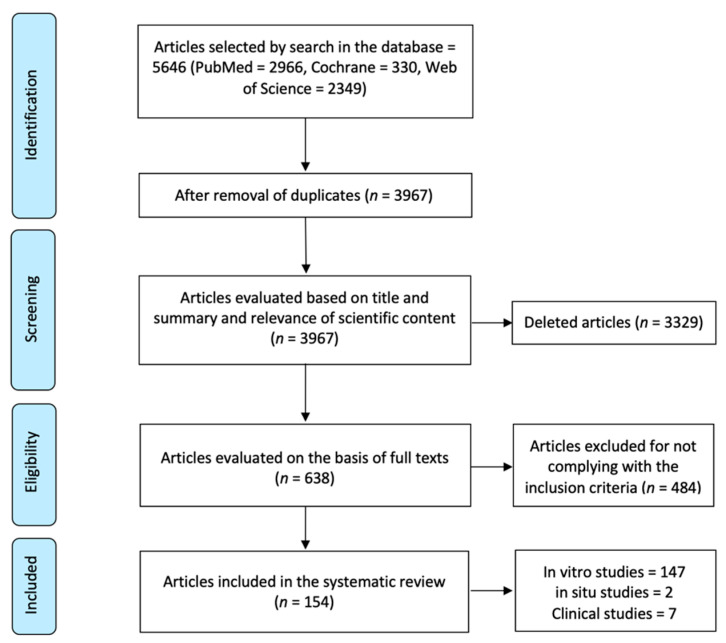
Flowchart of the selection process of the studies.

Initial screening of electronic databases yielded a total of 5645 articles. After removal of duplicated studies, a total of 3967 titles and abstracts were evaluated. Overall, a total of 638 potentially relevant articles were selected after an evaluation of titles and abstracts. Full text of these articles was obtained and thoroughly evaluated, and of these, 154 unique articles filled the inclusion criteria and were subsequently included in the systematic review. Two studies reported results for both in situ and in vitro experiments. The flowchart of the data selection process is presented in Figure 1.

### 2.2. In Vitro Studies

#### 2.2.1. Study Characteristics

One hundred and forty-seven in vitro studies were included in this review. Relevant information regarding each one of the studies was collected and can be found in Supplementary Materials S1.

The earliest study was published in 1992 [12] and the most recent ones were published in 2020 [13,14,15,16,17,18].

Almost all authors stated the use of premolars and/or permanent molars in all experimental procedures except for Yazici et al. [19] that only indicated the use of human teeth without specification on tooth type. The sample size ranged from 2 [20,21] to 20 [22] teeth per group.

From all of the selected teeth, only dentin substrates were put to use in the experimental protocols and the majority of the authors used healthy dentin as a test substrate. However, some of the authors also used infected dentin [14,23,24,25,26] or dentin presenting with artificial caries [27,28,29] made possible by a laboratory caries-induction procedure. In addition, some studies referred the use of deep and/or superficial dentin [30,31,32,33,34,35,36,37,38], however in most of the included studies this differentiation was not pointed out.

As regards to the storage medium to preserve teeth after extraction until its use in the experimental procedures, most of the authors reported the use of thymol [15,17,18,25,28,35,36,39,40,41,42,43,44,45,46,47,48,49,50,51,52,53,54,55,56,57,58,59,60,61,62,63,64,65,66,67,68,69,70,71,72,73,74,75,76,77,78,79], chloramine [13,14,23,26,27,29,30,32,33,34,38,80,81,82,83,84,85,86,87,88,89,90,91,92,93,94,95,96,97,98,99,100,101,102,103,104,105,106,107,108,109,110,111] or water [19,20,21,31,112,113,114,115,116,117,118,119,120,121,122,123,124,125,126,127,128,129,130,131]. However some other authors chose other storage solutions such as saline solution [22,24,37,132,133,134,135,136,137,138,139,140,141,142,143,144], sodium azide [22,135,136,137,145,146], alcohol [12], sodium hypochlorite [147] or formaldehyde [148]. In nine of the included studies [149,150,151,152,153,154,155,156,157] there was no available information regarding this issue.

The majority of the authors [12,13,14,15,16,17,18,19,20,21,23,25,26,27,28,30,31,32,33,34,35,36,38,39,40,41,43,44,45,46,47,48,50,51,52,53,54,55,56,58,59,60,61,63,64,65,67,68,69,70,71,73,74,75,76,77,78,79,80,81,82,83,84,85,86,87,88,89,90,91,92,93,94,95,96,97,99,100,101,102,103,104,105,106,107,108,109,110,112,113,114,115,116,117,118,119,120,121,122,123,124,126,127,128,129,130,131,132,133,134,135,136,137,139,140,141,142,143,144,145,146,147,148,149,152,154,156,157,158] reported the use of water as a storage medium or the incubation of teeth with a 100% humidity after experimental bonding procedures and before bond strength tests. In some of the studies other options for storage were considered such as artificial saliva [24,29,49,57,62,111,150], saline solution [37,125] and sodium hypochlorite [22]. Six authors [66,72,124,138,151,155] did not provide information on this matter. One of the authors, Silva et al. [98], conducted a study on the effect of chlorhexidine and storage media on bond strength values. The authors used distilled water, mineral oil and sodium hypochlorite as storage media to create three independent groups for each experimental procedure.

#### 2.2.2. Interventions—Cavity Disinfection Methods

Most of the studies reported results on the application of cavity disinfectant agents such as chlorhexidine [12,13,14,15,16,17,23,24,26,27,36,39,42,53,56,57,61,62,65,66,67,68,69,70,72,73,75,77,79,81,86,87,88,90,91,98,100,102,103,104,105,111,113,122,123,124,126,128,132,133,134,135,137,142,143,147,148,152,153,154,155,157], laser systems [14,19,20,21,22,28,29,31,33,37,38,40,41,43,44,46,49,50,51,52,54,58,60,63,71,76,78,80,82,83,88,89,94,95,96,101,107,112,114,115,116,117,119,120,125,131,139,140,141,143,144,145,151,156] and sodium hypochlorite (NaOCl) [18,22,25,30,32,34,35,45,47,64,66,70,74,85,90,97,98,106,110,115,118,121,129,130,132,138,149,150,152,154,158]. Further disinfectant solutions were also evaluated in some of the studies: EDTA based solutions [12,23,39,84,100,110,118,130,132,147], fluoridated agents [28,55,69,74,99,106], green tea extract/epigallocatechin gallate (EGCG)/catechin [56,57,67,136,137], ozone [26,48,93,133,143] and ozonated water [93], ethanol [26,59,61,75], tetracyclines [103,104,132,153], hydrogen peroxide [152,154], hypochlorous acid (HOCl) [25,47], boric acid [133,157], silver/zinc/titanium nanoparticles [73,108], aloe vera [65,134], urushiol [66,70], iodine based solutions [127,147,155], proanthocyanidin [53], ferrous sulfate (FeSO_4_) [57], benzalkonic chloride [100], grape seed extract [109] and glutaraldehyde based solutions (Gluma^®^, Kulzer, Hanau, Germany) [13,146].

#### 2.2.3. Effects of Interventions—Outcomes

##### Chlorhexidine

The studies evaluating the effects of chlorhexidine as a cavity disinfectant method tested different concentrations, ranging from 0.002% [86] to 5% [23,24]. However, most of the authors evaluated a 2% concentration [13,14,15,16,24,26,27,36,39,42,53,56,57,61,62,65,66,67,68,69,70,73,75,77,79,81,87,88,90,91,98,100,102,103,104,105,111,122,123,124,126,128,132,133,134,135,137,142,143,147,148,152,153,154,155,157].

Regarding its performance as a cavity disinfectant, chlorhexidine presented with positive results in the majority of the studies [16,17,24,36,39,42,53,56,57,61,62,65,66,67,68,69,72,73,75,77,79,81,86,87,90,91,98,100,102,103,104,105,111,113,122,123,124,128,132,134,135,137,142,143,148,153,157], allowing for maintenance or even an increase in bond strength values. On the other hand, a few authors [14,15,26,27,133,154,155] reported undesirable results when using chlorhexidine translating in a decrease in bond strength.

Some studies also evaluated the effects of chlorhexidine when combining it with different adhesive systems. Three of the authors [88,147,152] evaluated the effect of pretreatment with chlorhexidine before the application of two different adhesive systems and all of them reported a decrease in bond strength values when using a self-etch system and an increase or maintenance of values when using an etch-and-rinse system when comparing to control groups. On the contrary, Elkassas et al. [132] stated a decrease in bond strength values when using the etch-and-rinse system and an increase when using the self-etch system. However, five studies [79,113,122,128,137] did not find differences in bond strength when comparing the application of self-etch and etch-and-rinse adhesive systems. Sharma et al. [147] still reported that the use of 2% chlorhexidine before the application of a self-etch adhesive decreased bond strength values but when reducing the concentration to 1% the authors reported an increase in adhesive forces. Universal adhesive systems were also tested in some of the studies, presenting with positive results regarding bond strength. Say et al. [39] and Campos et al. [42] did not find differences between the use of an etch-and-rinse and a universal system as well as Akturk et al. [157] who also did not find differences between a self-etch and a universal system. Bravo et al. [123,124] also conducted studies testing the three different adhesive systems—etch-and-rinse, self-etch and universal—but find no differences between them. One other author tested the same universal system either as a self-etch or as an etch-and-rinse and concluded that bond strength values increased when using the universal system in etch-and-rinse mode and decreased when using it as a self-etch [102].

##### Laser Systems

Twenty-nine studies [19,28,29,33,37,38,41,43,44,54,58,78,80,82,83,89,94,95,107,112,115,116,117,119,125,140,141,144,145] reported the use of the ER:YAG laser, and six [14,50,88,89,96,131] the use of the Er,Cr:YSGG laser. Other laser systems such as the CO_2_ [20,49,71,151], diode [76,156], Nd:YAP [139], Excimer: KrF [46], ArF [114], Femtosecond [52] and the Ti:sapphire [119] lasers were also reported by some authors although to a lesser extent.

##### -ER:YAG LASER

Ten of the included studies [19,28,29,38,41,80,94,112,116,125] assessing the use of the ER:YAG laser reported a maintenance or increase in bond strength values, even when testing the laser with different working parameters [19,38,94,112]. However, Baraba et al. [95] tested different pulse durations—50, 100 and 300 µs—and reported worse adhesion results for all except for the medium short pulse (100 µs). Corona et al. [145] also compared different focal distances (mm) and concluded that adhesion values decreased for all except when the laser was used in a defocused mode (17 mm). Shirani et al. [58] tested the same variable—0.5, 2, 4, 11 mm—reporting positive results regarding bond strength but only for the 0.5 and 2 mm focal distances. Oliveira et al. [54] tested four laser settings (energy/repetition rate) but found no differences between them regarding bond strength alterations.

Nine studies [33,44,82,83,89,115,117,140,141] provided with negative results regarding adhesive forces when comparing to control groups and Gonçalves et al. [33] also reported a decrease in bond strength results when testing either on deep or superficial dentin.

Some of the studies also evaluated the effects of the ER:YAG laser combined with different adhesive procedures. Ramos et al. [78] and oliveira et al. [54] tested three different systems (self-etch, etch-and-rinse and universal) and reported that bond strength results were favourable when using the self-etch and universal adhesives but decreased when using the etch-and-rinse. Two studies [43,119] evaluated self-etch and etch-and-rinse adhesives being that in the study by Sierpinsky et al. [43], bond strength values decreased for all tested adhesives and in the one by Portillo et al. [119] there was only a decrease when using the etch-and-rinse system.

Two studies [94,144] tested the replacement of phosphoric acid with the laser and also tested its combined action and concluded that when the replacement was performed there was a decrease in adhesion but when used together bond strength values underwent no alterations. Davari et al. [144] also tested the combination of laser + phosphoric acid and reported worse results as well as Alahghemand et al. [37] who described similar results in deep dentin. Kucukilmaz et al. [107] also tested the replacement of phosphoric acid with laser and reported worse results when using the laser.

##### -Er,Cr:YSGG Laser

Three studies [88,96,131] reported favourable results regarding this intervention, presenting with the maintenance or increase in bond strength. Chou et al. [131] tested some different laser parameters but reported no differences in adhesion forces for none of them. Çelik et al. [88] tested two adhesive systems—self-etch and etch-and-rinse—applying the laser before the phosphoric acid in the etch-and-rinse system, and also reported positive results for both of them.

However, Ferreira et al. [89] reported negative results with a decrease in bond strength values when applying phosphoric acid followed by laser application. A significant decrease in adhesive forces was also reported by Carvalho et al. [50] who tested a self-etch and an etch-and-rinse adhesive making the replacement of phosphoric acid with the laser in the etch-and-rinse system.

##### -CO_2_ Laser

All four studies [20,49,71,151] assessing this laser as a cavity disinfectant agent reported a decrease in bond strength values when comparing to control groups.

##### -Nd:YAP Laser

As for the Nd:YAP laser system, only one study [139] evaluated its effects. The authors did not use any phosphoric acid nor adhesive system together with the laser in none of the experimental groups and reported an immediate decrease in bond strength.

##### -Diode

Only two studies [76,156] evaluated the diode laser as a disinfectant method. Zabeu et al. [156] evaluated two adhesive systems, concluding that regarding the etch-and-rinse system (where the laser was applied after the adhesive application) the bond strength values decreased immediately after the procedure and after 12 months. However, when testing the self-etch system these differences were not verified in neither one of the tested time periods. Kasraei et al. [76] reported opposite results since regarding the etch-and-rinse system, bond strength values did not decrease whether the laser was applied before or after the adhesive.

##### -Excimer: KrF, ArF

Regarding the Excimer: KrF laser, Eugénio et al. [46] reported a decrease in bond strength when the laser was used prior to the phosphoric acid and when it was used as a substitute of the phosphoric acid.

Regarding the Excimer: ArF laser, only one study was included [114] and the authors reported no alterations in bond strength values.

##### -Femtosecond

Regarding this intervention, only the study by Gerhardt et al. [52] was included in which different laser parameters were tested and concluded that there was a decrease in adhesive forces when using it at 80 µm/128 s. However, when using the laser as a substitute of the adhesive primer this decrease was not verified.

##### -Ti:sapphire

Regarding the Ti:sapphire laser, only one study was included, the one by Portillo et al. [119], in which three adhesive systems were tested—one etch-and-rinse and two self-etch—and the laser was always applied after the adhesive. Favourable results were only achieved when using the one-step self-etch adhesive.

##### Fluoridated Agents

Regarding the use of fluoridated agents, studies including the use of solutions such as silver diamine fluoride [28], ammonium hexafluorsilicate [28], sodium fluoride [69], Riva Star (SDI, Victoria, Australia) [99] and titanium tetrafluoride (TiF_4_) [55,74,106] were included.

##### -Silver Diamine Fluoride and Ammonium Hexafluorsilicate

As for these disinfection methods, only one study [28] evaluated both of them and concluded that bond strength values decreased when using either of them and when testing in both healthy and artificial caries-affected dentin.

##### -Sodium Fluoride

Only one study [69] evaluated this solution as a cavity disinfectant, reporting positive results regarding adhesive forces when used in a concentration of 1.23%.

##### -Titanium Tetrafluoride

Three studies [55,74,106] evaluated the effects of TiF_4_ as a disinfection method. Two of them [74,106] revealed positive outcomes, both using a 4% TiF_4_ concentration. In the third study, by Bridi et al. [55], two self-etch systems were tested with a 2.5% TiF_4_ concentration and with one of them there was a decrease of adhesive forces.

##### -Riva Star

The study by Koizumi et al. [99] was the only study including Riva Star. The authors reported a decrease in bond strength when using both self-etch and etch-and-rinse adhesive systems.

##### Sodium Hypochlorite

The studies evaluating the effects of NaOCl as a cavity disinfectant tested different concentrations, ranging from 0.5% [85,130] to 10% [18,32,35,45,110,115,121,150].

Regarding its action as a cavity disinfectant, NaOCl performed well in some of the studies, allowing for an increase and/or preservation of bond strength values [64,74,129,130]. Kunawarote et al. [25] also reported positive outcomes when testing either in healthy or infected dentin and Aguilera et al. [34] also did not find differences when testing deep nor superficial dentin when using a 5% NaOCl solution. However, two studies [32,35] revealed better results when testing a 10% NaOCl solution in superficial dentin while another study [30] reported an increase in bond strength when testing a 5% NaOCl solution in deep dentin.

Negative results regarding bond strength were also reported [18,45,70,106,150] and one of the studies [150] maintained these results even after 6 months. Kunawarote et al. [47] tested the effects of NaOCl in different application times (5, 15 and 30 s) and reported that as the application time increased, the results of adhesive forces worsened, with statistically significant results at 30 s.

Also, some of the studies tested the effect of NaOCl when used in combination with different adhesive systems, either self-etch [35,97,118], total-etch [35,115,121] and/or universal [118] and all reported positive results regarding bond strength. Abo et al. [85] even tested eight adhesive systems and reported an overall positive outcome immediately after the procedure as well as after 1 year.

Three authors [92,132,152] reported the use of two adhesive systems—self-etch and etch-and-rinse—and achieved opposite results since Mohammad et al. [92] reported a decrease in bond strength values when testing both adhesives, Elkassas et al. [132] reported an increase when using the self-etch and Ercan et al. [152] reported a decrease when using the self-etch. Two other authors [154,158] tested two self-etch adhesives and both also reported an overall decrease in bond strength results.

Prati et al. [138] tested four etch-and-rinse adhesives and reported a decrease in bond-strength for two of them when using a 1.5% NaOCl solution. Souza et al. [149] also tested four adhesives, two etch-and-rinse, one self-etch and one universal and reported a decrease in adhesive forces for only one of the etch-and-rinse.

In addition, Wuang et al. [110] reported that when testing a 5% NaOCl solution without phosphoric acid the results were unfavourable but when using both there was a positive outcome regarding bond strength. Also, Cha et al. [66] tested the influence of the use of NaOCl followed or not by a washing step and reported better results when the disinfectant was followed by washing.

##### EDTA Based Solutions

Of all the ten studies [12,23,39,84,100,110,118,130,132,147] evaluating the effects of the EDTA based solutions on adhesion, only Wang et al. [110] reported a decrease in bond strength associated with the use of such solutions. The authors tested a concentration of 15%.

##### Green Tea Extract/EGCG/Catechin

The green tea extract was evaluated by two studies [57,67] (at concentrations of 0.05% [57] and 2% [67]), the EGCG was evaluated by four studies [56,67,136,137] (at concentrations ranging from 0.02% [56,136] to 5% [137]) and the catechin by only one study [137]. Of all of the studies evaluating these solutions, only Santiago et al. [56] and Sun et al. [136] reported a decrease in bond strength associated with the use of EGCG but only in a 0.5% concentration.

##### Ethanol

Of all the four studies [26,59,61,75] evaluating the effects of ethanol as a disinfectant agent, only ozsoy et al. [26] described a decrease in bond strength but only when testing in caries-affected dentin.

##### Ozone

The use of ozone as a cavity disinfectant was reported in five of the included studies [26,48,93,133,143]. Garcia et al. [93] and Ercan et al. [133] did not report a decrease in bond strength regarding restorations performed in ozone disinfected cavities. However, Rodrigues et al. [48] reported a decrease in bond strength when the ozone was either used after or before the phosphoric acid application, describing a higher decrease in the latter. Dalkilic et al. [143] reported similar results as well as Ozsoy et al. [26] either in healthy or infected dentin.

##### Boric Acid

Boric acid was reported in two of the studies [133,157]. Akturk et al. [157], which evaluated its effects on adhesion when using it before a self-etch or a universal adhesive system, reported that there was a decrease in bond strength only when the boric acid was used before the universal system. However, Ercan et al. [133] reported a decrease in bond strength when testing the boric acid with a self-etch adhesive.

##### Iodine Based Solutions

Silva et al. [127], Sharma et al. [147] and Suma et al. [155] evaluated the effect of iodine based solutions on adhesion and all authors reported a decrease in bond strength in the majority of test conditions.

##### Hydrogen Peroxide

Two studies [152,154] evaluated the effects of 3% hydrogen peroxyde as a cavity disinfectant. Ercan et al. [152] evaluated the use of hydrogen peroxide when using an etch-and-rinse and a self-etch adhesive system. The authors described a decrease in bond strength associated with the use of the self-etch system. Reddy et al. [154] tested two self-etch systems and reported a decrease in bond strength for both adhesives.

##### Benzalkonic Chloride

Tekçe et al. [100] evaluated the effect of 1% benzalkonic chloride on adhesion 24 h and 12 months after the adhesive procedure. The authors reported a decrease in bond strength only at 12 months.

##### Other Disinfectant Methods

Urushiol [66,70], doxycycline [103,153], minocycline [104,153], glutaraldehyde based solutions (Gluma^®^) [13,146], hypochlorous acid [25,47], ferrous sulfate [57], proanthocyanidin [53], ozonated water [93], grape seed extract [109], silver/zinc/titanium nanoparticles [73,108] were all evaluated in only one or two studies and none of the products was associated with a decrease in bond strength in none of the test conditions. The effects of BioPure (Dentsply Sirona, York, PA, USA), a tetracycline based solution, were only reported by Elkassas et al. [132], who tested two distinct adhesive systems. The authors described an increase in bond strength after cavity disinfection with BioPure when used with an etch-and-rinse adhesive system. However, when using a self-etch system the bond strength decreased.

### 2.3. Clinical Studies

#### 2.3.1. Study Characteristics

Seven clinical studies were included in this review and relevant information regarding each one can be found in Supplementary Materials S2. All of the studies are classified as RCT being that four have a split-mouth design [159,160,161,162] and one is a pilot study [163].

The earliest study was published in 2006 [163] and the most recent one was published in 2020 [164].

The number of included participants ranged from 11 [163] to 42 [159,160] patients over 20 years old. The sample size ranged from 41 [163] to 169 [160] non-carious cervical lesions. All authors used relative field isolation except for Torres et al. [162] who used absolute field isolation. The follow-up period ranged from 6 months [160] to 5 years [162].

#### 2.3.2. Interventions—Cavity Disinfection Methods

All of the clinical studies selected for inclusion in this review were analyzed and four of them reported the use of 2% chlorhexidine [159,160,161,165], two reported the use of 10% NaOCl [162,163] and one reported the use of the diode laser [164].

#### 2.3.3. Clinical Outcomes

Several clinical aspects were assessed and used to withdraw conclusions regarding the effects of each disinfectant. All the clinical studies evaluated postoperative sensitivity, retention of the restoration and marginal discoloration. Presence or absence of secondary caries was evaluated by all authors except for Montagner et al. [160] and Favetti et al. [159]. Marginal integrity/adaptation was also evaluated by all except for Saboia et al. [163]. Pulp vitality was only evaluated in four of the included studies [159,160,161,162]. Other clinical aspects such as preoperative sensitivity [165], wear [165], clinical success [160,161], dental integrity [159,160], periodontal health [160] and survival rate [159] were only evaluated in one or two studies.

#### 2.3.4. Effects of Interventions—Outcomes

Dutra-Correa et al. [165], Montagner et al. [160] and Favetti et al. [159] tested a 2% chlorhexidine solution and didn’t find statistical differences at baseline and after follow-up regarding the clinical aspects assessed in each study. Saboia et al. [163] tested NaOCl at a 10% concentration and also reported no statistical differences regarding clinical outcomes either at 12 or 24 months. Torres et al. [162] also tested a 10% NaOCl solution and Akarsu et al. [164] tested a diode laser and both reported worse clinical results when comparing to baseline after 5 years and 18 months, respectively, however without statistically significant differences.

The only study presenting with statistically significant differences was the one by Sartori et al. [161] in which 2% chlorhexidine was evaluated. There was a statistically significant difference between the 12 and the 36 months recall for the experimental group regarding marginal discoloration. This difference was also verified between the baseline and the 36 months recall for both experimental and control groups. There was also a statistical difference between the baseline and the 36-month follow-up for the experimental group regarding retention and clinical success.

Of the four studies [159,160,161,162] evaluating pulp vitality, all reported a 100% success rate. The same percentage applied for the results regarding periodontal health, evaluated in only one study [160].

### 2.4. In Situ Studies

Only two in situ studies [61,122] were included in this review.

Simões et al. [61] evaluated the influence of chlorhexidine and ethanol on bond degradation. The authors compared the bond strength of an etch-and-rinse adhesive system at 24 h and 6 months (in vitro study) and in an in situ cariogenic challenge with nine patients aged 20–50 years. The bond strength values of the sticks submitted to the in situ cariogenic challenge were similar to those reported for the in vitro experience and the authors concluded that chlorhexidine and ethanol did not affect the bond strength.

Gunaydin et al. [122] evaluated the effect of 2% chlorhexidine on the immediate and aged dentin bond strength of one etch-and-rinse adhesive system and three self-etch adhesive systems in an in vitro and in an in situ experiment. For the in situ study, 40 patients aged 23–28 years were enrolled. Regardless of the adhesive system used, chlorhexidine treated groups exhibited lower immediate bond strength values and higher aged dentin bond strength values.

### 2.5. Assessment of Methodological Quality

**Figure 2:**
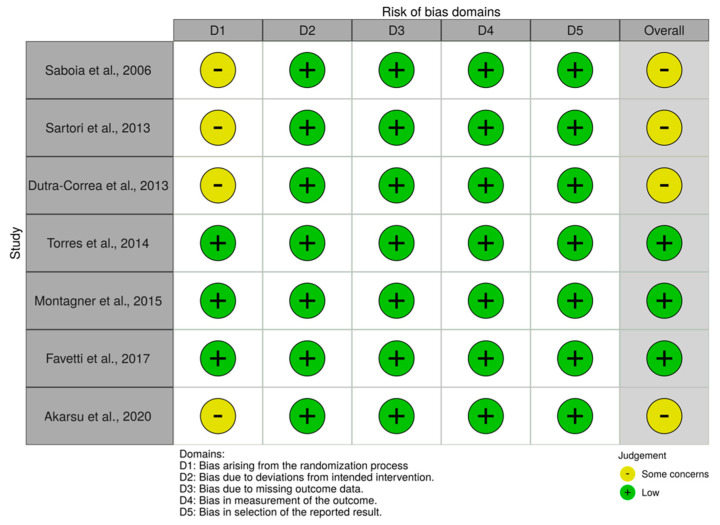
Methodological quality assessment of the included clinical (RCT) using the Revised Cochrane risk-of-bias tool for randomized trials (RoB 2).

The results of the studies’ quality assessment are presented in Figure 2.

Three studies presented an overall classification of “low” risk of bias while four studies presented with “some concerns”. These four studies all presented with insufficient information regarding the first domain as regards to the randomization process in which all of them failed to mention whether or not the allocation sequence was concealed until participants were enrolled and assigned to interventions. All of the other domains in all the included studies were well described.

## 3. Discussion

A cavity disinfectant must be bactericidal and/or bacteriostatic, biocompatible and easy to acquire and handle. It needs to be capable of correctly disinfecting the cavity but without compromising dentin bond strength. Its effect depends on each disinfectants characteristics but also on the type of substrate, adhesive system and restorative material used [90,108,126].

Dental substrates play an important role in the performance of adhesive systems, since the morphological and chemical-mechanical characteristics of healthy dentin are different from those of caries affected dentin [138]. The intertubular dentin of a caries affected substrate is partially demineralized, resulting in a softer and more porous structure, which compromises the adhesive strength [25,27,28]. Moreover, differences between superficial and deep dentin are also identified. Superficial dentin, composed mainly of intertubular dentin, has a higher percentage of collagen and a smaller number of dentinal tubules. The deep dentin, close to the pulp region, is formed mainly by dentinal tubules and presents a reduced percentage of intertubular dentin, mainly after acid etching [32,36,78,136]. As so, deep dentin is more hydrophilic, making disinfectants much more efficient in superficial dentin. In fact, several authors confirmed that adhesion to superficial dentin was significantly higher than that in deep dentin [30,31,32,33,37,38].

In most of the selected in vitro studies, the samples were placed in a storage medium before being submitted to adhesive resistance tests, in order to simulate the clinical aging of a material overtime. The ISO/TS 11405:2015 (Dental materials – testing of adhesion to tooth structure) [166] gives guidance on substrate selection, storage and handling of samples for quality testing of the adhesive bond between restorative materials and tooth structure. This ISO suggests distilled water or a 0.5% chloramine solution as good storage media for a maximum of one week after which the samples should be kept in distilled water at the temperature of 4 °C or under −5 °C. No other chemical agents should be used since it might affect absorption, adsorption, diffusion, and dissolution, and consequently alter the physical properties of dentin [167]. Furthermore, the longer the storage time, the worse the mechanical properties of the teeth (such as decreased microhardness and negative influence on bond strength) [168,169,170].

Although most studies reported the use of distilled water or chloramine as a storage medium, following the recommendations of the ISO, there were several authors using other solutions, such as thymol, which was used in 48 studies [15,17,18,25,28,35,36,39,40,41,42,43,44,45,46,47,48,49,50,51,52,53,54,55,56,57,58,59,60,61,62,63,64,65,66,67,68,69,70,71,72,73,74,75,76,77,78,79].

Given the degenerative changes that take place in dentin proteins, after teeth extraction the ISO/TS 11405:2015 [166] states that when it is not possible to perform experimental procedures immediately after teeth extraction, these should be performed in a time period not superior to 6 months. After the conclusion of all restorative procedures, the samples should be kept in water (ISO 3696:1987, grade 3 [171]) at a temperature of 23 °C.

The ISO/TS 11405:2015 [166] also states that ideally premolars and permanent molars should be used being also preferable to use third molars from individuals with ages ranging from 16–40 years. Almost all authors stated the use of premolars and/or permanent molars except for Yazici et al. [19] that only indicated the use of human teeth without specification on tooth type. However, it was not possible to obtain information regarding the age of the patients.

Regarding disinfectants, there are many available products in the market. In this review only studies that tested, at most, one cavity disinfection method per experimental group were included as a way to try to perceive each disinfectant’s true effect regarding bond strength alterations. In addition, experimental disinfectants were not considered.

### 3.1. In Vitro Studies

Chlorhexidine has a broad spectrum of antibacterial action, specially against gram-positive bacteria, and is used in different medical fields. Of all the species implicated in dental caries, epidemiologic evidence associates *Streptococcus mutans* as the main initiator of dental caries and it is known that chlorhexidine has a strong antibacterial capacity against it. Chlorhexidine also has the ability to inhibit the formation of the acquired pellicle, acting as an antiplaque agent [172,173,174,175,176,177,178].

As for the studies included in this review, most of the studies reported a maintenance or even an increase in bond strength values when chlorhexidine was used before the adhesive system, regardless of the concentration tested. Although some authors have studied the use of different adhesive systems (etch-and-rinse, self-etch and universal) after disinfection of the cavity with chlorhexidine, there seems to be no differences between them.

The enzymatic degradation of the collagen matrix by host-derived enzymes plays a significant role in destroying adhesive interfaces. Chlorhexidine is able to inhibit those collagen-degrading enzymes (namely matrix metalloproteinases (MMP) and cysteine cathepsins) which may justify the positive results [179,180,181]. On the other hand, chlorhexidine seems to have the ability to remove the loose smear debris and to increase the surface energy of dentin, which increases the wetting ability of primers [132,182].

The results are mainly positive and the pretreatment with chlorhexidine demonstrated an adequate preservation of adhesion to dentin, which makes it a safe option for cavity disinfection.

Another possible cavity disinfection method is laser irradiation. According to some authors, irradiation of the dentin surface results in improved microhardness, increased resistance to demineralization and decreased dentin permeability, minimizing bacterial access to the pulp [20,40,88].

However, the results regarding the use of lasers as cavity disinfectants are not consistent, with almost half of the studies reporting that lasers exert a negative effect on the mineral and organic components of dentin, impairing adhesion force [78,183,184].

The Erbium-doped yttrium aluminum garnet (Er:YAG) laser is widely used in different medical fields and has the highest water absorbency which makes it well absorbed in biological tissues containing water. This laser was the most studied one but the results are contradictory, regardless of the laser parameters and the adhesive system used. Dunn et al. [83] reported large peritubular areas with scaling and flaking aspects as well as a defective hybridization between dentin and composite resin when the surface was irradiated before acid-etching, which may justify the negative results. The residual thermal energy can also influence bonding by modifying the hydroxyapatite crystals, denaturing the collagen fibers and excessively dehydrating dentin [31,33,89,145,185,186].

Since laser ablation promotes opening of the dentinal tubules, some authors tested it as an alternative to acid-etching. However, bond strength values decreased in all studies [19,29,37,38,43,44,54,58,78,80,83,94,95,116,117,119,125,140,141,144]. One possible explanation for the negative results is that the Er:YAG laser doesn’t demineralize the peritubular dentin, which may hamper the formation of a hybrid layer and resin tags [31,187].

Regarding the other lasers included in this analysis (Er,Cr:YSGG, CO_2_, Nd:YAP, diode, Excimer: KrF and ArF, femtosecond, Ti:sapphire) the number of articles that reported results on them is very small. Since different lasers, with different parameters and under different conditions are used, it is not possible to draw conclusions.

Given the significant lack of scientific evidence, the use of lasers as cavity disinfectants should be avoided.

Some materials, such as fluoridated agents, are indicated for dentin pretreatment for different purposes [55]. These products help to diminish the development of secondary caries by decreasing dentin solubility and enhancing dentin remineralization [55,74]. Fluoridated agents such as silver diamine fluoride, ammonium hexafluorsilicate, sodium fluoride, titanium tetrafluoride (TiF_4_) and Riva Star were included in this review and its effects as cavity disinfectants prior to adhesive procedures and the influence in bond strength values were analysed. The results were mostly positive with reports of maintenance or even increase the bond strength in four out of six studies—sodium fluoride [69] and titanium tetrafluoride [55,74,106].

Metal fluorides, especially titanium tetrafluoride, have become popular due to their unique interaction with dental hard tissues [106]. In fact, TiF_4_ was the most studied fluoridated disinfectant agent, with a total of three studies which reported mostly positive results regarding bond strength. Its beneficial effects can be attributed to the increase of fluoride uptake, which can chemically reduce demineralization of dental hard tissues, but also to the formation of an acid-stable surface layer, referred to as glaze-like layer [188], which provides mechanical protection of the surface, forming a fine layer of titanium containing material, covering the surface and occluding the dentinal tubules [189,190]. However, one of the erosions’ inhibiting characteristics of TiF_4_ depends on its method of application on dentin. It seems that application with a microbrush leads to surface wear rather than allowing the formation of the glaze-like surface layer [191]. In all of the three included studies which tested TiF_4_, the product was actively applied with a microbrush for 60 s, which may possibly justify why pretreatment with TiF_4_ had no negative effects on bond strength.

Nevertheless, there was only a total of six studies regarding the use of fluoridated agents as cavity disinfectants and only three evaluating TiF_4,_ which makes it unadvisable to recommend its use until further studies are conducted on the matter to prove its feasibility.

Sodium hypochlorite is one of the most commonly used cavity disinfectants in clinical practice due to its antibacterial action and wettability property [132]. It also has the highest antimicrobial activity against anaerobic bacteria, as well as against *Streptococcus mutans* [66,192,193].

As regards to its use in restorative protocols, sodium hypochlorite has been considered for use prior to adhesive procedures as a cavity disinfectant. However, as for the studies’ results, there is a lack of consensus since some authors [64,74,129,130] reported positive results with maintenance or increase of bond strength values and others [18,45,70,106,150] reported a decrease. These differences might be attributed to differences in experimental protocols such as different NaOCl concentrations, adhesive systems and dentin substrates. Also, the decreased bond strength values may also be due to the oxygen released by NaOCl molecules as it may inhibit adhesive polymerization and compromise the mechanical performance of the bonding interfaces [32].

The substrate plays an important role on the adhesion of current adhesive systems and, in this case, four studies [30,32,34,35] evaluated the effects of sodium hypochlorite demineralization on deep and superficial dentin. Although one study [34] did not find differences when testing deep nor superficial dentin when using a 5% NaOCl solution, two other studies [32,35] revealed better results when testing a 10% NaOCl solution in superficial dentin. Since there are morphological differences between these two substrates, this might occur due to a decrease in the area of intertubular dentin available for bonding in deep dentin [30]. Furthermore, failure in removing all residual water confined deep into demineralized surfaces induces the formation of poorly polymerized polymer chains [34]. In addition, lower concentrations and times of application promote a continuous and slow denaturation of dentin collagen, leading to the formation of a gel layer on the demineralized dentin, preventing the diffusion of adhesive monomers. As the concentration and the time of application increases, denaturation progresses to degradation and complete dissolution of collagen, increasing the porosity of the dentin surface and diffusion of adhesive monomers through demineralized dentin [194]. This may also justify the results since a higher concentration of NaOCl—10%—was used in those two studies. Only one study [30] reported an increase in bond strength when testing in deep dentin with a 5% NaOCl solution. According to the authors, the total or partial removal of the collagen layer in the deep dentin by sodium hypochlorite may facilitate the penetration of the adhesive and avoid adhesive failures on deep cavities.

Some studies also evaluated the use of sodium hypochlorite in combination with different adhesive systems (etch-and-rinse, self-etch and/or universal) but there was no obvious consensus amongst authors since results were very incoherent making it impossible to draw conclusions regarding this matter.

There is no clear evidence on the effects on bond strength when using sodium hypochlorite as a cavity disinfectant prior to adhesive procedures and so caution is required until further studies assure its effects.

Ethylenediamine tetraacetic acid (EDTA) is an organic compound, capable of chelating calcium ions and selectively removing hydroxyapatite without entering deeply into the dentinal tubules [110]. It is an MMP inhibitor solution capable of increasing the longevity of the adhesive interface, dissolving the mineral components of dentin without altering the stability of the organic matrix and without causing collagen denaturation [23,84].

Nine [12,23,39,84,100,118,130,132,147] out of the ten studies evaluating the effects of EDTA reported maintenance or even an increase on mean bond strength despite the adhesive system used (etch-and-rinse, self-etch or universal). Although there is a need for further studies, the use of EDTA based solutions as cavity disinfectants prior to adhesive procedures seems to be a favorable alternative.

Ethanol is another possible alternative as a cavity disinfection method since when applied to dentin cavities it has the ability to expel water from dentin, keeping the collagen network distended. The interfibrillar spaces in the collagen matrix are filled with ethanol and the exposed collagen fibers are involved, preventing its degradation by MMP, thus providing a better substrate for resin placement and monomer infiltration [26,75]. It is also able to create a more hydrophobic environment, reducing water absorption over time, which is a key factor in the degradation of the adhesive bond [75]. This is in line with the results from the included studies since out of all the four studies [26,59,61,75] evaluating the effects of ethanol, only Ozsoy et al. [26] described a decrease in bond strength but only when ethanol was tested in caries-affected dentin. This substrate has a high degree of porosity due to mineral loss and partial or total tubular obstruction in the intertubular area which may justify these results. In addition, adhesive systems cannot infiltrate as deeply into demineralized intertubular structures as acids can, resulting in lower bond strength values than when testing in healthy dentin [26].

Since a decrease in bond strength was only observed when testing in caries-affected dentin [26] and since this is a more complex substrate for adhesion but also the main purpose of using cavity disinfectants, more studies should be conducted testing ethanol in caries-affected dentin to assess its feasibility when attempting to disinfect this substrate. Even though there is limited information about the use of ethanol as a cavity disinfectant, from the available results it looks like a promising alternative.

Ozone is a naturally occurring compound of three oxygen atoms. It is a strong oxidizing agent, with antibacterial activity capable of disrupting the cell wall and cytoplasmic membrane of microorganisms [157], which allows it to efficiently eliminate bacteria, fungi, protozoa, and viruses [48,143].

Ozone has also been proposed as a cavity disinfection method prior to restorative procedures since due to its oxidative and antimicrobial activity, it has the capacity to oxidize proteins in carious lesions and consequently diffusing and depositing calcium and phosphate ions into the demineralized dental tissues, hence leading to remineralization [48,143]. However, the results of the five included studies regarding the use of ozone are inconsistent since two authors [93,133] did not report a decrease in bond strength and the other three authors [26,48,143] reported opposite results even when testing either in healthy or infected dentin [26,143]. This might be due to the fact that ozone is an unstable molecule and rapidly dissociates into oxygen molecules which will react with free radicals and may cause the inhibition of polymerization of adhesive systems and thus reduce bond strength [26,48]. Therefore, the decrease in bond strength values may have been caused by the presence of residual oxygen molecules after the use of ozone. However, this is not in accordance with the results by Rodrigues et al. [48] who reported a higher decrease when ozone was used before rather than after the use of phosphoric acid which was immediately followed by the adhesive system. The authors stated that the results could only be justified by some chemical interaction that might have occurred between ozone and phosphoric acid, when the acid was used after the ozone, which interfered directly with the formation of the hybrid layer and might therefore affect the bond strength [48].

The use of ozone as a cavity disinfection method is still a matter of discussion since there is yet little available information on its effects on bond strength.

Some other disinfectant agents such as urushiol [66,70], tetracyclines [103,104,132,153], glutaraldehyde based solutions [13,146], hypochlorous acid [25,47], ferrous sulfate [57], proanthocyanidin [53], ozonated water [93], grape seed extract [109], silver/zinc/titanium nanoparticles [73,108], aloe vera [65,134] and green tea extract/EGCG/catechin [56,57,67,136,137] were included in this review, although to a lesser extent. Even though none of these products was associated with a decrease on bond strength in the majority of the test conditions, only a very limited number of studies regarding each disinfectant were included. On the other hand, other disinfectants such as boric acid [133,157], benzalkonic chloride [100], iodine based solutions [127,147,155] and hydrogen peroxide [152,154] were not only evaluated in very few studies but also all of them reported a decrease in bond strength in most of the experimental scenarios. Being that, the use of these products should be handled with caution since there is insufficient scientific evidence to support its use as cavity disinfectants.

In addition, there are several studies experimenting with different adhesive systems, given the different methodological approaches and reported results it is not possible to draw conclusions.

The development of new quality in vitro studies is essential. Although there is a lack of a standardized protocol, the experiments should account in detail for storage media and conditions, studied dental substrates, control and test groups, outcome measurement, and limited variables (e.g., the use of more than a cavity disinfectant per experimental group doesn’t allow the evaluation of its true effect on bond strength).

### 3.2. Clinical Studies

Concerning the clinical studies, the results were mainly positive for all tested disinfectants. Only Sartori et al. [161] reported worse results with statistically significant differences when testing a 2% chlorhexidine solution. The authors reported a statistically significant difference between the 12 and the 36 months recall for the experimental group regarding marginal discoloration and also between the baseline and the 36 months recall for both experimental and control groups. One possible explanation is the hypothesis that the repetitive cyclic of parafunctional loadings might have induced a failure in the cervical region of the restoration which might have generated micro-cracks at the restoration margin [161]. There was also a statistical difference between the baseline and the 36-month follow-up for the experimental group regarding retention and clinical success. These results might be due to the fact that despite chlorhexidine could decelerate the rate of resin-dentin bonds degradation by inhibition of MMP, it could not prevent the hydrolytic breakdown of polymers that constitute those bonds. Also, another aspect that could not be clarified in the study by Sartori et al. [161] is the origin of the adhesive failures, whether the negative results were due to a defect within the resin or the dentin matrix part of the hybrid layers.

Even though results concerning these disinfectants were mostly positive there is a clear need for further clinical studies regarding these and other disinfectant alternatives and with longer follow-up periods. According to the American Dental Association [195], it is required a retention rate of at least 90% of the restorations placed after 18 months to obtain full acceptance. Given this guideline, the study by Dutra-Correa et al. [165] achieved full acceptance since after 18 months the retention rate was over 90% when testing a 2% chlorhexidine solution. Torres et al. [162] reported a 97% retention rate after 18 months but not after 3 nor 5 years. Saboia et al. [163] also reported a 90% retention rate after 24 months but only for one of the tested adhesive systems.

According to the American Dental Association guidelines [195], non-carious cervical lesions are ideal models for testing the bonding of restorative materials to dental tissue. This is due to the non-carious loss of dental hard tissue having the most of the bonding area in dentinal tissue with only a small incisal/occlusal margin in enamel, they do not have a retentive shape and do not require preparation before restoration. However, the causes of the diminished longevity of non-carious class V restorations are still poorly understood, in contrast with other restorations [159,160,161] since they are known to have a multifactorial etiology and the prognosis may be significantly affected by several factors related to the material, patient and the environment [160]. The oral environment presents a challenge to the longevity of adhesive resistance since there are several factors that can influence the long term success of restorations such as temperature changes, masticatory load cycles, water absorption and pH fluctuations [196]. It is also important to consider that these kind of lesions might be due to characteristics of an individual patient (for example, parafunctional habits) [159,160,161] and so it is important to consider patient’s individual characteristics since these might affect the survival of restorations.

In order to reduce the impact of these variations on the studies, a good alternative might be to use a split-mouth design which was used in four of the included studies [159,160,161,162]. Also, different teeth cavity configurations [161] and each lesions’ different clinical characteristics [159,160] should also be taken into account since these might turn substrates more complex to adhesive procedures [159,160]—enamel, dentin or sclerotic dentin [159,160,165]. In the study by Montagner et al. [160] some cavity variables affected the retention of the cervical restorations regardless of the treatment since deeper and wider lesions presented statistically more retention failure than the others. On the other hand, the effect of cavity configuration and location of the restoration margin in relation to the gingival margin were not significant factors for restoration retention in the study by Favetti et al. [159].

A summary of the available evidence on each cavity disinfectant is presented in Table 2.

The main limitations of the included studies concerns the randomization process in which four of the studies [161,163,164,165] failed to mention whether or not the allocation sequence was concealed until participants were enrolled and assigned to interventions. Also, the field isolation is an important aspect to consider when performing adhesive procedures and since all authors used relative field isolation except for Torres et al. [162], who used absolute field isolation, this is also an important limitation. Restoration losses of non-carious cervical lesions are more prevalent in the posterior region due to stress intensity and moisture contamination in the cervical region which justifies the importance of absolute field isolation [164].

As so, there is a clear need for further clinical studies regarding this topic that allow the adoption of strict inclusion criteria, the generation of random sequences and the use of blind participants, examiners and evaluators. There is also a need for longer follow-up periods, in order to assess whether these products can be incorporated into restorative protocols or not.

## 4. Materials and Methods

This systematic review was registered on the International Prospective Register of Systematic Reviews (PROSPERO) platform (temporary ID: 199614) and designed according to PRISMA methodology (Preferred *Reporting Items for Systematic Reviews and Meta-Analysis*).

### 4.1. Inclusion and Exclusion Criteria

In vitro, in situ and clinical studies evaluating the effect of applying, at most, one cavity disinfection method per experimental group and evaluating the effect of the disinfection method on dentin adhesion of commercially available conventional adhesives and composites were included. Only in vitro and in situ studies reporting results for bond strength tests, presented in the form of mean and standard deviation, were considered.

Clinical studies on postoperative sensitivity, marginal pigmentation and adaptation, restoration loss, secondary caries, microinfiltration and pulp vitality were included.

Studies with no control group, in deciduous teeth, in bleached teeth or in teeth with endodontic treatment were excluded. Studies evaluating the adhesion of posts, cements, sealants, brackets or glass ionomer, studies that used manipulated adhesives (experimental or mixed with other solutions) and studies evaluating root or pulp chamber walls were also excluded. Regarding the laser, the studies that used it to prepare the cavity and not only as a disinfection method were also excluded. Previous contamination of teeth with hemostatic agents, saliva or blood was also considered an exclusion criterion.

All review articles, cell or animal studies, letters, case reports, abstracts and comments were excluded.

### 4.2. Search Strategy

An electronic search was carried out in the Cochrane Library (www.cochranelibrary.com), PubMed (www.ncbi.nlm.nih.gov/pubmed) and Web of Science (www.webofscience.com) databases, by articles published until August the 8th of 2020, without restrictions on the type, region or year of publication. Only articles in English, Portuguese and Spanish were included. Articles that weren’t available online were excluded.

For the research, the terms MeSH “Dentin”, “Disinfection”, “Anti-Bacterial Agents”, “Chlorhexidine”, “Sodium Hypochlorite”, “Lasers”, “Ozone”, “Ethanol”, “Aloe” and “Adhesives” were used. The search keys used in the different databases are found in Table 3.

### 4.3. Study Selection

The eligibility of the initially selected articles was evaluated by reading titles, abstracts and full text by two reviewers, independently. Any disagreement was discussed and the opinion of a third reviewer was obtained when necessary.

### 4.4. Data Extraction

Selected articles were read independently by two reviewers. During data extraction, two Microsoft^®^ Excel (Microsoft, Washington, WA, USA) tables were elaborated. The first, for the in vitro studies, included the parameters: name of the authors, year of publication, groups (n), storage solutions and materials used, and results (bond strength). The second table, for the in vivo studies, included the parameters: name of the authors, year of publication, type of study, groups (n), follow-up period, materials used, and results. Any disagreement was discussed and the opinion of a third reviewer was obtained when necessary.

### 4.5. Quality Assessment of the Included Clinical Studies

The methodological quality assessment of included clinical studies (randomized controlled trials, RCT) was assessed using the Revised Cochrane risk-of-bias tool for randomized trials (RoB 2) [197] by two independent reviewers. Briefly, five domains were evaluated: (D1) risk of bias arising from the randomization process; (D2) risk of bias due to deviations from the intended interventions; (D3) Risk of bias due to missing outcome data; (D4) Risk of bias in measurement of the outcome; (D5) Risk of bias in selection of the reported results. From this evaluation, each study may vary its overall classification regarding bias risk as “low”, “high” or “some concerns”.

## 5. Conclusions

A variety of different products is available for cavity disinfection prior to adhesive procedures. However, there are only a few that have been tested to a proper extent and with proved in vitro and clinical viability.

Chlorhexidine is a popular disinfectant and it was possible to conclude that it is a safe option for cavity disinfection since results are mainly positive with an adequate preservation of adhesion to dentin. Other disinfectants such as EDTA and ethanol may be promising alternatives but there is a clear need for further studies to safely suggest their use as cavity disinfectants. Also, further research is needed to clarify not only the effect of cavity disinfectants in bond strength but also their efficacy against cariogenic bacteria, their application times, products’ concentration, their use before or after acid-etching and their combination with different adhesive systems and dental substrates.

## Figures and Tables

**Table 1:** Population, Intervention, Comparison and Outcome (PICO) Strategy.

Parameter	In Vitro/In Situ	Clinical
**Population**	Teeth or dentin discs	Patients in need of a restoration
**Intervention**	Restoration with prior application of cavity disinfection methods	
**Comparison**	Conventional restoration	
**Outcome**	Effect of cavity disinfection on dentin adhesion (bond strength)	Effect of cavity disinfection on the clinical success of restoration

**Table 2:** Summary table of the available evidence on the use of cavity disinfectants.

Disinfectant	Does the Available Evidence Support Its Use as a Cavity Disinfectant?
Aloe vera	No (limited number of studies)
Benzalkonic chloride	No
Boric acid	No
Chlorhexidine	Yes
EDTA	Yes (positive results but limited number of studies)
Ethanol	No (limited number of studies)
Ferrous sulfate	No (limited number of studies)
Fluoride	Yes (positive results but limited number of studies)
Glutaraldehyde	No (limited number of studies)
Grape seed extract	No (limited number of studies)
Green tea extract/EGCG/catechin	No (limited number of studies)
Hydrogen peroxide	No
Hypochlorous acid	No (limited number of studies)
Iodine	No
Laser	No
Ozonated water	No (limited number of studies)
Ozone	No
Proanthocyanidin	No (limited number of studies)
Silver/zinc/titanium nanoparticles	No (limited number of studies)
Sodium hypochlorite	No
Tetracyclines	No (limited number of studies)
Urushiol	No (limited number of studies)

**Table 3:** Search keys used in the different databases. The asterisk represents any group of characters.

Database	Research
**Cochrane Library**	#1 MeSH descriptor: [Dentin] explodes all trees
	#2 dentin
	#3 cavity
	#4 MeSH descriptor: [Disinfection] in all MeSH products
	#5 disinfect*
	#6 antibacteria*
	#7 MeSH descriptor: [Anti-Bacterial Agents] explodes all trees
	#8 chlorhexidine
	#9 MeSH descriptor: [Chlorhexidine] explodes all trees
	#10 “sodium hypochlorite”
	#11 MeSH descriptor: [Sodium Hypochlorite] explodes all trees
	#12 laser
	#13 MeSH descriptor: [Lasers] explodes all trees
	#14 ozone
	#15 MeSH descriptor: [Ozone] explodes all trees
	#16 ethanol
	#17 MeSH descriptor: [Ethanol] explodes all trees
	#18 “aloe vera”
	#19 MeSH descriptor: [Aloe] explodes all trees
	#20 #1 OR #2 OR #3
	#21 #4 OR #5 OR #6 OR #7 OR #8 OR #9 OR #10 #11 OR #13 OR #12 #14 OR #15 OR #16 OR #17 OR #18 OR #19
	#22 adhesion
	#23 adhesive
	#24 MeSH descriptor: [Adhesives] in all MeSH products
	#25 bond strength
	#26 #22 OR #23 OR #24 OR #25
	#27 #20 AND #21 and #26
	#28 #20 AND #21
**Web of Science**	TS = ((dentin[MeSH Terms] OR dentin OR cavity) AND (disinfect* OR antibacteria* OR chlorhexidine OR “sodium hypochlorite” OR laser OR ozone OR ethanol OR “aloe vera”) AND (“bond strength” OR adhesion or adhesive))
**Pubmed**	(dentin[MeSH Terms] OR dentin OR cavity) AND (disinfection[MeSH Terms] OR disinfect* OR antibacteria* OR agents, antibacterial[MeSH Terms] OR chlorhexidine[MeSH Terms] OR chlorhexidine OR “sodium hypochlorite” OR sodium hypochlorite[MeSH Terms] OR laser OR lasers[MeSH Terms] OR ozone OR ozone[MeSH Terms] OR ethanol OR ethanol[MeSH Terms] OR “aloe vera” OR aloe[MeSH Terms]) AND (“bond strength” OR adhesion OR adhesive OR adhesives[MeSH Terms])

## Data Availability

The data presented in this study are available in Supplementary Materials S1 and S2.

## Supplementary Materials

Can be found at https://www.mdpi.com/1422-0067/22/1/353/s1.

## Abbreviations

CHX	Chlorhexidine
DT	Diamond tip
DTUS	Ultrasonic diamond tip
EDTA	Ethylenediamine tetraacetic acid
EGCG	Epillocatechin gallate
MSP	Medium short pulse
PBS	Phosphate buffer saline
QSP	Quantum square pulse
TC	Thermocyling
