# Supplementary material for: Effect of Cavity Disinfectants on Dentin Bond Strength and Clinical Success of Composite Restorations—A Systematic Review of In Vitro, In Situ and Clinical Studies
Source: Int J Mol Sci. 2020 Dec 31;22(1):353. doi: 10.3390/ijms22010353 (PMC7794949; doi:10.3390/ijms22010353)
Supplement: Supplementary file 1 [file ijms-22-00353-s001.zip › Supplementary material S1.docx]

Supplementary material S1 – Results from the *in vitro* studies included in the systematic review

| Authors, year | Groups (n) | Teeth | Storage | Materials | Strength bond | |
| --- | --- | --- | --- | --- | --- | --- |
| Gwinnett et al 1992[12] | 10% phosphoric acid +  G_1_: Tubulicid Red + wash + dry (10)  G_2_: Tubulicid Red (10)  G_3_: 4% CHX + wash + dry (10)  G_4_: 4% CHX (10)  G_5_: wash + dry (10)  G_6_: blotted dry (10)  + adhesive + resin | Third molars | 70% Alcohol + water (24h) | Tubulicid Red (Global Dental Products, USA)  Adhesive: All-Bond 2 (Bisco, USA)  Resin: Filtek P50 (3M, USA) | G_1_: 26.36±9.05; G_2_: 31.51±5.01;  G_3_: 27.11±4.98; G_4_: 31.85±4.61;  G_5_: 23.26±6.30; G_6_: 32.68±7.12  G_1_/G_3_/G_5_ > G_2_/G_4_/G_6_^*^ | |
| Perdigão et al, 1994[39] | 10% phosphoric acid +  G_1_: wash + dry + wet cotton + air blow (10)  G_2_: 2% CHX (10)  G_3_: dry + 2% CHX (10)  + adhesive + resin + TC | Molars | Thymol + distilled water  (1 week) | Adhesive: All-Bond 2  Resin: Filtek Z100 (3M, USA) | G_1_: 20.67±6.45; G_2_: 22.88±8.59;  G_3_: 21.70±7.12 | |
| Visuri et al, 1996[80] | G_1_: laser (9)  G_2_: laser + 10% phosphoric acid (9)  G_3_: none (9)  G_4_: 10% phosphoric acid (9)  + adhesive + resin | Third molars | 1% Chloramine  (3 months) | Laser Er:YAG (1-2-3, Schwartz eletro-optics, USA)  Adhesive: OroBond (Dentsply, Germany)  Resin: TPH (Dentsply, Germany) | G_1_: 12.9±7.3; G_2_: 7.1±5.0;  G_3_: 8.1±4.1; G_4_: 7.3±4.3  G_1_/All ^*^ | |
| Gürgan et al, 1999[81] | G_1_: 35% phosphoric acid (16)  G_2_: 2% CHX + 35% phosphoric acid (16)  G_3_: 35% phosphoric acid + 2% CHX (16)  G_4_: 35% phosphoric acid + 2% CHX + wash (16)  + adhesive + resin + TC | Third molars | 1% Chloramine (≤ 6 months) | Adhesive: Permagen A&B (Ultradent, USA) Resin: Tetric (Vivadent, Liechtenstein) | G_1_: 18.38±3.07; G_2_: 11.45±3.79;  G_3_: 12.41±3.79; G_4_: 18.04±3.85  G_1_/G_2_;G_3_; G_2_/G_4_^*^; G_3_/G_4_^*^ | |
| Prati et al, 1999[132] | 35% phosphoric acid +  G_1_: adhesive 1 (6) G_2_: 1.5% NaOCl (2 min) + adhesive 1 (6) G_3_: adhesive 2 (6) G_4_: 1.5% NaOCl (2 min) + adhesive 2 (6) G_5_: adhesive 3 (6) G_6_: 1.5% NaOCl (2 min) + adhesive 3 (6) G_7_: adhesive 4 (6) G_8_: 1.5% NaOCl (2 min) + adhesive 4 (6)  + resin | Third molars | Saline  (≤ 1 month) | Adhesive: 1 - Optibond FL (Kerr, USA); 2 - Prime & Bond 2.0 (Dentsply, Germany); 3 - Single Bond (3M, USA); 4 - Scotchbond Multi-purpose Plus (3M, USA) Resin: Filtek Z100 | G_1_: 12.7±2.5; G_2_: 17.9±4.4;  G_3_: 10.3±2.8; G_4_: 11.3±1.8;  G_5_: 14.9±3.9; G_6_: 10.6±5.3;  G_7_: 16.1± 10.7; G_8_: 9.6±7.4  G_1_/G_2_^*^; G_5_/G_6_^*^; G_7_/G_8_^*^ | |
| El-Housseiny et al, 2000[112] | G_1_: pumice paste (18)  G_2_: 2% CHX (18)  + 37% phosphoric acid + adhesive + resin | Premolars | Distilled water | Adhesive: Scotchbond MP plus  Resin: Filtek Z100 | G_1_: 8.12±3.54; G_2_: 8.22±2.10 | |
| Ceballos et al, 2002[31] | G_1_: 35% phosphoric acid (superficial dentin) (10) G_2_: 35% phosphoric acid (deep dentin) (10) G_3_: laser (superficial dentin) (10) G_4_: laser (deep dentin) (10) G_5_: laser + 35% phosphoric acid (superficial dentin) (10) G_6_: laser + 35% phosphoric acid (deep dentin) (10)  + adhesive + resin + TC | Third molars | Tap water (37°C, 24h) | Laser Er-YAG (Model 002532, KaVo, Germany)  Adhesive: Single Bond  Resin: Filtek Z100 | G_1_: 22.5±3.4; G_2_: 23.4±5.5;  G_3_: 4.0±2.2; G_4_: 6.3±3.0;  G_5_: 16.7±2.9; G_6_: 13.0±3.2  G_1_/G_3_;G_6_^*^; G_2_/G_3_;G_4_;G_5_;G_6_^*^; G_3_/G_5_;G_6_^*^; G_4_/G_5_;G_6_^*^; G_5_/G_6_^*^ | |
| Gonçalves et al, 2002[82] | G_1_: laser + 37% phosphoric acid + adhesive 1 + resin 1 (7)  G_2_: 37% phosphoric acid + adhesive 1 + resin 1 (7)  G_3_: laser + 37% phosphoric acid + adhesive 2 + resin 2 (7)  G_4_: 37% phosphoric acid + adhesive 2 + resin 2 (7) | Third molars | 0.5% Chloramine | Laser Er:YAG (Fidelis, Fotona, USA)  Adhesive: 1 - Optibond Solo (Kerr, USA); 2 – Single Bond  Resin: 1 - Prodigy (Kerr, USA); 3 – Filtek Z100 | G_1_: 12.57±5.29; G_2_: 19.88±4.95;  G_3_: 14.11±3.76; G_4_: 19.58±7.50  G_1_/G_2_^*^ | |
| Ramos et al, 2002[40] | G_1_: adhesive 1 (5) G_2_: laser + adhesive 1 (5) G_3_: 37% phosphoric acid + adhesive 2 (5) G_4_: laser + 37% phosphoric acid + adhesive 2 (5) G_5_: 37% phosphoric acid + adhesive 3 (5) G_6_: laser + 37% phosphoric acid + adhesive 3 (5)  + resin | Molars | Thymol  (≤ 6 months) | Laser Er:YAG (Kavo, Germany)  Adhesive: 1 - Clearfil Liner Bond 2V (Kuraray, Japan); 2 - Excite (Vivadent, Liechtenstein); 3 - Gluma One Bond (Heraeus Kulzer, USA)  Resin – Filtek Z250 (3M, USA) | G_1_: 21.62±5.28; G_2_: 19.20±2.99;  G_3_: 19.45±3.84; G_4_: 15.02±2.21;  G_5_: 16.30±3.56; G_6_: 15.52±5.28  G_1_/G_4_;G_5_;G_6_^*^; G_2_/G_4_;G_5_;G_6_^*^; G_3_/G_4_;G_5_;G_6_^*^ | |
| Toledano et al, 2002[30] | 36% phosphoric acid +  G_1_: adhesive (superficial dentin) (10)  G_2_: adhesive (deep dentin) (10)  G_3_: 5% NaOCl + adhesive (superficial dentin) (10)  G_4_: 5% NaOCl + adhesive (deep dentin) (10)  + resin + TC | Third molars | 0.5% Chloramine  (1 month) | Adhesive: Prime&Bond 2.1 (Dentsply, Germany)  Resin: Prisma TPH (Dentsply, Germany) | G_1_: 9.75±3.29; G_2_: 6.18±1.48;  G_3_: 7.44±2.64; G_4_: 8.53±2.18  G_1_/G_2_;G_4_^*^; G_2_/G_3_;G_4_^*^ | |
| de Castro et al, 2003[41] | G_1_: 35% phosphoric acid + adhesive 1 (3) G_2_: 2% CHX + 35% phosphoric acid + adhesive 1 (3) G_3_: 35% phosphoric acid + 2% CHX + adhesive 1 (3) G_4_: 35% phosphoric acid + adhesive 2 (3) G_5_: 2% CHX + 35% phosphoric acid + adhesive 2 (3) G_6_: 35% phosphoric acid + 2% CHX + adhesive 2 (3) G_7_: adhesive 3 (3) G_8_: 2% CHX + adhesive 3 (3)  + resin + TC | Third molars | 0.2% Thymol | Adhesive: 1 - Prime & Bond NT (Dentsply, Germany); 2 - Single Bond; 3 - Clearfil SE Bond (Kuraray, Japan) Resin: Filtek Z100 | G_1_: 31±10; G_2_: 38±9;  G_3_: 41±15; G_4_: 32±12;  G_5_: 36±13; G_6_: 38±15;  G_7_: 39±10; G_8_: 39±10 | |
| Uceda-Gómez et al, 2003[32] | 32% phosphoric acid +  G_1_: none (superficial dentin) (3) G_2_: 10% NaOCl (superficial dentin) (3)  G_3_: none (deep dentin) (3) G_4_: 10% NaOCl (deep dentin) (3)  + adhesive + resin | Molars | 0.5% Chloramine | Adhesive: One Step (Bisco, USA) Resin: Filtek Z100 | G_1_: 35.44±12.3; G_2_: 28.82±12.7;  G_3_: 26.48±8.50; G_4_: 24.27±8.30  G_1_/All^*^ | |
| Huang et al, 2004[133] | G_1_: 35% phosphoric acid + adhesive 1 (7) G_2_: adhesive 2 (7) G_3_: laser (7)  G_4_: 35% phosphoric acid + adhesive 1 (TC, 500 cycles) (7) G_5_: adhesive 2 (TC, 500 cycles) (7) G_6_: laser (TC, 500 cycles) (7) G_7_: 35% phosphoric acid + adhesive 1 (TC, 1000 cycles) (7) G_8_: adhesive 2 (TC, 1000 cycles) (7) G_9_: laser (TC, 1000 cycles) (7) G_10_: 35% phosphoric acid + adhesive 1 (TC, 2000 cycles) (7) G_11_: adhesive 2 (TC, 2000 cycles) (7) G_12_: laser (TC, 2000 cycles) (7) G_13_: 35% phosphoric acid + adhesive 1 (TC, 3000 cycles) (7) G_14_: adhesive 2 (TC, 3000 cycles) (7) G_15_: laser (TC, 3000 cycles) (7)  + resin | Molars | Saline | Laser Nd:YAP (Lokki, France)  Adhesive: 1 - Scotchbond; 2 - Clearfil Liner Bond II (Kuraray, Japan) | G_1_: 18.2±4.9; G_2_: 12.6±3.0;  G_3_: 13.4±3.3; G_4_: 16.3±4.4;  G_5_: 12.1±4.7; G_6_: 12.5±3.2;  G_7_: 12.4±2.1; G_8_: 11.1±2.6;  G_9_: 10.9±2.4; G_10_: 10.6±2.8;  G_11_: 9.5±3.0; G_12_: 9.2±1.9;  G_13_: 9.1±1.4; G_14_: 7.8±1.8;  G_15_: 8.1±1.7  G_1_/G_2_;G_3_; G_4_/G_5_;G_6_^*^ | |
| Ramos et al, 2004[134] | G_1_: adhesive 1 (15) G_2_: laser + adhesive 1 (15) G_3_: 35% phosphoric acid + adhesive 2 (15)  G_4_: laser + 35% phosphoric acid + adhesive 2 (15)  G_5_: 20% phosphoric acid + adhesive 3 (15)  G_6_: laser + 20% phosphoric acid + adhesive 3 (15)  + resin | Molars | 0.9% Saline + 0.4% sodium azide (4°C) | Laser Er:YAG (Kavo Key Laser 2; Kavo, Germany)  Adhesive: 1 - Clearfil SE Bond; 2 - Single Bond; 3 - Gluma One Bond  Resin: Filtek Z250 | G_1_: 20.65±1.81; G_2_: 14.06±1.88;  G_3_: 18.36±1.48; G_4_: 16.19±1.90;  G_5_: 16.58±1.94; G_6_: 14.07±2.13  G_1_/All^*^; G_2_/G_3_;G_4_;G_5_^*^; G_3_/G_4_;G_6_^*^; G_4_/G_6_^*^; G_5_/G_6_^*^ | |
| Say et al, 2004[42] | G_1_: 32% phosphoric acid + adhesive 1 (7)  G_2_: 2% CHX + 32% phosphoric acid + adhesive 1 (7)  G_3_: Ultracid F + 32% phosphoric acid + adhesive 1 (7)  G_4_: 32% phosphoric acid + adhesive 2 (7)  G_5_: 2% CHX + 32% phosphoric acid + adhesive 2 (7)  G_6_: Ultracid F + 32% phosphoric acid + adhesive 2 (7)  + resin | Third molars | Thymol crystals + distilled water (4ºC) | Adhesive: 1 - One Step; 2 - Optibond Solo  Ultracid F (Ultradent, USA)  Resin: Valux Plus (3M, USA) | G_1_: 11.33±3.04; G_2_: 11.33±3.58;  G_3_: 10.87±3.87; G_4_: 15.36±4.47;  G_5_: 13.71±5.47; G_6_: 13.55±2.71 | |
| Corona et al, 2005[145] | G_1_ - none (10) G_2_ - laser (11mm) (10) G_3_ - laser (12mm) (10) G_4_ - laser (14mm) (10) G_5_ - laser (16mm) (10) G_6_ - laser (17mm) (10)  + 35% phosphoric acid + adhesive + resin | Molars | 0.4% Sodium azide | Laser Er:YAG Kavo Key Laser 2  Adhesive: Single Bond Resin: Filtek Z250 | G_1_: 18.02±2.09; G_2_: 9.91±3.34;  G_3_: 9.49±2.29; G_4_: 10.98±3.45;  G_5_: 10.56±1.93; G_6_: 17.05±2.31  G_1_/G_2_;G_3_;G_4_;G_5_;  G_6_/G_2_;G_3_;G_4_;G_5_^*^ | |
| Dunn et al, 2005[83] | G_1_: none (20)  G_2_: 37% phosphoric acid (20)  G_3_: laser (20)  + adhesive + resin + TC | Molars | 0.5% Chloramine  (6 months) | Laser Er:YAG (DELight laser system, Continuum, USA)  Adhesive: Adper Scotchbond Multi-purpose  Resin: Filtek Z250 | G_1_: 19.8±3.6; G_2_: 6.1±2.1;  G_3_: 3.4±2.1  G_1_/G_2_;G_3_^*^; G_2_/G_3_^*^ | |
| Koshiro et al, 2005[20] | G_1_: phosphoric acid + adhesive 1 (2)  G_2_: phosphoric acid + adhesive 2 (2)  G_3_: laser + phosphoric acid + adhesive 1 (3) G_4_: laser + phosphoric acid + adhesive 2 (3)  + resin | Premolars | Water (37ºC, 24h) | Laser CO_2_ (Opelaser, Japan)  Adhesive: 1 - Single Bond; 2 - Clearfil Mega Bond (Kuraray, Japan) Resin: Clearfil AP-X (Kuraray, Japan) | G_1_: 57.2±4,5; G_2_: 41.1±10.0;  G_3_: All specimens broke during specimen preparation;  G_4_: 4.8±6,7;  G_2_/G_4_^*^ | |
| Osorio et al, 2005[84] | G_1_: 35% phosphoric acid + adhesive 1 (4) G_2_: 0.1M EDTA + adhesive 1 (4) G_3_: adhesive 2 (4)  + resin | Third molars | 0.5% Chloramine  (≤ 1 month) | Adhesive: 1 - Adper Scotchbond 1 (3M, USA); 2 - Clearfil SE Bond Resin - Tetric Ceram | G_1_: 43.3±11.1; G_2_: 40.4±9,7;  G_3_: 45.3±9.9 | |
| da Silva et al, 2006[113] | G_1_: 35% phosphoric acid + adhesive 1 (3) G_2_: 35% phosphoric acid + adhesive 2 (3) G_3_: adhesive 3 (3) G_4_: adhesive 4 (3) G_5_: 2% iodine + 35% phosphoric acid + adhesive 1 (3) G_6_: 2% iodine + 35% phosphoric acid + adhesive 2 (3) G_7_: 2% iodine + adhesive 3 (3) G_8_: 2% iodine + adhesive 4 (3)  + resin | Molars | Water | Adhesive: 1 - Single Bond; 2 - Prime & Bond NT; 3 - Clearfil SE Bond; 4 - Opti-Bond FL Resin: Filtek Z100 | G_1_: 44.2±15.9; G_2_: 37.1±14.8;  G_3_: 36.3±12.9; G_4_: 39.5±14.7;  G_5_: 34.9±14.0; G_6_: 37.8±14.4;  G_7_: 24.8±13.3; G_8_: 23.2±12.7  G_1_/G_2_;G_5_;G_6_;G_7_;G_8_^*^; G_2_/G_3_;G_4_^*^; G_3_/G_5_;G_6_;G_7_;G_8_^*^; G_4_/G_5_;G_6_;G_7_;G_8_^*^ | |
| Souza et al, 2005[149] | G_1_: 35% phosphoric acid + adhesive 1 + resin 1 (6) G_2_: 37% phosphoric acid + 5% NaOCl + adhesive 1 + resin 1(6) G_3_: 34% phosphoric acid + adhesive 2 + resin 2(6) G_4_: 37% phosphoric acid + 5% NaOCl + adhesive 2 + resin 2(6) G_5_: 15% phosphoric acid + adhesive 3 + resin 3(6) G_6_: 37% phosphoric acid + 5% NaOCl + 15% phosphoric acid + adhesive 3 + resin 3(6) G_7_: 35% phosphoric acid + adhesive 4 + resin 4 (6) G_8_: 37% phosphoric acid + 5% NaOCl + 35% phosphoric acid + adhesive 4 + resin 4 (6) | Third molars | N/A | Adhesive 1 - Single Bond; 2 - Prime & Bond NT; 3 - One Coat Bond (Còltene, Switzerland); 4 - PQ1 (Ultradent, USA)  Resin 1- Filtek Z250; 2- Esthet X (Dentsply, USA); 3 - Fill Magic (Coltène, Deutschland); 4 - Vit-l-escence (Ultradent, USA) | G_1_: 60.70±10.18; G_2_: 39.08±10.79;  G_3_: 31.73±6.91; G_4_: 61.53±5.85;  G_5_: 54.30±8.40; G_6_: 51.23±11.32;  G_7_: 39.11±9.45; G_8_: 58.18±10.98  G_1_/G_2_;G_3_;G_7_^*^; G_2_/G_4_;G_8_^*^; G_3_/G_4_;G_5_;G_6_;G_8_^*^; G_4_/G_7_^*^; G_7_/G_8_^*^ | |
| Abo et al, 2006[85] | G_1_: 37% phosphoric acid + adhesive 1 (8)  G_2_: 37% phosphoric acid + 0.5% NaOCl + adhesive 1 (8)  G_3_: 35% phosphoric acid + adhesive 2 (8)  G_4_: 35% phosphoric acid + 0.5% NaOCl + adhesive 2 (8)  G_5_: adhesive 3 (8)  G_6_: 0.5% NaOCl + adhesive 3 (8)  G_7_: adhesive 4 (8)  G_8_: 0.5% NaOCl + adhesive 4 (8)  G_9_: adhesive 5 (8)  G_10_: 0.5% NaOCl + adhesive 5 (8)  G_11_: adhesive 6 (8)  G_12_: 0.5% NaOCl + adhesive 6 (8)  G_13_: adhesive 7 (8)  G_14_: 0.5% NaOCl + adhesive 7 (8)  G_15_: adhesive 8 (8)  G_16_: 0.5% NaOCl + adhesive 8 (8)  + resin (24h)  G_17_: 37% phosphoric acid + adhesive 1 (8)  G_18_: 37% phosphoric acid + 0.5% NaOCl + adhesive 1 (8)  G_19_: 35% phosphoric acid + adhesive 2 (8)  G_20_: 35% phosphoric acid + 0.5% NaOCl + adhesive 2 (8)  G_21_: adhesive 3 (8)  G_22_: 0.5% NaOCl + adhesive 3 (8)  G_23_: adhesive 4 (8)  G_24_: 0.5% NaOCl + adhesive 4 (8)  G_25_: adhesive 5 (8)  G_26_: 0.5% NaOCl + adhesive 5 (8)  G_27_: adhesive 6 (8)  G_28_: 0.5% NaOCl + adhesive 6 (8)  G_29_: adhesive 7 (8)  G_30_: 0.5% NaOCl + adhesive 7 (8)  G_31_: adhesive 8 (8)  G_32_: 0.5% NaOCl + adhesive 8 (8)  + resin (1 year) | Molars | 0.5% Chloramine + water | Adhesive: 1 - OptiBond FL; 2 – EXL (3M, USA); 3 – OptiBond Solo Plus;  4 – Clearfil SE Bond; 5 – AdheSE (Ivoclar Vivadent, Liechtenstein); 6 – iBond (Heraeus Kulzer, Germany); 7 – Adper Prompt L-Pop; 8 – Xeno (Dentsply, USA)  Resin: Filtek Supreme (3M, USA) | G_1_: 31±4.7; G_2_: 27±4.2;  G_3_: 20±7.6; G_4_: 16±6.4;  G_5_: 13±2.4; G_6_: 22±4.8;  G_7_: 39±4.4; G_8_: 30±7.4;  G_9_: 17±4.3; G_10_: 27±6.7;  G_11_: 10±4.5; G_12_: 15±3.9;  G_13_: 14±4.1; G_14_: 13±2.8;  G_15_: 26±5.4; G_16_: 26±5.6;  G_17_: 28±6.8; G_18_: 25±7.0;  G_19_: 22±7.1; G_20_: 16±4.7;  G_21_: 12±4.5; G_22_: 16±2.5;  G_23_: 30±10.0; G_24_: 28±9.6;  G_25_: 25±9.6; G_26_: 26±2.4;  G_27_: 16±4.0; G_28_: 16±7.5;  G_29_: 13±3.0; G_30_: 14±3.4;  G_31_: 20±5.8; G_32_: 17±4.7  G_5_/G_6_;G_22_^*^; G_6_/G_21_^*^; G_9_/G_10_;G_26_^*^; G_15_/G_31_;G_32_^*^; G_16_/G_31_;G_32_^*^; G_21_/G_22_^*^ | |
| Franke et al, 2006[43] | 35% phosphoric acid + adhesive +  G_1_: none (12)  G_2_: laser (5J/cm^2^) (4)  G_3_: laser (10J/cm^2^) (4)  G_4_: laser (50J/cm^2^) (4)  + resin | Molars | 0.1% Thymol | Laser Nd:YAG (Quanta-Ray GCR-11, Spectra-Physics, USA)  Adhesive: Single Bond  Resin: Filtek Z250 | G_1_: 48.14±17.06; G_2_: 56.88±15.43;  G_3_: 48.81±16.68; G_4_: 30.42±10.53  G_2_/G_4_^*^ | |
| Rolla et al, 2006[21] | G_1_: 35% phosphoric acid + adhesive 1 (2)  G_2_: adhesive 2 (2)  G_3_: adhesive 3 (2)  G_4_: laser + 35% phosphoric acid + adhesive 1 (2)  G_5_: laser + adhesive 2 (2)  G_6_: laser + adhesive 3 (2)  + resin | Third molars | Distilled water (6 months) | Laser Nd:YAG Pulse Master 1000 (American Dental Technologies, USA)  Adhesive: 1 - Single Bond; 2 - One Step Plus (Bisco, USA); 3 - Adper Prompt L-Pop (3M, USA)  Resin: Filtek Z250 | G_1_: 49.32±13.64; G_2_: 19.13±10.71;  G_3_: 13.78±3.84; G_4_: 47.34±9.65;  G_5_: 27.09±8.97; G_6_: 22.85±9.29  G_2_/G_5_^*^; G_3_/G_6_^*^ | |
| Gonçalves et al, 2007[33] | G_1_: none (superficial dentin) (10)  G_2_: laser (1Hz) (superficial dentin) (10)  G_3_: laser (2Hz) (superficial dentin) (10)  G_4_: laser (3Hz) (superficial dentin) (10)  G_5_: laser (4Hz) (superficial dentin) (10)  G_6_: none (deep dentin - 1 mm) (10)  G_7_: laser (1Hz) (deep dentin - 1 mm) (10)  G_8_: laser (2Hz) (deep dentin - 1 mm) (10)  G_9_: laser (3Hz) (deep dentin - 1 mm) (10)  G_10_: laser (4Hz) (deep dentin - 1 mm) (10)  G_11_: none (deep dentin - 1,5 mm) (10)  G_12_: laser (1Hz) (deep dentin - 1,5 mm) (10)  G_13_: laser (2Hz) (deep dentin - 1,5 mm) (10)  G_14_: laser (3Hz) (deep dentin - 1,5 mm) (10)  G_15_: laser (4Hz) (deep dentin - 1,5 mm) (10)  + 37% phosphoric acid + adhesive + resin | Third molars | 0.5% Chloramine + distilled water  (1 week) | Laser Er:YAG Kavo Key Laser 2  Adhesive: Single Bond  Resin: Filtek Z250 | G_1_: 18.33±63.78; G_2_: 8.83±62.81;  G_3_: 7.64±62.67; G_4_: 8.76±63.15;  G_5_: 8.74±62.48; G_6_: 15.40±63.09;  G_7_: 9.20±62.38; G_8_: 11.35±63.93;  G_9_: 8.96±62.65; G_10_: 5.60±63.45;  G_11_: 12.19±62.76; G_12_: 6.61±63.09;  G_13_: 10.87±65.21; G_14_: 8.46±63.77;  G_15_: 6.89±63.08  G_1_/G_2_;G_3_;G_4_;G_5_;G_7_;G_8_;G_9_;G_10_;G_11_;G_12_;G_13_;G_14_;G_15_^*^;  G_6_/G_2_;G_3_;G_4_;G_5_;G_7_;G_9_;G_10_;G_12_;G_14_^*^; G_10_/G_11_^*^; G_11_/G_15_^*^ | |
| Malta et al, 2007[44] | G_1_: none (4) G_2_: laser (60 mJ, 5 Hz) (4)  G_3_: laser (100 mJ, 5 Hz) (4)  + 37% phosphoric acid + adhesive + resin | Molars | Distilled water + 0.2% thymol  (≤3 months) | Laser Er:YAG (Twinlight, Fotona, Slovenia)  Adhesive: Single Bond  Resin: Filtek Z250 | G_1_: 26.30±4.50; G_2_: 21.16±6.01;  G_3_: 22.23±4.98 | |
| Bengtson et al, 2008[114] | G_1_: 37% phosphoric acid + adhesive 1 (10) G_2_: adhesive 2 (10) G_3_: 2% CHX + 37% phosphoric acid + adhesive 1 (10) G_4_: 2% CHX + adhesive 2 (10)  + resin | Third molars | Distilled water (24h) | Adhesive: 1 - Single Bond 2; 2 - Clearfil SE Bond Resin: Filtek Z250 | G_1_: 42.88±2.3; G_2_: 31.52±6.24;  G_3_: 40.14±2.91; G_4_: 34.41±6.92 | |
| de Carvalho et al, 2008[141] | G_1_: none  G_2_: laser (150 mJ, 90º, with contact) (10)  G_3_: laser (70 mJ, 90º, with contact) (10)  G_4_: laser (150 mJ, 90º, no contact) (10)  G_5_: laser (70 mJ, 90º, no contact) (10)  G_6_: laser (150 mJ, 45º, with contact) (10)  G_7_: laser (70 mJ, 45º, with contact) (10)  G_8_: laser (150 mJ, 45º, no contact) (10)  G_9_: laser (70 mJ, 45º, no contact) (10)  + adhesive + resin | Third molars | Saline | Laser Er:YAG (Osada, Japan)  Adhesive: Clearfil SE Bond  Resin: Clearfil AP-X | G_1_: 44.97±6.36; G_2_: 23.83±2.46;  G_3_: 30.26±2.57; G_4_: 35.29±3.74;  G_5_: 41.90±4.95; G_6_: 27.48±2.11;  G_7_: 34.61±2.91; G_8_: 37.16±1.96;  G_9_: 41.74±1.60  G_1_/All^*^; G_2_/All^*^; G_3_/G_4_;G_5_;G_7_;G_8_;G_9_^*^; G_4_/G_5_;G_6_;G_9_^*^; G_5_/G_6_;G_7_;G_8_^*^; G_6_/G_7_;G_8_;G_9_^*^; G_7_/G_9_^*^; G_8_/G_9_^*^ | |
| Erhardt et al, 2008[23] | G_1_: 35% phosphoric acid (10)  G_2_: 35% phosphoric acid (infected dentin) (10)  G_3_: 0.1M EDTA (10)  G_4_: 0.1M EDTA (infected dentin) (10)  G_5_: 35% phosphoric acid + 5% CHX (10)  G_6_: 35% phosphoric acid + 5% CHX (infected dentin) (10)  + adhesive + resin | Molars | 0.5% Chloramine  (4ºC, 1 month) | Adhesive: Scotchbond 1  Resin: Tetric Ceram | G_1_: 31.9 ±3.3; G_2_: 26.2 ±3.5;  G_3_: 28.1±4.2; G_4_: 24.9±5.8;  G_5_: 27.3±4.7; G_6_: 23.8±5.5 | |
| Fawzy et al, 2008[129] | G_1_: 37% phosphoric acid + adhesive 1 (5)  G_2_: adhesive 2 (5)  G_3_: 5.25% NaOCl + adhesive 2 (5)  G_4_: 5.25% NaOCl + 37% phosphoric acid + adhesive 1 (5)  + resin | Third molars | Distilled water  (1 month) | Adhesive: 1 - Excite; 2 - AdheSE  Resin: Tetric Ceram | G_1_: 4.06±1.35; G_2_: 4.42±1.36;  G_3_: 7.42±2.16; G_4_: 4.68±1.26  G_3_/G_4_^*^ | |
| Saboia et al, 2008[150] | 35% phosphoric acid +  G_1_: 10% NaOCl (10) G_2_: 10% NaOCl (6 months) (10) G_3_: none(10) G_4_: none (6 months) (10)  + adhesive + resin | Third molars | N/A | Adhesive: XP-Bond (Dentsply, Germany)  Resin: Filtek Z250 | G_1_: 18.9±5.8; G_2_: 10.1±2.7;  G_3_: 49.9±10.3; G_4_: 35.2±8.7  G_1_/All^*^; G_2_/All^*^; G_3_/All^*^; G_4_/All^*^ | |
| Sierpinksy et al, 2008[43] | G_1_: laser + 37% phosphoric acid + adhesive 1 (5)  G_2_: 37% phosphoric acid + adhesive 1 (5)  G_3_: laser + adhesive 2 (5)  G_4_: adhesive 2 (5)  G_5_: laser + 37% phosphoric acid + adhesive 3 (5)  G_6_: 37% phosphoric acid + adhesive 3 (5)  + resin + TC | Molars | 0.2% Thymol  (3 months) | Laser Er:YAG (Twinlight, Fotona, Slovenia)  Adhesive: 1 - Excite; 2 - Prompt L-Pop; 3 - Single Bond  Resin: Tetric Ceram | G_1_: 19.7±6.8; G_2_: 25.7±6.7;  G_3_: 10.6±4.2; G_4_: 13.7±7.2;  G_5_: 19.0±7.7; G_6_: 25.6±9.2  G_1_/G_2_;G_3_;G_4_;G_6_^*^; G_2_/G_3_;G_4_;G_5_^*^; G_3_/All^*^; G_4_/All^*^ | |
| Baseggio et al, 2009[45] | 35% phosphoric acid +  G_1_: none (20) G_2_: 10% NaOCl (20)  + resin + TC | Third molars | Thymol  (≤ 1 week) | Adhesive: Single Bond  Resin: Filtek Z100 | G_1_: 50.3812±3.4; G_2_: 39.1584±2.6  G_1_/G_2_^*^ | |
| Breschi et al, 2009[111] | 35% phosphoric acid +  G_1_: 0.2% CHX + adhesive 1 (24h)  G_2_: 2% CHX + adhesive 1 (24h)  G_3_: adhesive 1 (24h)  G_4_: 0.2% CHX + adhesive 2 (24h)  G_5_: 2% CHX + adhesive 2 (24h)  G_6_: adhesive 2 (24h)  G_7_: 0.2% CHX + adhesive 1 (6 months)  G_8_: 2% CHX + adhesive 1 (6 months)  G_9_: adhesive 1 (6 months)  G_10_: 0.2% CHX + adhesive 2 (6 months)  G_11_: 2% CHX + adhesive 2 (6 months)  G_12_: adhesive 2 (6 months)  G_13_: 0.2% CHX + adhesive 1 (12 months)  G_14_: 2% CHX + adhesive 1 (12 months)  G_15_: adhesive 1 (12 months)  G_16_: 0.2% CHX + adhesive 2 (12 months)  G_17_: 2% CHX + adhesive 2 (12 months)  G_18_: adhesive 2 (12 months)  + resin | Third molars | 0.5% Chloramine | Adhesive: 1 - Scotchbond 1 XT; 2 – XP Bond  Resin: Filtek Z250 | G_1_: 41.9±9.6; G_2_: 39.1±11.9;  G_3_: 49.9±9.5; G_4_: 38.3±8.9;  G_5_: 37.6±5.6; G_6_: 39.6±9.4;  G_7_: 35.0±9.7; G_8_: 34.8±8.6;  G_9_: 27.2±8.4; G_10_: 33.3±8.5;  G_11_: 32.2±7.9; G_12_: 26.5±7.3;  G_13_: 33.2±8.3; G_14_: 29.5±12.7;  G_15_: 20.1±5.4; G_16_: 26.5±10.9;  G_17_: 28.5±7.5; G_18_: 14.2±5.0  G_9_/G_1_;G_2_;G_3_;G_4_;G_5_;G_6_^*^  G_15_/All^*^  G_18_/All^*^ | |
| Campos et al, 2009[42] | G_1_: phosphoric acid + adhesive 1 (10)  G_2_: phosphoric acid + 0.2% CHX + adhesive 1 (10)  G_3_: phosphoric acid + 2% CHX + adhesive 1 (10)  G_4_: adhesive 2 (10) G_5_: 0.2% CHX + adhesive 2 (10)  G_6_: 2% CHX + adhesive 2 (10)  G_7_: phosphoric acid + adhesive 1 (6 months) (10) G_8_: phosphoric acid + 0.2% CHX + adhesive 1 (6 months) (10) G_9_: phosphoric acid + 2% CHX + adhesive 1 (6 months) (10) G_10_: adhesive 2 (6 months) (10) G_11_: 0.2% CHX + adhesive 2 (6 months) (10) G_12_: 2% CHX+ adhesive 2 (6 months) (10)  + resin + TC | Third molars | 0.1% Thymol solution  (≤ 3 months) | Adhesive: 1 - Single Bond; 2 - Clearfil Tri S Bond (Kuraray, Japan) Resin: Filtek Z250 | G_1_: 24.22±1.65; G_2_: 23.45±2.13;  G_3_: 23.69±2.80; G_4_: 21.59±2.84;  G_5_: 20.15±3.07; G_6_: 20.50±2.06;  G_7_: 13.65±1.78; G_8_: 17.87±2.75;  G_9_: 17.43±1.73; G_10_: 12.75±2.52;  G_11_: 12.68±2.61; G_12_: 15.96±1.42  G_1_/G_7_;G_8_;G_9_^*^; G_2_/G_7_;G_8_;G_9_^*^; G_3_/G_7_;G_8_;G_9_^*^; G_7_/G_8_;G_9_^*^; G_4_/G_10_;G_11_;G_12_^*^; G_5_/G_10_;G_11_;G_12_^*^; G_6_/G_10_;G_11_;G_12_^*^; G_10_/G_12_^*^ | |
| Chou et al, 2009[131] | G_1_: 37% phosphoric acid (10)  G_2_: laser (5W; 30s) (10)  G_3_: laser (2.5W; 30s) (10) G_4_: laser (2.5W; 60s) (10) G_5_: laser (1.5W; 30s) (10) G_6_: laser (1.5W; 100s) (10)  + adhesive + resin | Third molars | Distilled water (≤ 1 month) | Laser Er,Cr:YSGG (Millennium; Biolase Technology, USA) Adhesive: Single Bond 2 Resin: Filtek Z250 | G_1_: 19.06±4.06; G_2_: 15.53±5.27;  G_3_: 12.96±2.50; G_4_: 15.61±4.53;  G_5_: 12.35±3.62; G_6_: 14.31±3.66 | |
| Ercan et al, 2009[152] | G_1_: adhesive 1 (10) G_2_: 34% phosphoric acid + adhesive 2 (10)  G_3_: 2% CHX + adhesive 1 (10)  G_4_: 2% CHX + 34% phosphoric acid + adhesive 2 (10) G_5_: 2.5% NaOCl + adhesive 1 (10) G_6_: 2.5% NaOCl + 34% phosphoric acid + adhesive 2 (10) G_7_: 1% CHX + adhesive 1 (10) G_8_: 1% CHX + adhesive 2 (10) G_9_: 3% H_2_O_2_ + adhesive 1 (10) G_10_: 3% H_2_O_2_ + 34% phosphoric acid + adhesive 2 (10)  + resin | Third molars | N/A | Adhesive: 1 - Clearfil SE Bond; 2 - Prime & Bond NT Resin: Clearfil AP-X | G_1_: 20.87±3.94; G_2_: 20.83±3.79;  G_3_: 16.36±2.46; G_4_: 21.01± 2.10;  G_5_: 15.21± 2.36; G_6_: 20.87±2.52;  G_7_: 20.86±3.65; G_8_: 21.00±3.53;  G_9_: 15.02±2.87; G_10_: 20.97±2.26  G_1_/G_3_;G_5_;G_9_^*^; G_2_/G_3_;G_5_;G_9_^*^; G_3_/G_4_;G_6_;G_7_;G_8_;G_10_^*^; G_4_/G_5_;G_9_^*^; G_5_/G_6_;G_7_;G_8_;G_10_^*^; G_6_/G_9_^*^; G_7_/G_9_^*^; G_8_/G_9_^*^; G_9_/G_10_^*^ | |
| Hedayatollahnajafi et al, 2009[151] | G_1_: 35% phosphoric acid + laser 300Hz, 25-mm/s + water (10)  G_2_: laser (300Hz, 25-mm/s) + water (10)  G_3_: 35% phosphoric acid + laser (300Hz, 50-mm/s) + water (10)  G_4_: laser (300Hz, 50-mm/s) + water (10)  G_5_: 35% phosphoric acid + laser (300Hz, 50-mm/s) (10)  G_6_: laser (300Hz, 50-mm/s) (10)  G_7_: 35% phosphoric acid + water (10)  G_8_: water (10)  + adhesive + resin | Molars | N/A | Laser CO_2_ Impact 2500 (Light Machinery, United Kingdom)  Adhesive: Single Bond  Resin: Filtek Z250 | G_1_: 29.9±6.4; G_2_: 14.1 ±7.2;  G_3_: 21.3±5.5; G_4_: 17.3±3;  G_5_: 14.1±5.3; G_6_: 11.3±5.8;  G_7_: 39.0±5.4; G_8_: 5.1±8.0  G_1_/G_2_;G_4_;G_5_;G_6_;G_7_;G_8_^*^; G_2_/G_7_^*^; G_3_/G_6_;G_7_;G_8_^*^; G_4_/G_7_;G_8_^*^; G_5_/G_7_^*^; G_6_/G_7_^*^; G_7_/G_8_^*^ | |
| Loguercio et al, 2009[86] | 34% phosphoric acid +  G_1_: none (5)  G_2_: 0.002% CHX (5)  G_3_: 0.02% CHX (5)  G_4_: 0.2% CHX (5)  G_5_: 2% CHX (5)  G_6_: 4% CHX (6 months) (5)  G_7_: none (6 months) (5)  G_8_: 0.002% CHX (6 months) (5)  G_9_: 0.02% CHX (6 months) (5)  G_10_: 0.2% CHX (6 months) (5)  G_11_: 2% CHX (6 months) (5)  G_12_: 4% CHX (6 months) (5)  + adhesive 1 + resin  37% phosphoric acid +  G_13_: none (5)  G_14_: 0.002% CHX (5)  G_15_: 0.02% CHX (5)  G_16_: 0.2% CHX (5)  G_17_: 2% CHX (5)  G_18_: 4% CHX (6 months) (5)  G_19_: adhesive 1 (6 months) (5)  G_20_: 0.002% CHX (6 months) (5)  G_21_: 0.02% CHX (6 months) (5)  G_22_: 0.2% CHX (6 months) (5)  G_23_: 2% CHX (6 months) (5)  G_24_: 4% CHX (6 months) (5)  + adhesive 2 + resin | Third molars | 0.5% Chloramine + distilled water  (6 months) | Adhesive: 1 - Prime & Bond 2.1; 2 - Single Bond  Resin: Opallis (FGM, Brazil) | G_1_: 32.0±3.2; G_2_: 28.2±3.4;  G_3_: 29.9±5.3; G_4_: 30.9±4.7;  G_5_: 34.2±5.1; G_6_: 26.7±4.7;  G_7_: 21.3±2.4; G_8_: 25.1±3.2;  G_9_: 30.1±4.2; G_10_: 27.4±4.6;  G_11_: 31.3±4.1; G_12_: 21.1±3.5;  G_13_: 34.1±4.6; G_14_: 35.2±6.2;  G_15_: 30.2±4.3; G_16_: 34.2±4.1;  G_17_: 32.4±6.1; G_18_: 26.3±4.2;  G_19_: 24.2±5.4; G_20_: 31.1±5.3;  G_21_: 27.3±5.1; G_22_: 36.2±4.0;  G_23_: 28.3±3.5; G_24_: 24.3±4.2  G_1_/G_7_;G_12_^*^; G_2_/G_7_;G_12_^*^; G_3_/G_7_;G_12_^*^; G_4_/G_7_;G_12_^*^; G_5_/G_7_;G_12_^*^; G_7_/G_9_;G_10_;G_11_^*^; G_9_/G_12_^*^; G_10_/G_12_^*^; G_11_/G_12_^*^; G_13_/G_19_;G_24_^*^; G_14_/G_19_;G_24_^*^; G_15_/G_19_;G_24_^*^; G_16_/G_19_;G_24_^*^; G_17_/G_19_;G_24_^*^; G_19_/G_20_;G_22_;G_23_^*^; G_20_/G_24_^*^; G_22_/G_24_^*^; G_23_/G_24_^*^ | |
| Sauro et al, 2009[130] | G_1_: 37% phosphoric acid + adhesive 1 (5) G_2_: 37% phosphoric acid + 0.5% NaOCl (30s) + adhesive 1 (5) G_3_: 0.1M EDTA + adhesive 1 (5) G_4_: 37% phosphoric acid + adhesive 2 (5) G_5_: 37% phosphoric acid + 0.5% NaOCl (30s) + adhesive 2 (5) G_6_: 0.1M EDTA + adhesive 2 (5)  G_7_: 37% phosphoric acid + adhesive 1 (12% NaOCl storage) (5) G_8_: 37% phosphoric acid + 0.5% NaOCl (30s) + adhesive 1 (12% NaOCl storage) (5) G_9_: 0.1M EDTA + adhesive 1 (12% NaOCl storage) (5) G_10_: 37% phosphoric acid + adhesive 2 (12% NaOCl storage) (5) G_11_: 37% phosphoric acid + 0.5% NaOCl (30s) + adhesive 2 (12% NaOCl storage) (5) G_12_: 0.1M EDTA + adhesive 2 (12% NaOCl storage) (5)  + resin | Molars | Distilled water (≤1 month) | Adhesive: 1 – Scotchbond 1 XT; 2 - Optibond Solo Plus Resin: Filtek Supreme | G_1_: 58.2±12.4; G_2_: 60.3±17.2;  G_3_: 56.2±13.6; G_4_: 60.2±13.3;  G_5_: 61.5±9.01; G_6_: 63.1±11.5;  G_7_: 32.8±9.8; G_8_: 57.3±15.7;  G_9_: 50.5±16.6; G_10_: 41.8±14.3;  G_11_: 56.1±12.6; G_12_: 57.6±16.4  G_1_/G_7_;G_10_^*^; G_4_;G_7_^*^;  G_7_/G_8_;G_9_^*^; G_10_/G_11_;G_12_^*^ | |
| Stanislawczuk et al, 2009[87] | 37% phosphoric acid +  G_1_: adhesive 1 (7) G_2_: adhesive 2 (7) G_3_: 2% CHX + adhesive 1 (7)  G_4_: 2% CHX + adhesive 2 (7)  G_5_: adhesive 1 (6 months) (7) G_6_: adhesive 2 (6 months) (7) G_7_: 2% CHX + adhesive1 (6 months) (7) G_8_: 2% CHX + adhesive 2 (6 months) (7)  + resin | Molars | 0.5% Chloramine  (≤ 6 months) | Adhesive: 1 - Prime&Bond NT; 2 - Single Bond 2  Resin: Opallis | G_1_: 22.0±9.7; G_2_: 27.2±6.1;  G_3_: 21.9±4.7; G_4_: 31.1±3.1;  G_5_: 14.6±3.1; G_6_: 20.4±2.1;  G_7_: 23.4±2.1; G_8_: 31.1±2.6  G_1_/G_5_^*^; G_2_/G_6_^*^; G_3_/G_5_^*^; G_5_/G_7_^*^; G_6_/G_8_^*^ | |
| Yazici et al, 2009[19] | G_1_: 34.5% phosphoric acid (14)  G_2_: laser (10s) (14)  G_3_: none (14)  + adhesive + resin | Molars | 0.1% Thymol  (≤ 1 month) | Laser Er:YAG  Adhesive: Futura Bond NR (Voco, Germany)  Resin: Filtek Z250 | G_1_: 9.84±5.7; G_2_: 8.06±5.62;  G_3_: 14.44±6,23  G_1_/G_3_^*^; G_2_/G_3_^*^ | |
| Çelik et al, 2010[88] | G_1_: 35% phosphoric acid + adhesive 1 (5) G_2_: adhesive 2 (5)  G_3_: 2% CHX + 35% phosphoric acid + adhesive 1 (5) G_4_: 2% CHX + adhesive 2 (5)  G_5_: laser + 35% phosphoric acid + adhesive 1 (5) G_6_: laser + adhesive 2 (5)  + resin | Third molars | 0.5% Chloramine | Laser Er,Cr:YSGG (Waterlase; USA);  Adhesive: 1 - Single Bond 2; 2 - Clearfil SE Bond  Resin: Filtek Z250 | G_1_: 16.4±5.62; G_2_: 18.5±6.11;  G_3_: 23.4±5.10; G_4_: 14.8±5.29;  G_5_: 23.4±6.46; G_6_: 23.5±5.5  G_1_/G_3_;G_4_;G_5_;G_6_^*^;  G_2_/G_3_;G_4_;G_5_;G_6_^*^  G_3_/G_4_^*^; G_4_/G_5_;G_6_^*^ | |
| Dalli et al, 2010[113] | G_1_: 34% phosphoric acid + adhesive 1 + resin 1 (15) G_2_: 1% CHX + 34% phosphoric acid + adhesive 1 + resin 1 (15) G_3_: 37% phosphoric acid + adhesive 1 + resin 1 (15) G_4_: Adhesive 2 + resin 2 (15) G_5_: 1% CHX + adhesive 2 + resin 2 (15) | Molars | Tap water  (1 week) | Adhesive: 1 - Prime & Bond NT; 2 - Clearfil S3 Bond (Kuraray, Japan) Resin: 1 - Quixfil (Dentsply, Germany); 2 - Clearfil Majesty Posterior (Kuraray, Japan) | G_1_: 16.4±4.1; G_2_: 16.2±3.9;  G_3_: 13.0±4.5; G_4_: 11.9±2.7;  G_5_: 11.5±2.7  G_1_/G_3_;G_4_;G_5_^*^; G_2_/G_3_;G_4_;G_5_^*^; G_3_/G_4_;G_5_^*^ | |
| Carvalho et al, 2011[50] | G_1_: adhesive 1 (5) G_2_: 37% phosphoric acid + adhesive 2 (5) G_3_: laser + adhesive 1 (5) G_4_: laser + adhesive 2 (5)  + resin | Third molars | 0.05% Thymol (5°C,  ≤ 3 months) | Laser Er,Cr:YSGG l (Waterlase Millenium, Biolase, USA);  Adhesive: 1 - Clearfil SE Bond; 2 - Single Bond Plus | G_1_: 52.5±5.6; G_2_: 41.2±5.7;  G_3_: 21.9±4.7; G_4_: 7.9±1.1  G_1_/G_3_^*^; G_2_/G_4_^*^; G_3_/G_4_^*^ | |
| Chang et al, 2010[142] | G_1_: 2% CHX + wash + 37% phosphoric acid (24h) (5)  G_2_: 2% CHX + 37% phosphoric acid (24h) (5)  G_3_: 37% phosphoric acid + 2% CHX + wash (24h) (5)  G_4_: 37% phosphoric acid + 2% CHX (24h) (5)  G_5_: 37% phosphoric acid (24h) (5)  G_6_: 2% CHX + wash + 37% phosphoric acid (TC) (5)  G_7_: 2% CHX + 37% phosphoric acid (TC) (5)  G_8_: 37% phosphoric acid + 2% CHX + wash (TC) (5)  G_9_: 37% phosphoric acid + 2% CHX (TC) (5)  G_10_: 37% phosphoric acid (TC) (5)  + adhesive + resin | Molars | Saline | Adhesive: Single Bond 2  Resin: Filtek Z350 (3M, USA) | G_1_: 30.84±3.86; G_2_: 31.05±5.56;  G_3_: 29.97±6.81; G_4_: 29.35±3.82;  G_5_: 29.43±3.37; G_6_: 26.48±3.09;  G_7_: 26.19±2.66; G_8_: 25.86±3.16;  G_9_: 26.86±2.67; G_10_: 19.12±2.49  G_10_/All^*^ | |
| Eugénio et al, 2010[46] | G_1_: 35% phosphoric acid (4)  G_2_: laser (4)  G_3_: laser + 35% phosphoric acid (4)  + adhesive + resin | Third molars | 0.1% Thymol (≤1 month) | Laser KrF excimer Adhesive: Scotchbond 1 XT  Resin: Esthet-X | G_1_: 33.7±8.7; G_2_: 13.8±5.1;  G_3_: 19.7±6.9  G_1_/G_2_;G_3_^*^; G_2_/G_3_^*^ | |
| Ferreira et al, 2010[89] | G_1_: 35% phosphoric acid (15s) (10)  G_2_: 35% phosphoric acid (30s) (10)  G_3_: 35% phosphoric acid (60s) (10)  G_4_: 35% phosphoric acid (15s) + laser 1 (10)  G_5_: 35% phosphoric acid (30s) + laser 1 (10)  G_6_: 35% phosphoric acid (60s) + laser 1 (10)  G_7_: 35% phosphoric acid (15s) + laser 2 (10)  G_8_: 35% phosphoric acid (30s) + laser 2 (10)  G_9_: 35% phosphoric acid (60s) + laser 2 (10)  + adhesive + resin | Third molars | 0.5% Chloramine | Laser: 1 - Er:YAG (Kavo Key Laser III, Kavo, Germany); 2 - Er,Cr:YSGG (Waterlase Millenium, Biolase Technology, USA)  Adhesive: Single Bond  Resin: Filtek Z250 | G_1_: 22.30±4.25; G_2_: 21.54±4.71;  G_3_: 18.88±3.05; G_4_: 16.51±3.94;  G_5_: 15.74±3.40; G_6_: 12.62±5.90;  G_7_: 12.38±2.47; G_8_: 10.69±3.05;  G_9_: 8.8±4.36  G_1_/G_4_;G_7_^*^; G_2_/G_5_;G_8_^*^; G_3_/G_6_;G_9_^*^ | |
| Kunawarote et al, 2011[25] | G_1_: none (5) G_2_: 806.02 mM NaOCl (5)  G_3_: 0.95 mM HOCl (5) G_4_: 1.91 mM HOCl (5)  G_5_: none (infected dentin) (5) G_6_: 806.02 mM NaOCl (infected dentin) (5) G_7_: 0.95 mM HOCl (infected dentin) (5) G_8_: 1.91 mM HOCl (infected dentin) (5)  + adhesive + resin | Molars | Saline + thymol | Adhesive: Clearfil SE Bond Resin: Clearfil AP-X | G_1_: 41.56±5.39; G_2_: 40.87±5.33;  G_3_: 41.93±4.93; G_4_: 41.24±7.10;  G_5_: 26.77±3.47; G_6_: 31.62±6.62;  G_7_: 39.28±5.27; G_8_: 34.16±6.58  G_1_/G_5_^*^; G_2_/G_6_^*^; G_4_/G_8_^*^; G_5_/G_7_^*^ | |
| Kunawarote et al, 2010[47] | G_1_: none (3) G_2_: 6% NaOCl (5s) (3)  G_3_: 6% NaOCl (15s) (3)  G_4_: 6% NaOCl (30s) (3)  G_5_: HOCl (50ppm) (5s) (3)  G_6_: HOCl (50ppm) (15s) (3)  G_7_: HOCl (50ppm) (30s) (3)  G_8_: HOCl (100ppm) (5s) (3)  G_9_: HOCl (100ppm) (15s) (3)  G_10_: HOCl (100ppm) (30s) (3)  G_11_: HOCl (200ppm) (5s) (3)  G_12_: HOCl (200ppm) (15s) (3)  G_13_: HOCl (200ppm) (30s) (3)  + adhesive + resin | Molars | Saline + thymol | Adhesive: Clearfil SE Bond  Resin: Clearfil AP-X | G_1_: 41.26±6.32; G_2_: 40.34±5.77;  G_3_: 38.43±5.26; G_4_: 27.19±5.62;  G_5_: 41.97±4.94; G_6_: 37.64±6.35;  G_7_: 36.87±6.03; G_8_: 41.24±7.10;  G_9_: 37.38±6.64; G_10_: 36.26±5.23;  G_11_: 37.68±6.17; G_12_: 36.87±7.47;  G_13_: 35.39±8.84  G_4_/G_1_;G_2_;G_3_;G_5_;G_6_;G_8_;G_11_^*^ | |
| Ramos et al, 2010[112] | G_1_: 35% phosphoric acid + adhesive 1 (8)  G_2_: adhesive 1 (8) G_3_: adhesive 2 (8)  G_4_: laser (250 mJ / 2 Hz) + 35% phosphoric acid + adhesive 1 (8)  G_5_: laser (250 mJ / 2 Hz) + adhesive 1 (8) G_6_: laser (250 mJ / 2 Hz) + adhesive 2 (8) G_7_: laser (400 mJ / 4 Hz) + 35% phosphoric acid + adhesive 1 (8)  G_8_: laser (400 mJ / 4 Hz)+ adhesive 1 (8) G_9_: laser (400 mJ / 4 Hz) + adhesive 2 (8)  + resin | Third molars | Distilled water (37°C, 24h) | Laser Er:YAG KaVo Key Laser 2  Adhesive: 1 - Single Bond; 2 - Clearfil SE Bond Resin: Filtek Z250 | G_1_: 18.38±0.83; G_2_: 5.59±0.76;  G_3_: 17.51±2.37; G_4_: 18.03±1.60;  G_5_: 7.62±0.73; G_6_: 17.54±1.29;  G_7_: 18.19±1.04; G_8_: 7.63±0.75;  G_9_: 16.32±1.05  G_1_/G_2_;G_5_;G_8_^*^; G_2_/G_3_;G_4_;G_6_;G_7_;G_9_^*^; G_3_/G_5_;G_8_^*^; G_4_/G_5_;G_8_^*^; G_5_/G_6_;G_7_;G_9_^*^; G_6_/G_8_^*^; G_7_/G_8_^*^; G_8_/G_9_^*^ | |
| Yazıcı et al, 2010[19] | G_1_: adhesive 1 (10) G_2_: laser (3 Hz, 100 mJ) + adhesive 1 (10) G_3_: laser (15 Hz, 1 W) + adhesive 1 (10) G_4_: adhesive 2 (10) G_5_: laser (3 Hz, 100 mJ) + adhesive 2 (10) G_6_: laser (15 Hz, 1 W) + adhesive 2 (10)  + resin | N/A | Distilled water | Laser Er:YAG (Fidelis III, Fotona, Slovenia); Adhesive: 1 - Clearfil SE Bond; 2 - Clearfil Tri-S Bond  Resin: Clearfil Majesty Esthetic (Kuraray, Japan) | G_1_: 17.98±4.57; G_2_: 8.26±2.01;  G_3_: 10.16±2.14; G_4_: 10.93±1.72;  G_5_: 11.23±3.16; G_6_: 8.58±3.49 | |
| Mobarak et al, 2011[24] | G_1_: water (24h) (10)  G_2_: 2% CHX (24h) (10)  G_3_: 5% CHX (24h) (10)  G_4_: none (2 years) (10)  G_5_: 2% CHX (2 years) (10)  G_6_: 5% CHX (2 years) (10)  G_7_: water (24h) (infected dentin) (10)  G_8_: 2% CHX (infected dentin) (24h) (10)  G_9_: 5% CHX (infected dentin) (24h) (10)  G_10_: none (infected dentin) (2 years) (10)  G_11_: 2% CHX (infected dentin) (2 years) (10)  G_12_: 5% CHX (infected dentin) (2 years) (10)  + adhesive + resin (under simulated pulp pressure) | Molars | Saline  (2 weeks) | Adhesive: Clearfil SE Bond  Resin: Filtek Supreme XT | G_1_: 24.33±5.1 G_2_: 23.79±5.9;  G_3_: 25.94±6.4; G_4_: 9.46±3.4;  G_5_: 8.74±3.2; G_6_: 10.98±3.3;  G_7_: 21,73±6.0; G_8_: 20.84±6.2;  G_9_: 20.59±5.1; G_10_: 9.97±2.5;  G_11_: 9.99±3.4; G_12_: 14.67±4.5  G_1_/G_4_;G_10_^*^; G_2_/G_5_;G_11_^*^; G_3_/G_6_^*^; G_4_/G_7_^*^; G_5_/G_8_^*^; G_6_/G_9_;G_12_^*^; G_7_/G_10_^*^;G_8_/G_11_^*^; G_10_/G_12_^*^ | |
| Mohammad et al, 2011[90] | G_1_: adhesive 1 (15)  G_2_: 2% CHX + adhesive 1 (15)  G_3_: adhesive 2 (15)  G_4_: 2% CHX + adhesive 2 (15)  + resin + TC | Premolars | 0.5% Chloramine + distilled water  (1 week) | Adhesive: 1 - Clearfil SE Bond; 2 - Clearfil S3 Bond  Resin: Filtek Z100 | G_1_: 22.86±0.61; G_2_: 22.07±1.34;  G_3_: 22.13±1.12; G_4_: 21.74±2.78 | |
| Mohammad et al, 2011[92] | G_1_: 35% phosphoric acid + adhesive 1 (15) G_2_: 5.25% NaOCl + 35% phosphoric acid + adhesive 1 (15) G_3_: adhesive 2 (15) G_4_: 5.25% NaOCl + adhesive 2 (15)  + resin + TC | Third molars | 0.5% Chloramine + distilled water  (1 week) | Adhesive: 1 - Single Bond; 2 - Clearfil S3 Bond Resin: Filtek Z100 | G_1_: 32.38±5.78; G_2_: 28.12±3.95;  G_3_: 25.91±5.03; G_4_: 22.15±3.96  G_1_/G_2_^*^; G_3_/G_4_^*^ | |
| Nguyen et al, 2011[49] | G_1_: laser (25 mm/S) + water + 35% phosphoric acid (10)  G_2_: laser (25 mm/S) + water (10)  G_3_: laser (50 mm/S) + water + 35% phosphoric acid (10)  G_4_: laser (50 mm/S) + water (10)  G_5_: laser (50 mm/S) + 35% phosphoric acid (10)  G_6_: laser (50 mm/S) (10)  G_7_: 35% phosphoric acid (10)  G_8_: none (10)  + adhesive + resin | Molars | Gamma radiation + 0.1% thymol | Laser CO_2_ (Impact 2500, GSI Lumonics, United Kingdom)  Adhesive: Single Bond  Resin: Filtek Z250 | G_1_: 29.9±6.4; G_2_: 14.1±7.2;  G_3_: 21.3±5.5; G_4_: 17.3±3;  G_5_: 14.1±5.3; G_6_: 11.3±5.8;  G_7_: 39.0±5.4; G_8_: 5.1±8.0  G_1_/G_3_;G_6_;G_7_;G_8_^*^; G_7_/All^*^ | |
| Rodrigues et al, 2011[48] | G_1_: 37% phosphoric acid (10)  G_2_: ozone + 37% phosphoric acid (10)  G_3_: 37% phosphoric acid + ozone (10)  + adhesive | Third molars | 0.2% Thymol  (3 months) | Adhesive: Single Bond 2  Resin: TPH | G_1_: 29.7±3.7; G_2_: 15.0±4.6;  G_3_: 20.7±4.5  G_1_/G_2_;G_3_^*^; G_2_/G_3_^*^ | |
| Sharma et al, 2011[147] | G_1_: adhesive 1 (20)  G_2_: 2% CHX + adhesive 1 (20)  G_3_: Tubulicid Red + adhesive 1 (20)  G_4_: 1% CHX + adhesive 1 (20)  G_5_: Ora-5 + adhesive 1 (20)  G_6_: 34% phosphoric acid + adhesive 2 (20)  G_7_: 2% CHX + 34% phosphoric acid + adhesive 2 (20)  G_8_: Tubulicid Red + 34% phosphoric acid + adhesive 2 (20)  G_9_: 1% CHX +34% phosphoric acid + adhesive 2 (20)  G_10_: Ora-5 + 34% phosphoric acid + adhesive 2 (20)  + resin | Lower molars | 2.6% NaOCl | Tubulicid Red  Ora-5 (McHenry Labs, USA)  Adhesive: 1 - Clearfil SE Bond; 2 - Prime&Bond NT  Resin: Clearfil AP-X | G_1_: 20.99±4.94; G_2_: 16.89±2.47;  G_3_: 15.67±2.41; G_4_: 20.87±4.59;  G_5_: 15.44±2.89; G_6_: 21.08±3.94;  G_7_: 21.87±2.16; G_8_: 21.59±2.73;  G_9_: 21.69±3.60; G_10_: 21.62±2.36    G_1_/G_2;_G_3;_G_5_^*^; G_2_/G_4_;G_6_;G_7_;G_8_;G_9_;G_10_^*^; G_3_/G_4_;G_6_;G_7_;G_8_;G_9_;G_10_^*^; G_4_/G_5_^*^; G_5_/G_6_;G_7_;G_8_;G_9_;G_10_^*^ | |
| Stanislawczuk et al, 2011[91] | G_1_: phosphoric acid + adhesive 1 (7)  G_2_: phosphoric acid + 2% CHX + adhesive 1 (7) G_3_: phosphoric acid + adhesive 1 (2 years) (7) G_4_: phosphoric acid + 2% CHX + adhesive 1 (2 years) (7) G_5_: phosphoric acid + adhesive 2 (7)  G_6_: phosphoric acid + 2% CHX + adhesive 2 (7) G_7_: phosphoric acid + adhesive 2 (2 years) (7) G_8_: phosphoric acid + 2% CHX + adhesive 2 (2 years) (7)  + resin | Third molars | 0.5% Chloramine | Adhesive: 1 - Prime&Bond NT; 2 - Single Bond 2  Resin: Opallis | G_1_: 29.0±4.5; G_2_: 32.8±4.2;  G_3_: 13.5±6.3; G_4_: 26.5±4.6;  G_5_: 32.3±4.1; G_6_: 32.2±5.2;  G_7_: 17.2±5.9; G_8_: 26.1±5.4  G_1_/G_3_^*^; G_2_/G_4_^*^; G_3_/G_4_^*^; G_5_/G_7_^*^; G_6_/G_7_^*^; G_7_/G_8_^*^ | |
| Stanislawczuk et al, 2011[153] | G_1_: 34% phosphoric acid + adhesive 1 (5) G_2_: 34% phosphoric acid + 2% minocycline + adhesive 1 (5) G_3_: 34% phosphoric acid + 2% doxycycline + adhesive 1 (5) G_4_: 34% phosphoric acid + 2% CHX + adhesive 1 (5) G_5_: 35% phosphoric acid + adhesive 2 (5) G_6_: 35% phosphoric acid + 2% minocycline + adhesive 2 (5) G_7_: 35% phosphoric acid + 2% doxycycline + adhesive 2 (5) G_8_: 35% phosphoric acid + 2% CHX + adhesive 2 (5)  + resin | Third molars | N/A | Adhesive: 1 - Prime&Bond NT; 2 - Single Bond 2  Resin: Opallis | G_1_: 35.7±4.5; G_2_: 40.2±12.1;  G_3_: 30.3±8.2; G_4_: 38.1±8.3;  G_5_: 42.1±8.3; G_6_: 36.9±16.4;  G_7_: 40.2±13.2; G_8_: 42.8±10.4  G_2_/G_3_^*^; G_3_/G_4_^*^ | |
| Aguilera et al, 2012[34] | G_1_: none (deep dentin) (12) G_2_: none (superficial dentin) (12) G_3_: phosphoric acid + 5% NaOCl (deep dentin) (12) G_4_: phosphoric acid + 5% NaOCl (superficial dentin) (12)  + adhesive + resin + TC | Third molars | 0.5% Chloramine  (4ºC,  ≤ 1 month) | Adhesive: Prime & Bond NT  Resin: Tetric Ceram | G_1_: 8.46±4.2; G_2_: 12.1±4.3;  G_3_: 8.15±6.2; G_4_: 9.19±5.4 | |
| Castro et al, 2012[51] | G_1_: 35% phosphoric acid + adhesive (3)  G_2_: 35% phosphoric acid + adhesive + laser (0.75W) (3)  G_3_: 35% phosphoric acid + adhesive + laser (1W) (3)  G_4_: adhesive (3)  G_5_: adhesive + laser (0.75W) (3)  G_6_: adhesive + laser (1W) (3)  + resin | Third molars | Saline + 0.2% thymol | Laser Nd:YAG (Twin.Light, Fotona, Slovenia)  Adhesive: Single Bond  Resin: Filtek Z250 | G_1_: 50.0±12.8; G_2_: 43.8±10.3;  G_3_: 50.0±13.6; G_4_: 11.9±22.3;  G_5_: 30.1±12.1; G_6_: 32.8±10.9  G_1_/G_4_;G_5_;G_6_^*^; G_2_/G_4_;G_5_;G_6_^*^; G_3_/G_4_;G_5_;G_6_^*^; G_4_/G_5_;G_6_^*^ | |
| Dalkilic et al, 2012[143] | G_1_: ozone (5) G_2_: laser (5) G_3_: 2% CHX (5)  G_4_: none (5)  + adhesive + resin | Third molars | Saline | Laser Nd:YAG (Pulsmaster 600 IQ, American Dental Technologies, USA) Adhesive: Clearfil SE Bond  Resin: Clearfil Majesty | G_1_: 12.09±5.49; G_2_: 16.64±4.98;  G_3_: 19.06±6.19; G_4_: 17.30±7.88  G_1_/All^*^ | |
| Firat et al, 2012[94] | G_1_: 36% phosphoric acid (6)  G_2_: laser (50 µs) (6)  G_3_: 36% phosphoric acid + laser (50 µs) (6)  G_4_: laser (100 µs) (6)  G_5_: 36% phosphoric acid + laser (100 µs) (6)  G_6_: laser (150 µs) (6)  G_7_: 36% phosphoric acid + laser (150 µs) (6)  + adhesive + resin | Third molars | 0.5% Chloramine  (1 month) | Laser Er:YAG (Fidelis III, Fotona, Slovenia)  Adhesive: XP Bond  Resin: CeramX Duo (Dentsply, Germany) | G_1_: 31.85±10.34; G_2_: 23.39±7.79;  G_3_: 31.45±14.6; G_4_: 18.54±7.7;  G_5_: 29.25±8.3; G_6_: 16.16±8.32;  G_7_: 37.28±9.12  G_1_/G_2_;G_4_;G_6_^*^; G_2_/G_7_^*^; G_3_/G_4_;G_6_^*^; G_4_/G_5_;G_7_^*^; G_5_/G_6_;G_7_^*^; G_6_/G_7_^*^ | |
| Garcia et al, 2012[93] | G_1_: ozone + 35% phosphoric acid + adhesive 1 + resin 1 (5)  G_2_: ozone + 35% phosphoric acid + adhesive 2 + resin 2 (5)  G_3_: ozonated water + 35% phosphoric acid + adhesive 1 + resin 1 (5)  G_4_: ozonated water + 35% phosphoric acid + adhesive 2 + resin 2 (5)  G_5_: water + 35% phosphoric acid + adhesive 1 + resin 1 (5)  G_6_: water + 35% phosphoric acid + adhesive 2 + resin 2 (5) | Third molars | 0.5% Chloramine + distilled water  (6 months) | Adhesive: 1 - Single Bond 2; 2 - XP Bond  Resin: 1 – Filtek Z350; 2 - Esthet X | G_1_: 31.7±4.8; G_2_: 39.2±6.5;  G_3_: 30.1±1.9; G_4_: 39.4±6.8;  G_5_: 31.1±5.2; G_6_: 41.9±6.2 | |
| Gerhardt-Szep et al, 2012[52] | G_1_: laser (80 µm, 128 s) + adhesive (12)  G_2_: laser (80 µm, 128 s) + adhesive (bond) (10)  G_3_: laser (160 µm, 65 s) + adhesive (bond) (11)  G_4_: none (12)  + adhesive + resin (simulated pulp pressure) | Molars | 0.1% Thymol  + distilled water  (6 months) | Laser femtosecond (Zentrum Hannover, Germany)  Adhesive: Clearfil SE Bond  Resin: XRV Herculite (Kerr, Germany) | G_1_: 7.13±1.40; G_2_: 5.95±1.56;  G_3_: 8.37±1.21; G_4_: 9.75±1.22  G_1_/G_4_^*^; G_2_/G_3_;G_4_^*^ | |
| Lenzi et al, 2012[27] | 35% phosphoric acid +  G_1_: water (5)  G_2_: water (artificial caries) (5) G_3_: 2% CHX (5)  G_4_: 2% CHX (artificial caries) (5)  + adhesive + resin | Third molars | 0.5% Chloramine (4ºC, 30 days) | Adhesive: Single Bond 2 Resin: Filtek Z250 | G_1_: 41.7±2.7; G_2_: 29.1±6.0;  G_3_: 43.2±4.7; G_4_: 36.4±1.3  G_1_/G_2_;G_4_^*^; G_2_/G_3_^*^; G_3_/G_4_^*^ | |
| Sacramento et al, 2012[135] | G_1_: adhesive 1 (24h) (5)  G_2_: adhesive 2 (24h) (5)  G_3_: 2% CHX + adhesive 1 (24h) (5)  G_4_: 2% CHX + adhesive 2 (24h) (5)  G_5_: adhesive 1 (6 months) (5)  G_6_: adhesive 2 (6 months) (5)  G_7_: 2% CHX + adhesive 1 (6 months) (5)  G_8_: 2% CHX + adhesive 2 (6 months) (5)  G_9_: adhesive 1 (12 months) (5)  G_10_: adhesive 2 (12 months) (5)  G_11_: 2% CHX + adhesive 1 (12 months) (5)  G_12_: 2% CHX + adhesive 2 (12 months) (5)  + resin | Third molars | 0.9% NaCl + 0.02 sodium azide  (2 months) | Adhesive: 1 - Clearfil Protect Bond (Kuraray, Japan)  Resin: Charisma (Heraeus Kulzer, Germany) | G_1_: 12.28±2.91; G_2_: 16.24±2.71;  G_3_: 12.39±2.37; G_4_: 14.60±3.65;  G_5_: 2.95±0.77; G_6_: 2.32±0.60;  G_7_: 2.88±1.30; G_8_: 3.09±0.92;  G_9_: 1.36±0.22; G_10_: 1.11±0.59;  G_11_: 1.76±0.35; G_12_: 2.34±0.76 | |
| Sano et al, 2012[114] | G_1_: laser + adhesive (5) G_2_: adhesive (5) G_3_: laser + bond (5)  + resin | Molars | Distilled water | Laser ArF excimer (EX5 Excimer, GAM Laser, USA);  Adhesive: Clearfil Mega Bond;  Resin: Clearfil AP-X | G_1_: 16.7±7.3; G_2_: 13.0±3.3;  G_3_: 11.6±4.4 | |
| Baraba et al, 2013[95] | G_1_: none (12)  G_2_: laser (50 µsec, 10Hz, 80mJ) (12)  G_3_: laser (100 µsec, 10Hz, 80mJ) (12)  G_4_: laser (300 µsec, 10Hz, 80mJ) (12)  + adhesive + resin | Molars | 1% Chloramine | Laser Er:YAG  Adhesive: G-bond (GC, Japan)  Resin: Gradia Direct (GC, Japan) | G_1_: 35.3±12.8; G_2_: 24.0±9.8;  G_3_: 29.1±9.8; G_4_: 32.9±10.7  G_1_/G_2_^*^; G_2_/G_4_^*^ | |
| Bridi et al, 2013[55] | G_1_: adhesive 1 (6)  G_2_: adhesive 2 (6)  G_3_: 2.5% TiF_4_ + adhesive 1 (6)  G_4_: 2.5% TiF_4_ + adhesive 2 (6)  + resin | Third molars | 0.1% Thymol | Adhesive: 1 - Clearfil SE Bond; 2 - Easy One (3M, USA)  Resin: Filtek Z350 | G_1_: 19.31±8.41; G_2_: 16.19±7.04;  G_3_: 27.22±10.74; G_4_: 11.11±2.35    G_4_/All^*^ | |
| Chiang et al, 2013[146] | G_1_: water (24h) (6)  G_2_: 5% gluma (24h) (6)  G_3_: water + TC (6)  G_4_: 5% gluma + TC (6)  + adhesive + resin | Molars | 0.02% Sodium azide | Adhesive: Scotchbond Multi-purpose  Resin: Filtek Z250  Gluma (Heraeus Kulzer, USA) | G_1_: 30.25±12.41; G_2_: 42.12±21.64;  G_3_: 16.95±6.75; G_4_: 34.43±14.02  G_1_/G_2_^*^; G_3_/G_4_^*^ | |
| Davari et al, 2013[144] | G_1_: 37.5% phosphoric acid (15)  G_2_: laser (15)  G_3_: laser + 37.5% phosphoric acid (15)  G_4_: 37.5% phosphoric acid + laser (15)  G_5_: none (15)  + adhesive + resin + TC | Upper premolars | Formaldehyde (2h) + saline (3 months) | Laser Er:YAG (Kavo Key Laser 3)  Adhesive: Optibond FL  Resin: Point 4 (Kerr, USA) | G_1_: 20.1±1.8; G_2_: 14.1±3.4;  G_3_: 15.6±3.5; G_4_: 21.5±5.1;  G_5_: 8.1±2.1  G_1_/G_2_;G_3_;G_5_^*^; G_2_/G_4_;G_5_^*^; G_3_/G_4_;G_5_^*^; G_4_/G_5_^*^ | |
| Jiang et al, 2013[116] | G_1_: 37% phosphoric acid + adhesive 1 (23)  G_2_: adhesive 2 (23)  G_3_: adhesive 3 (20)  G_4_: adhesive 4 (21)  G_5_: adhesive 5 (22)  G_6_: laser + 37% phosphoric acid + adhesive 1 (23)  G_7_: laser + adhesive 2 (23)  G_8_: laser + adhesive 3 (20)  G_9_: laser + adhesive 4 (21)  G_10_: laser + adhesive 5 (22)  + resin | Posterior teeth | Distilled water | Laser Er:YAG (Smart-2940D; DEKA, Italy)  Adhesive: 1 - Prime & Bond NT; 2 - G-Bond plus (GC, Japan); 3 - XENO V (Dentsply, USA); 4 - iBond; 5 - Easy One  Resin: Filtek Z250 | G_1_: 10.67±5.78; G_2_: 5.02±1.87;  G_3_: 9.53±5.70; G_4_: 9.39±3.37;  G_5_: 12.75±4.29; G_6_: 14.18±6.20;  G_7_: 5.05±2.62; G_8_: 9.27±4.92;  G_9_: 6.90±2.67; G_10_: 11.90±5.65  G_1_/G_2_^*^; G_2_/G_4_;G_5_^*^; G_6_/G_7_;G_9_^*^; G_7_/G_8_;G_10_^*^; G_9_/G_10_^*^ | |
| Kasraei et al, 2013[118] | G_1_: adhesive 1 (9) G_2_: 2.5% NaOCl + adhesive 1 (9) G_3_: 0.5M EDTA + adhesive 1 (9) G_4_: adhesive 2 (9) G_5_: 2.5% NaOCl + adhesive 2 (9) G_6_: 0.5M EDTA + adhesive 2 (9)  + resin + TC | Premolars | Distilled water | Adhesive: 1 – iBond; 2 - Clearfil S3 Bond Resin: Filtek Z100 | G_1_: 19.6±4.6; G_2_: 32.6±7.7;  G_3_: 25.9±6.3; G_4_: 30.4±11.8;  G_5_: 31.6±7.7; G_6_: 27.5±10.8  G_1_/G_2_;G_4;_G_5_^*^ |  |
| de Oliveira et al, 2013[54] | G_1_: phosphoric acid + adhesive 1 (16) G_2_: laser (120 mJ) + phosphoric acid + adhesive 1 (16) G_3_: laser (140 mJ) + phosphoric acid + adhesive 1 (16)  G_4_: laser (180 mJ) + phosphoric acid + adhesive 1 (16) G_5_: laser (200 mJ) + phosphoric acid + adhesive 1 (16) G_6_: adhesive 2 + resin (16) G_7_: laser (120 mJ) + adhesive 2 (16) G_8_: laser (140 mJ) + adhesive 2 (16) G_9_: laser (180 mJ) + adhesive 2 (16) G_10_: laser (200 mJ) + adhesive 2 (16) G_11_: adhesive 3 (16) G_12_: laser (120 mJ) + adhesive 3 (16) G_13_: laser (140 mJ) + adhesive 3 (16) G_14_: laser (180 mJ) + adhesive 3 (16) G_15_: laser (200 mJ) + adhesive 3 (16)  + resin | Third molars | Thymol  (5°C,  ≤ 3 months) | Laser Er:YAG (Kavo Key Laser 3);  Adhesive: 1 - Single Bond Plus; 2 - Clearfil Protect Bond; 3 - Clearfil Tri-S Bond;  Resin: Clearfil AP-X | G_1_: 21.6±2.8; G_2_: 15.1±3.2;  G_3_: 12.0±2.2; G_4_: 13.8±2.4;  G_5_: 13.4±2.6; G_6_: 22.7±2.5;  G_7_: 23.7±6.1; G_8_: 24.0±3.1;  G_9_: 22.2±2.7; G_10_: 25.0±5.9;  G_11_: 21.2±2.5; G_12_: 17.6±7.6;  G_13_: 22.4±6.4;G_14_: 21.5±4.4;  G_15_: 24.3±4.9  G_1_/G_2_;G_3_;G_4_;G_5_^*^; G_2_/G_7_^*^; G_3_/G_8_;G_13_^*^; G_4_/G_9_;G_14_;G_15_^*^; G_5_/G_10_^*^; G_12_/G_15_^*^ | |
| Reddy et al, 2013[154] | G_1_: adhesive 1 (10)  G_2_: 2% CHX adhesive 1 (10)  G_3_: 2% NaOCl adhesive 1 (10)  G_4_: 3% H_2_O_2_ + adhesive 1 (10)  G_5_: adhesive 2 (10)  G_6_: 2% CHX + adhesive 2 (10)  G_7_: 2% NaOCl + adhesive 2 (10)  G_8_: 3% H_2_O_2_ + adhesive 2 (10)  + resin | Posterior teeth | N/A | Adhesive: 1 - Adper SE Plus (3M, USA); 2 - Easy One  Resin: Filtek Z250 | G_1_: 19.89±1.36; G_2_: 15.87±1.27;  G_3_: 13.82±1.23; G_4_: 13.28±0.63;  G_5_: 15.76±1.46; G_6_: 13.42±1.08;  G_7_: 13.49±2.02; G_8_: 13.13±1.83  G_1_/G_3_;G_5_;G_7_^*^; G_2_/G_4_;G_6_^*^; G_2_/G_8_^*^; G_3_/G_7_^*^ | |
| Ribeiro et al, 2013[117] | G_1_: 37% phosphoric acid + adhesive (14)  G_2_: 37% phosphoric acid + adhesive + laser 1 (14)  G_3_: laser 2 + adhesive (14)  + resin | Molars | Distilled water (29 days) | Laser: 1 - Nd:YAG (American Dental Technologies, USA); 2 - Er:YAG (Kavo Key Laser)  Adhesive: Single Bond  Resin: Filtek Z100 | G_1_: 17.05±4.15; G_2_: 16.90±3.36;  G_3_: 12.12±3.85  G_1_/G_3_^*^; G_2_/G_3_^*^ | |
| Santiago et al, 2013[56] | 35% phosphoric acid +  G_1_: none (3) G_2_: 0.02% EGCG (3)  G_3_: 0.1% EGCG (3)  G_4_: 0.5% EGCG (3)  G_5_: 2% CHX (3)  G_6_: none (6 months) (3) G_7_: 0.02% EGCG (6 months) (3)  G_8_: 0.1% EGCG (6 months) (3)  G_9_: 0.5% EGCG (6 months) (3)  G_10_: 2% CHX (6 months) (3)  + adhesive + resin | Third molars | 0.01% Thymol (≤1 month) | Adhesive: Single Bond 2  Resin: Filtek Z250 | G_1_: 34.17±7.75; G_2_: 31.39±7.82;  G_3_: 34.74±9.14; G_4_: 27.11±7.78;  G_5_: 34.68±7.30; G_6_: 27.67±6.98;  G_7_: 31.75±10.58; G_8_: 35.99±10.91;  G_9_: 31.18±9.29; G_10_: 31.62±5.78  G_1_/G_4_;G_6_^*^; G_3_/G_4_^*^; G_4_/G_5_^*^; G_6_/G_8_^*^ | |
| Saraceni et al, 2013[115] | G_1_: 35% phosphoric acid + adhesive 1 (10)  G_2_: 37% phosphoric acid + adhesive 2 (10)  G_3_: 35% phosphoric acid + 10% NaOCl + adhesive 1 (10)  G_4_: 37% phosphoric acid + 10% NaOCl + adhesive 2 (10)  G_5_: laser + adhesive 1 (10)  G_6_: laser + adhesive 2 (10)  + resin | Third molars | Distilled water (6 months) | Laser Er:YAG (LELO, Brazil)  Adhesive: 1 - Single Bond 2; 2 - Prime & Bond 2.1  Resin: Filtek Z250 | G_1_: 19.9±5.7; G_2_: 20.6±4.2;  G_3_: 13.0±4.4; G_4_: 23.1±5.2;  G_5_: 11.4±2.9; G_6_: 17.9±4.0  G_1_/G_2_;G_5_^*^; G_2_/G_4_;G_6_^*^; G_3_/G_5_^*^; G_4_/G_6_^*^ | |
| Verma et al, 2013[53] | phosphoric acid +  G_1_: adhesive 1 (24h) (10)  G_2_: 2% CHX + adhesive 1 (24h) (10)  G_3_: 30% proanthocyanidin + adhesive 1 (24h) (10)  G_4_: adhesive 2 (24h) (10)  G_5_: 2% CHX + adhesive 2 (24h) (10)  G_6_: 30% proanthocyanidin + adhesive 2 (24h) (10)  G_7_: adhesive 1 (6 months) (10)  G_8_: 2% CHX + adhesive 1 (6 months) (10)  G_9_: 30% proanthocyanidin + adhesive 1 (6 months) (10)  G_10_: adhesive 2 (6 months) (10)  G_11_: 2% CHX + adhesive 2 (6 months) (10)  G_12_: 30% proanthocyanidin + adhesive 1 (6 months) (10)  + resin | Molars | 0.1% Thymol | Adhesive: 1 - Solobond M (VOCO, Germany); 2 - Tetric N Bond (Ivoclar Liechtenstein)  Resin: Filtek Z350 | G_1_: 7.71±2.58; G_2_: 8.57±2.45;  G_3_: 8.74±1.53; G_4_: 7.77±2.63;  G_5_: 9.11±3.17; G_6_: 8.44±2.51;  G_7_: 4.23±1.46; G_8_: 9.31±2.30;  G_9_: 8.15±2.16; G_10_: 4.96±1.88;  G_11_: 9.03±2.81; G_12_: 8.38±2.16  G_1_/G_7_;G_10_^*^; G_2_/G_7_;G_10_^*^; G_3_/G_7_;G_10_^*^; G_4_/G_7_;G_10_^*^; G_5_/G_7_;G_10_^*^; G_6_/G_7_;G_10_^*^; G_7_/G_8_;G_9_;G_11_;G_12_^*^; G_8_/G_10_^*^; G_9_/G_10_^*^; G_10_/G_11_;G_12_^*^ | |
| Castro et al, 2014[60] | G_1_: adhesive (3) G_2_: adhesive + laser (0.75 W) (3) G_3_: adhesive + laser (1W) (3) G_4_: bond (3) G_5_: bond + laser (0.75 W) (3) G_6_: bond + laser (1W) (3)  + resin | Third molars | 0.2% Thymol (4°C) | Laser Nd:YAG (Twin-Light, Fotona, Slovenia); Adhesive: Clearfil SE Bond Resin: Filtek Z250 | G_1_: 46.2±15.8; G_2_: 35.3±10.4;  G_3_: 40.0±12.2; G_4_: 49.8±15.9;  G_5_: 55.7±15.6; G_6_: 56.1±12.8  G_2_/G_3_;G_4_;G_5_;G_6_^*^; G_3_/G_5_;G_6_^*^ |  |
| Elkassas et al, 2014[132] | G_1_: adhesive 1 (10)  G_2_: 35% phosphoric acid + adhesive 2 (10)  G_3_: 5.25% NaOCl + adhesive 1 (10)  G_4_: 5.25% NaOCl + 35% phosphoric acid + adhesive 2 (10)  G_5_: Tubulicid Red + adhesive 1 (10)  G_6_: Tubulicid Red + 35% phosphoric acid + adhesive 2 (10)  G_7_: 2% CHX + adhesive 1 (10)  G_8_: 2% CHX + 35% phosphoric acid + adhesive 2 (10)  G_9_: Biopure + adhesive 1 (10)  G_10_: Biopure + 35% phosphoric acid + adhesive 2 (10)  + resin | Molars | Saline  (2 weeks) | Tubulicid Red  Biopure (Dentsply, USA)  Adhesive: 1 - Clearfil SE Bond; 2 - Single Bond 2  Resin: Clearfil AP-X | G_1_: 14.58±4.7; G_2_: 13±4.5;  G_3_: 20.46±5.9; G_4_: 6.46±2.1;  G_5_: 16.98±3.5; G_6_: 6.14±3.5;  G_7_: 16.03±5.6; G_8_: 9.3±4.6;  G_9_: 16.76±5.2; G_10_: 10.65±2.8  G_1_/G_3_;G_4_;G_5_;G_6_;G_7_;G_8_;G_9_;G_10_^*^;  G_2_/G_3_;G_4_;G_5_;G_6_;G_7_;G_8_;G_9_;G_10_^*^;  G_3_/All^*^; G_4_/G_5_;G_7_;G_8_;G_9_;G_10_^*^;  G_4_/G_5_;G_7_;G_8_;G_10_^*^; G_5_/G_6_;G_8_;G_9_;G_10_^*^; G_6_/G_7_;G_8_;G_9_;G_10_^*^; G_7_/G_8_;G_9_;G_10_;G_8_/G_9_^*^; G_9_/G^10*^ |  |
| Galafassi et al, 2014[62] | 35% phosphoric acid +  G_1_: deionized water (24h) (10)  G_2_: 2% CHX (24h) (10)  G_3_: deionized water (6 months) (10)  G_4_: 2% CHX (6 months) (10)  G_5_: deionized water (12 months) (10)  G_6_: 2% CHX (12 months) (10)  + adhesive + resin + TC | Third molars | 0.2% Thymol (48h) | Adhesive: Single Bond 2  Resin: Filtek Z350 | G_1_:27.89±10.11; G_2_: 29.30±5.66;  G_3_: 21.16±7.43; G_4_: 21.82±5.97;  G_5_: 23.85±7.43; G_6_: 21.29±13.35 |  |
| Manso et al, 2014[59] | 32% phosphoric acid +  G_1_: ethanol + adhesive 1 (6) G_2_: ethanol + adhesive 2 (6) G_3_: distilled water + adhesive 1 (6) G_4_: distilled water + adhesive 2 (6)  + resin | Third molars | 0.1% Thymol (4°C,  ≤6 months) | Adhesive: 1 - All Bond 3 (Bisco, USA); 2 - Excite Resin: Aelite All Purpose Body (Bisco, USA) | 24h  G_1_: 59.41±3.6; G_2_: 49.67±5.4;  G_3_: 51.07±3.6; G_4_: 49.51±5.4  6 months  G_1_: 56.41±3.6; G_2_: 44.56±5.4;  G_3_: 57.13±3.6; G_4_: 42.10±5.4  G_1_/G  15 months  G_1_: 44.41±4.4; G_2_: 42.48±5.4; G_3_: 47.29±4.4; G_4_: 45.51±6.6 |  |
| Shirani et al, 2014[58] | G_1_: adhesive 1 (10)  G_2_: laser (0.5 mm) + adhesive 1 (10)  G_3_: laser (2 mm) + adhesive 1 (10)  G_4_: laser (4 mm) + adhesive 1 (10)  G_5_: laser (11 mm) + adhesive 1 (10)  G_6_: adhesive 2 (10)  G_7_: laser (0.5 mm) + adhesive 2 (10)  G_8_: laser (2 mm) + adhesive 2 (10)  G_9_: laser (4 mm) + adhesive 2 (10)  G_10_: laser (11 mm) + adhesive 2 (10)  + resin | Third molars | 0.2% Thymol  (3 months) | Laser Er:YAG (Fidelis Plus, Fotona, Slovenia)  Adhesive: 1 - Single Bond; 2 - Clearfil SE Bond  Resin: Filtek Z100 | G_1_: 14.43±4.54; G_2_: 6.58±2.5;  G_3_: 8.2±3.8; G_4_: 12.31±4.9;  G_5_: 12.73±3.2; G_6_: 18.04±2.55;  G_7_: 10.54±3.9; G_8_: 12.1±3.8;  G_9_: 12.17±6.5; G_10_: 15.28±5.1    G_1_/G_2_;G_3_^*^; G_2_/G_5_^*^ |  |
| Simões et al, 2014[61] | 32% phosphoric acid +  G_1_: none (24h) (9)  G_2_: 2% CHX (24h) (9)  G_3_: 100% ethanol (24h) (9)  G_4_: none (6 months) (9)  G_5_: CHX (6 months) (9)  G_6_: 100% ethanol (6 months) (9)  + adhesive + resin | Third molars | 0.1% Thymol | Adhesive: All Bond 3  Resin: Filtek Z100 | G_1_: 28.77±11.02; G_2_: 24.43±8.01;  G_3_: 20.86±7.09; G_4_: 19.37±5.74;  G_5_: 17.47±6.62; G_6_: 19.28±9.68 |  |
| Zheng et al, 2014[57] | G_1_: 37% phosphoric acid + adhesive 1 (5) G_2_: 37% phosphoric acid + adhesive 1 (9 months) (3) G_3_: 2% CHX + 37% phosphoric acid + adhesive 1 (9 months) (3) G_4_: 0.05% green tea extract + 37% phosphoric acid + adhesive 1 (9 months) (3) G_5_: FeSO_4_ + 37% phosphoric acid + adhesive 1 (9 months) (3)  G_6_: adhesive 2 (5) G_7_: adhesive 2 (9 months) (3) G_8_: 2% CHX + adhesive 2 (9 months) (3) G_9_: 0.05% green tea extract + adhesive 2 (9 months) (3) G_10_: FeSO_4_ + adhesive 2 (9 months) (3)  + resin | Molars | 0.01% Thymol (≤ 1 month) | Adhesive: 1 - Optibond FL; 2 - Clearfil SE Bond Resin: CeramX Mono (Dentsply, USA) | G_1_: 32.1±14.8; G_2_: 20.3±13.6;  G_3_: 32.9±11.3; G_4_: 33.2±14.0;  G_5_: 25.3±10.5; G_6_: 28.3±12.4;  G_7_: 25.3±11.8; G_8_: 32.9±11.3;  G_9_: 26.1±14.2; G_10_: 25.3±10.5  G_1_/G_2_;G_5_;G_6_;G_7_;G_8_;G_9_;G_10_^*^; G_2_/G_3_;G_4_^*^; G_3_/G_5_;G_6_;G_7_;G_8_;G_9_;G_10_^*^; G_4_/G_5_;G_6_;G_7_;G_8_;G_9_;G_10_^*^ |  |
| Bueno et al, 2015[36] | G_1_: none (superficial dentin) (10) G_2_: 2% CHX (superficial dentin) (10) G_3_: none (6 months) (superficial dentin) (10) G_4_: 2% CHX (6 months) (superficial dentin) (10) G_5_: none (deep dentin) (10) G_6_: 2% CHX (deep dentin) (10) G_7_: none (6 months) (deep dentin) (10) G_8_: 2% CHX (6 months) (deep dentin) (10)  + adhesive + resin | Third molars | 0.1% Thymol | Adhesive: Clearfil SE Bond Resin: Filtek Z250 | G_1_: 24.8±11.0; G_2_: 30.4±7.0;  G_3_: 28.1±9.4; G_4_: 30.2±6.6;  G_5_: 24.2±7.2; G_6_: 28.0±8.4;  G_7_: 21.8±7.3; G_8_: 33.4±9.3 |  |
| Chauhan et al, 2015[64] | 37% phosphoric acid +  G_1_: none (10)  G_2_: 5% NaOCl (10)  + adhesive + resin | Premolars | 0.1% Thymol | Adhesive: Single Bond 2  Resin: Filtek Z250 | G_1_: 14.24±3.17; G_2_: 20.52±2.27 |  |
| Ebrahimi-Chaharom et al, 2015[158] | G_1_: adhesive 1 (15)  G_2_: 5.25% NaOCl + adhesive 1 (15)  G_3_: adhesive 2 (15)  G_4_: 5.25% NaOCl + adhesive 2 (15)  + resin | Premolars | 0.5% Chloramine + distilled water | Adhesive: 1 - Clearfil SE Bond; 2 - Easy One  Resin: Filtek Z100 | G_1_: 36.82±5.35; G_2_: 26.19±4.48;  G_3_: 32.81±7.79; G_4_: 23.64±4.35  G_1_/G_2_;G_4_^*^; G_2_/G_3_^*^; G_3_/G_4_^*^ |  |
| Ercan et al, 2015[133] | G_1_: none (15) G_2_: ozone (15)  G_3_: 2% CHX (15) G_4_: 5% boric acid (15)  + adhesive + resin | Molars | Saline (4ºC) | Adhesive: Clearfil S3 Bond Plus Resin: Clearfil AP-X | G_1_: 15.47±2.67; G_2_: 13.98±2.67;  G_3_: 12.81±3.01; G_4_: 12.63±2.45  G_1_/G_3_;G_4_^*^; G_2_/G_3_;G_4_^*^ |  |
| Heredia et al, 2015[120] | G_1_: 35% phosphoric acid + adhesive 1 (6)  G_2_: laser + adhesive 1 (6)  G_3_: adhesive 2 (6)  G_4_: laser + adhesive 2 (6)  + resin | Third molars | Distilled water (6 months) | Adhesive: 1 - Single Bond 2; 2 - Clearfil SE Bond  Laser Nd:YAG (Pulse Master 1000)  Resin: Filtek Z250 | G_1_: 20.68±7.86; G_2_: 15.12±6.88;  G_3_: 11.27±4.95; G_4_: 10.08±4.77  G_1_/G_2_^*^; G_1_/G_3_^*^; G_2_/G_3_^*^ |  |
| Maenosono et al, 2015[63] | G_1_: 37% phosphoric acid + adhesive 1 (10)  G_2_: adhesive 2 (10)  G_3_: 37% phosphoric acid + adhesive 1 + laser (10)  G_4_: adhesive 2 + laser (10)  + resin | Third molars | 0.1% Thymol | Laser Nd:YAG (Sirolaser, Sirona, Germany)  Adhesive: 1 – Single Bond 2; 2 – Easy One  Resin: Filtek Z250 | G_1_: 33.49±6.77; G_2_: 19.67±5.86;  G_3_: 43.69±8.15; G_4_: 29.87±6.98  G_1_/G_2;_G_3_^*^; G_2_/G_4_^*^; G_3_/G_4_^*^ |  |
| Montagner et al, 2015[35] | G_1_: adhesive 1 (oclusal, superficial) (5)  G_2_: adhesive 1 (oclusal, deep) (5)  G_3_: adhesive 1 (proximal) (5)  G_4_: 10% NaOCl + adhesive 1(oclusal, superficial) (5)  G_5_: 10% NaOCl + adhesive 1 (oclusal, deep) (5)  G_6_: 10% NaOCl + adhesive 1 (proximal) (5)  G_7_: adhesive 2 (oclusal, superficial) (5)  G_8_: adhesive 2 (oclusal, deep) (5)  G_9_: adhesive 2 (proximal) (5)  G_10_: 10% NaOCl + adhesive 2 (oclusal, superficial) (5)  G_11_: 10% NaOCl + adhesive 2 (oclusal, deep) (5)  G_12_: 10% NaOCl + adhesive 2 (proximal) (5)  G_13_: 35% phosphoric acid + adhesive 3 (oclusal, superficial) (5)  G_14_: 35% phosphoric acid + adhesive 3 (oclusal, deep) (5)  G_15_: 35% phosphoric acid + adhesive 3 (proximal) (5)  G_16_: 35% phosphoric acid + 10% NaOCl + adhesive 3 (oclusal, superficial) (5)  G_17_: 35% phosphoric acid + 10% NaOCl + adhesive 3 (oclusal, deep) (5)  G_18_: 35% phosphoric acid + 10% NaOCl + adhesive 3 (proximal) (5)  G_19_: adhesive 4 (oclusal, superficial) (5)  G_20_: adhesive 4 (oclusal, deep) (5)  G_21_: adhesive 4 (proximal) (5)  G_22_: 10% NaOCl + adhesive 4 (oclusal, superficial) (5)  G_23_: 10% NaOCl + adhesive 4 (oclusal, deep) (5)  G_24_: 10% NaOCl + adhesive 4 (proximal) (5)  + resin | Third molars | 0.5% Thymol | Adhesive: 1 - G-Bond; 2 - Clearfil SE Bond; 3 - Single Bond 2; 4 - Adper SE Plus  Resin: Filtek Z250 | G_1_: 6.3±0.9; G_2_: 7.6±2.3;  G_3_: 6.9±1.2; G_4_: 7.8±2.8;  G_5_: 9.1±1.1; G_6_: 11.2±5.0;  G_7_: 10.4±3.5; G_8_: 6.9±1.6;  G_9_: 13.2±3.5; G_10_: 9.4±2.4;  G_11_: 7.9±3.4; G_12_: 16.3±6.5;  G_13_: 11.5±2.4; G_14_: 7.7±2.8;  G_15_: 14.9±4.5; G_16_: 11.6±3.2;  G_17_: 4.2±1.8; G_18_: 15.5±3.5;  G_19_: 9.4±3.6; G_20_: 6.4±2.6;  G_21_: 13.4±5.5; G_22_: 10.4±1.6;  G_23_: 12.2±3.3; G_24_: 13.7±2.4  G_1_/G_7_;G_13_^*^; G_3_/G_15_^*^; G_5_/G_17_^*^; G_8_/G_9_;G_12_^*^; G_9_/G_11_^*^; G_11_/G_12_^*^; G_14_/G_15_;G_16_;G_18_^*^; G_15_/G_17_^*^; G_16_/G_17_^*^; G_17_/G_18_;G_23_^*^; G_20_/G_21_;G_22_;G_23_;G_24_^*^ |  |
| Ozsoy et al, 2015[26] | G_1_: 37.5% phosphoric acid (3) G_2_: 37.5% phosphoric acid + 99.5% ethanol (3) G_3_: ozone + 37.5% phosphoric acid (3) G_4_: 37.5% phosphoric acid + 2% CHX (3) G_5_: 37.5% phosphoric acid (infected dentin) (3) G_6_: 37.5% phosphoric acid + 99.5% ethanol (infected dentin) (3) G_7_: ozone + 37.5% phosphoric acid (infected dentin) (3) G_8_: 37.5% phosphoric acid + 2% CHX (infected dentin) (3)  + adhesive + resin | Molars | 0.5% Chloramine  (≤3 months) | Adhesive: Optibond FL Resin: Tetric Ceram | G_1_: 34.62±4.51; G_2_: 30.20±2.43;  G_3_: 21.56±2.42; G_4_: 30.39±2.85;  G_5_: 21.78±4.06; G_6_: 22.05±3.85;  G_7_: 16.98±3.97; G_8_: 23.05±4.92  G_1_/All^*^; G_2_/G_3_^*^; G_3_/G_4_^*^; G_4_/G_5_;G_6_;G_7_;G_8_^*^; G_5_/G_7_^*^; G_6_/G_7_^*^; G_7_/G_8_^*^ |  |
| Portillo et al, 2015[119] | G_1_: adhesive 1 (3)  G_2_: adhesive 2 (3)  G_3_: adhesive 3 (3)  G_4_: laser 1 + adhesive 1 (3)  G_5_: laser 1 + adhesive 2 (3)  G_6_: laser 1 + adhesive 3 (3)  G_7_: laser 2 + adhesive 1 (3)  G_8_: laser 2 + adhesive 2 (3)  G_9_: laser 2 + adhesive 3 (3)  + resin | Third molars | Distilled water (24h) | Adhesive: 1 - Scotchbond 1 XT; 2 - Clearfil SE Bond; 3 - Optibond All-in-One (Kerr, USA)  Resin: Filtek Z250  Laser: 1 - Er:YAG (Fotona, Slovenia); 2 - Ti:sapphire (Tsunami, USA) | G_1_: 28.2±8.0; G_2_: 43.3±10.9;  G_3_: 24.1±7.4; G_4_: 23.9±8.0;  G_5_: 34.9±10.4; G_6_: 23.9±5.9;  G_7_: 22.4±8.2; G_8_: 29.2±6.7;  G_9_: 21.0±7.5  G_1_/G_2_;G_3_;G_4_;G_7_^*^; G_2_/G_8_^*^; G_4_/G_5_^*^; G_5_/G_6_^*^; G_7_/G_8_^*^; G_8_/G_9_^*^ |  |
| da Silva et al, 2015[98] | 37% phosphoric acid +  G_1_: adhesive 1 (distilled water, 24h) (3)  G_2_: adhesive 1 (distilled water, 15 days) (3)  G_3_: adhesive 1 (mineral oil, 24h) (3)  G_4_: adhesive 1 (mineral oil, 15 days) (3)  G_5_: adhesive 1 (1% NaOCl, 24h) (3)  G_6_: adhesive 1 (1% NaOCl, 15 days) (3)  G_7_: 2% CHX + adhesive 1 (distilled water, 24h) (3)  G_8_: 2% CHX + adhesive 1 (distilled water, 15 days) (3)  G_9_: 2% CHX + adhesive 1 (mineral oil, 24h) (3)  G_10_: 2% CHX + adhesive 1 (mineral oil, 15 days) (3)  G_11_: 2% CHX + adhesive 1 (1% NaOCl, 24h) (3)  G_12_: 2% CHX + adhesive 1 (1% NaOCl, 15 days) (3)  G_13_: adhesive 2 (distilled water, 24h) (3)  G_14_: adhesive 2 (distilled water, 15 days) (3)  G_15_: adhesive 2 (mineral oil, 24h) (3)  G_16_: adhesive 2 (mineral oil, 15 days) (3)  G_17_: adhesive 2 (1% NaOCl, 24h) (3)  G_18_: adhesive 2 (1% NaOCl, 15 days) (3)  G_19_: 2% CHX + adhesive 2 (distilled water, 24h) (3)  G_20_: 2% CHX + adhesive 2 (distilled water, 15 days) (3)  G_21_: 2% CHX + adhesive 2 (mineral oil, 24h) (3)  G_22_: 2% CHX + adhesive 2 (mineral oil, 15 days) (3)  G_23_: 2% CHX + adhesive 2 (1% NaOCl, 24h) (3)  G_24_: 2% CHX + adhesive 2 (1% NaOCl, 15 days) (3)  + resin | Molars | 0.5% Chloramine + distilled water | Adhesive: 1 - Single Bond 2; 2 - Ambar  Resin: Filtek Z350 | G_1_: 11.4±3.6; G_2_: 6.3±2.5;  G_3_: 13.7±3.5; G_4_: 9.8±4.0;  G_5_: 9.9±4.2; G_6_: 4.2±3.9;  G_7_: 21.7±6.7; G_8_: 11.1±3.6;  G_9_: 14.8±2.9; G_10_: 18.0±11.3;  G_11_: 14.3±3.2; G_12_: 5.3±3.1;  G_13_: 12.5±7.6; G_14_: 7.7±3.6;  G_15_: 9.6±7.2; G_16_: 14.0±3.7;  G_17_: 14.2±3.7; G_18_: 8.6±4.5;  G_19_: 11.2±5.9; G_20_: 6.8±4.2;  G_21_: 12.7±5.7; G_22_: 10.2±2.8;  G_23_: 10.3±4.0; G_24_: 4.1±1.6  G_1_/G_6_;G_7_;G_24_^*^; G_2_/G_3_;G_7_;G_9_;G_10_;G_11_;G_16_;G_17_^*^; G_3_/G_6_;G_7_;G_12_;G_24_^*^; G_4_/G_7_;G_10_^*^; G_5_/G_7_;G_10_^*^; G_6_/G_7_;G_9_;G_10_;G_11_;G_13_;G_16_;G_17_;G_21_^*^; G_7_/G_8_;G_11_;G_12_;G_13_;G_14_;G_15_;G_16_;G_17_;G_18_;G_19_;G_20_;G_21_;G_22_;G_23_;G_24_^*^; G_9_/G_12_;G_20_;G_24_^*^; G_10_/G_12_;G_14_;G_15_;G_18_;G_20_;G_24_^*^; G_11_/G_12_;G_24_^*^; G_12_/G_16_;G_17_^*^; G_13_/G_24_^*^; G_16_/G_24_^*^; G_17_/G_24_^*^ |  |
| Ustunkol et al, 2015[96] | G_1_: laser (15)  G_2_: 35% phosphoric acid (15)  G_3_: none (15)  + adhesive + resin | Third molars | 0.5% Chloramine  (4ºC, 1 month) | Laser Er,Cr:YSGG (Waterlase MD, Biolase, USA)  Adhesive: Silorane System Adhesive (3M, USA)  Resin: Filtek Silorane Restorative (3M, USA) | G_1_: 20.98±6.68; G_2_: 34.10±12.33;  G_3_: 13.92±8.06  G_1_/G_2_^*^; G_2_/G_3_^*^ |  |
| Zhou et al, 2015[97] | G_1_: 5.25% NaOCl + adhesive 1 (7)  G_2_: 5.25% NaOCl + adhesive 1 (TC) (7)  G_3_: 5.25% NaOCl + adhesive 2 (7)  G_4_: 5.25% NaOCl + adhesive 2 (TC) (7)  G_5_: 5.25% NaOCl + adhesive 3 (7)  G_6_: 5.25% NaOCl + adhesive 3 (TC) (7)  G_7_: adhesive 1 (7)  G_8_: adhesive 1 (TC) (7)  G_9_: adhesive 2 (7)  G_10_: adhesive 2 (TC) (7)  G_11_: adhesive 3 (7)  G_12_: adhesive 3 (TC) (7)  + resin | Third molars | 1% Chloramine  (1 month) | Adhesive: 1 - Clearfil S3 Bond; 2 - G-Bond; 3 - Xeno V  Resin: Charisma | G_1_: 26,72±3.3; G_2_: 20.02±2.8;  G_3_: 34.63±3.9; G_4_: 23.50±3.5;  G_5_: 21.06±2.8; G_6_: 13.75±2.1;  G_7_: 28.41±4.4; G_8_: 21.54±3.4;  G_9_: 37.08±3.5; G_10_: 29.00±4.4;  G_11_: 27.38±2.8; G_12_: 20.56±2.4  G_1_/G_2_;G_3_;G_4_;G_5_;G_6_;G_9_;G_10_;G_11_;G_12_^*^; G_2_/G_3_;G_4_;G_5_;G_6_;G_7_;G_9_;G_10_;G_11_;G_12_^*^; G_3_/G_4_;G_5_;G_6_;G_7_;G_8_;G_11_;G_12_^*^; G_4_/G_5_;G_6_;G_7_;G_8_;G_9_;G_10_;G_11_;G_12_^*^; G_5_/G_6_;G_7_;G_8_;G_9_;G_10_;G_11_^*^; G_6_/G_7_;G_8_;G_9_;G_10_;G_11_;G_12_^*^; G_7_/G_8_;G_9_;G_10_;G_11_;G_12_^*^; G_8_/G_9_;G_10_;G_11_;G_12_^*^; G_9_/G_10_;G_11_;G_12_^*^; G_10_/G_11_;G_12_^*^; G_11_/G_12_^*^ |  |
| Alaghehmand et al, 2016[37] | G_1_: 37% phosphoric acid (superficial dentin) (10)  G_2_: 37% phosphoric acid (deep dentin) (10)  G_3_: laser (superficial dentin) (10)  G_4_: laser (deep dentin) (10)  G_5_: laser + 37% phosphoric acid (superficial dentin) (10)  G_6_: laser + 37% phosphoric acid (deep dentin) (10)  + adhesive + resin + TC | Third molars | 0.9% NaCl  (6 months) | Laser Er:YAG (Dr Smile, Italy)  Adhesive: Single Bond 2  Resin: Filtek Z250 | G_1_: 5.33±2.15; G_2_: 4.70±1.21;  G_3_: 2.95±0.66; G_4_: 2.74±0.64;  G_5_: 4.97±1.34; G_6_: 1.92±0.50  G_1_/G_3_;G_4_;G_6_^*^; G_2_/G_3_;G_4_;G_6_^*^; G_3_/G_5_;G_6_^*^; G_4_/G_5_^*^; G_5_/G_6_^*^ |  |
| Balloni et al, 2016[68] | G_1_: DTUS + 2% CHX + adhesive + resin (24h) (6)  G_2_: DT + 2% CHX + adhesive + resin (24h) (6)  G_3_: DTUS + 2% CHX + adhesive + resin (6 months) (6)  G_4_: DT + 2% CHX + adhesive + resin (6 months) (6)  G_5_: DTUS + adhesive + resin (24h) (6)  G_6_: DT + adhesive + resin (24h) (6)  G_7_: DTUS + adhesive + resin (6 months) (6)  G_8_: DT + adhesive + resin (6 months) (6) | Third molars | 1% Thymol  (6 months) + water (24h) | DT: Diamond tip 1092 (KG Sorensen, Brazil)  DTUS: Ultrasound diamond tip CR1 (CVDentUS, Brazil)  Adhesive: Clearfil SE Bond  Resin: Charisma | G_1_: 19.87±11.84; G_2_: 19.24±11.89;  G_3_: 14.35±8.14; G_4_: 11.97±9.95;  G_5_: 20.55±13.15; G_6_: 12.67±7.43;  G_7_: 16.86±10.54; G_8_: 10.22±5.00  G_1_/G_2_^*^; G_3_/G_4_^*^; G_5_/G_6_^*^; G_7_/G_8_^*^ |  |
| Cersosimo et al, 2016[29] | G_1_: laser (20)  G_2_: none (20)  G_3_: none (artificial caries) (20)  G_4_: laser (artificial caries) (20)  + adhesive + resin | Third molars | 0.5% Chloramine  (3 months) | Laser Er:YAG (Fidelis III, Slovenia)  Adhesive: Clearfil SE Bond  Resin: Filtek Z350 | G_1_: 12.77±5.09; G_2_: 9.76±3.39;  G_3_: 5.12±1.72; G_4_: 7.62±3.39  G_1_/All^*^; G_2_/All^*^; G_3_/All^*^; G_4_/All^*^ |  |
| Cha et al, 2016[66] | G_1_: none (15) G_2_: 2% CHX + wash (15) G_3_: 6% NaOCl + wash (15) G_4_: 0.01% urushiol + wash (15) G_5_: 2% CHX (15) G_6_: 6% NaOCl (15) G_7_: 0.01% urushiol (15)  + adhesive + resin | Molars | Thymol  (4ºC,  ≤ 2 weeks) | Adhesive: Scotchbond Universal Resin: Filtek Z350 | G_1_: 16.74±5.14; G_2_: 15.07±4.86;  G_3_: 13.45±3.50; G_4_: 14.84±4.12;  G_5_: 14.10±5.56; G_6_: 10.96±4.57;  G_7_: 12.06±4.47  G_1_/G_6_^*^ |  |
| Gan et al, 2016[22] | G_1_: 35% phosphoric acid (50)  G_2_: 35% phosphoric acid (TC) (50)  G_3_: 35% phosphoric acid (10% NaOCl) (50)  G_4_: laser + 35% phosphoric acid (50)  G_5_: laser + 35% phosphoric acid (TC) (50)  G_6_: laser + 35% phosphoric acid (10% NaOCl) (50)  + adhesive + resin | Molars | 0.9% NaCl + 0.002% sodium azide (4ºC, 1 month) | Laser Nd:YAG (Miracle Laser-3100, Miracle Laser Technologies, China)  Adhesive: Single Bond 2  Resin: Charisma | G_1_: 20.8±6.4; G_2_: 15.1±5.8;  G_3_: 16.1±4.7; G_4_: 21.0±6.4;  G_5_: 20.6±7.1; G_6_: 20.8±5.1  G_1_/G_2_;G_3_^*^; G_2_/G_4_;G_5_;G_6_^*^; G_3_/G_4_;G_5_;G_6_^*^ |  |
| Gerhardt et al, 2016[67] | G_1_: 2% CHX (24h) (8) G_2_: 2% EGCG (24h) (8) G_3_: 2% green tea (24h) (8) G_4_: none (24h) (8) G_5_: 2% CHX (6 months) (8) G_6_: 2% EGCG (6 months) (8)  G_7_: 2% green tea (6 months) (8)  G_8_: none (6 months) (8)  + adhesive + resin | Third molars | 0.1% Thymol  (≤ 6 months) | Adhesive: Clearfil SE Bond Resin: Filtek Z350 | G_1_: 13.31±3.36; G_2_: 6.93±3.43;  G_3_: 10.60±4.69; G_4_: 8.64±5.52;  G_5_: 11.09±4.98; G_6_: 15.96±5.32;  G_7_: 17.82±12.20; G_8_: 16.69±7.20  G_1_/G_2_;G_3_;G_4_^*^; G_2_/G_3_;G_6_^*^; G_3_/G_7_^*^; G_4_/G_8_^*^; G_5_/G_6_;G_7_;G_8_^*^ |  |
| Gunaydin et al, 2016[122] | G_1_: 35% phosphoric acid + adhesive 1 (24h) (5)  G_2_: 35% phosphoric acid + 2% CHX + adhesive 1 (24h) (5)  G_3_: adhesive 2 (24h) (5)  G_4_: 2% CHX + adhesive 2 (24h) (5)  G_5_: adhesive 3 (24h) (5)  G_6_: 2% CHX + adhesive 3 (24h) (5)  G_7_: adhesive 4 (24h) (5)  G_8_: 2% CHX + adhesive 4 (24h) (5)  G_9_: 35% phosphoric acid + adhesive 1 (TC) (5)  G_10_: 35% phosphoric acid + 2% CHX + adhesive 1 (TC) (5)  G_11_: adhesive 2 (TC) (5)  G_12_: 2% CHX + adhesive 2 (TC) (5)  G_13_: adhesive 3 (TC) (5)  G_14_: 2% CHX + adhesive 3 (TC) (5)  G_15_: adhesive 4 (TC) (5)  G_16_: 2% CHX + adhesive 4 (TC) (5)  + resin | Third molars | Distilled water (30 days) | Adhesive: 1 - Single Bond 2; 2 - Clearfil SE Bond; 3 - Clearfil S3 Bond; 4 - Adper Prompt-L-Pop  Resin: Filtek Z250 | G_1_: 36.13±2.46; G_2_: 31.36±1.29;  G_3_: 35.38±2.26; G_4_: 32.94±1.72;  G_5_: 30.23±1.20; G_6_: 27.5±1.55;  G_7_: 27.11±0.96; G_8_: 23.69±1.12;  G_9_: 17.60±1.71; G_10_: 23.91±1.23;  G_11_: 17.97±1.31; G_12_: 23.68±1.67;  G_13_: 14.36±1.16; G_14_: 19.83±0.78;  G_15_: 12.96±0.49; G_16_: 17.20±0.65  G_1_/G_2_;G_9_^*^; G_2_/G_10_^*^; G_3_/G_4_;G_11_^*^; G_4_/G_12_^*^;  G_5_/G_6_;G_13_^*^; G_6_/G_14_^*^; G_7_/G_8_;G_15_^*^; G_8_/G_16_^*^; G_9_/G_10_^*^; G_11_/G_12_^*^; G_13_/G_14_^*^; G_15_/G_16_^*^ |  |
| Koizumi et al, 2016[99] | G_1_: 37.5% phosphoric acid + adhesive 1 (5) G_2_: adhesive 2 (5) G_3_: adhesive 3 (5) G_4_: Riva Star + 37.5% phosphoric acid + adhesive 1 (5) G_5_: Riva Star + adhesive 2 (5) G_6_: Riva Star + adhesive 3 (5)  + resin | Molars | 0.5% Chloramine | Adhesive: 1 - Optibond FL; 2 – Optibond Versa (Kerr, USA); 3 - Clearfil Liner Bond F (Kuraray, Japan) Riva Star (SDI, Australia) Resin: Aura (SDI, Australia) | G_1_: 32.1±1.2; G_2_: 35.0±3.9;  G_3_: 28.4±8.4; G_4_: 21.4±9.4;  G_5_: 9.6±2.0; G_6_: 10.8±2.1  G_1_/G_4_;G_5_;G_6_^*^; G_2_/G_4_;G_5_;G_6_^*^; G_3_/G_4_;G_5_;G_6_^*^; G_4_/G_5_;G_6_^*^ |  |
| Kucukyilmaz et al, 2016[28] | G_1_: none (12) G_2_: none (artificial caries) (12) G_3_: laser (12) G_4_: laser (artificial caries) (12) G_5_: silver diamine fluoride (12) G_6_: silver diamine fluoride (artificial caries) (12) G_7_: ammonium hexafluorosilicate (12)  G_8_: ammonium hexafluorosilicate (artificial caries) (12)  + adhesive + resin | Molars | 0.5% Thymol  (≤ 1 month) | Laser Er:YAG (Fidelis, Fotona, Slovenia) Adhesive: Clearfil SE Bond Resin: Filtek Z250 | G_1_: 36.79±5.37; G_2_: 30.10±5.88;  G_3_: 37.06±5.53; G_4_: 34.78±5.34;  G_5_: 31.87±7.54; G_6_: 18.89±2.28;  G_7_: 32.80±3.93; G_8_: 25.87±3.57  G_1_/G_2_;G_5_;G_7_^*^; G_2_/G_3_;G_4_;G_6_;G_8_^*^; G_3_/G_7_^*^; G_5_/G_6_^*^; G_6_/G_8_^*^ |  |
| Kusdemir et al, 2016[102] | G_1_: 32% phosphoric acid + 2% CHX + adhesive (3)  G_3_: 32% phosphoric acid + adhesive (3)  G_4_: 2% CHX + adhesive (3)  G_6_: adhesive (3)  + resin | Third molars | 0.5% chloramine + distilled water  (6 months) | Aqua Prep F (Bisco, USA)  Adhesive: 1 - Single Bond Universal  Resin: Filtek Z550 (3M, USA) | G_1_: 32.8±6.4; G_2_: 24.4±5.32;  G_3_: 14.1±4.1; G_4_: 21.2±4.8  G_1_/All^*^; G_2_/G_3_^*^; G_3_/G_4_^*^ |  |
| Loguercio et al, 2016[104] | 37% phosphoric acid +  G_1_: water + adhesive 1 + resin (24h) (5)  G_2_: 2% minocycline + adhesive 1 + resin (24h) (5)  G_3_: 2% CHX + adhesive 1 + resin (24h) (5)  G_4_: water + adhesive 1 + resin (24 months) (5)  G_5_: 2% minocycline + adhesive 1 + resin (24 months) (5)  G_6_: 2% CHX + adhesive 1 + resin (24 months) (5)  G_7_: water + adhesive 2 + resin (24h) (5)  G_8_: 2% minocycline + adhesive 2 + resin (24h) (5)  G_9_: 2% CHX + adhesive 2 + resin (24h) (5)  G_10_: water + adhesive 2 + resin (24 months) (5)  G_11_: 2% minocycline + adhesive 2 + resin (24 months) (5)  G_12_: 2% CHX + adhesive 2 + resin (24 months) (5) | Third molars | 0.5% Chloramine + distilled water (2-3 months) | Adhesive: 1 - Prime & Bond NT; 2 - Single Bond 2  Resin: Opallis | G_1_: 42.3±3.4; G_2_: 46.3±5.4;  G_3_: 44.2±4.3; G_4_: 23.6±5.3;  G_5_: 41.4±3.6; G_6_: 36.3±5.1;  G_7_: 46.2±4.7; G_8_: 49.6±3.6;  G_9_: 50.3±5.6; G_10_: 32.3±4.5;  G_11_: 44.2±5.1; G_12_: 43.3±3.5  G_4_/G_1_;G_2_; G_3_;G_5_;G_6_^*^; G_10_/G_7_;G_8_;G_9_;G_11_;G_12_^*^ |  |
| Loguercio et al, 2016[105] | G_1_: 37% phosphoric acid + distilled water + adhesive 1 (24h) (7)  G_2_: 2% CHX + 37% phosphoric acid + 2% CHX + adhesive 1 (24h) (7)  G_3_: 37% phosphoric acid + distilled water + adhesive 2 (24h) (7)  G_4_: 2% CHX + 37% phosphoric acid + 2% CHX + adhesive 2 (24h) (7)  G_5_: 37% phosphoric acid + distilled water + adhesive 1 (5 years) (7)  G_6_: 2% CHX + 37% phosphoric acid + 2% CHX + adhesive 1 (5 years) (7)  G_7_: 37% phosphoric acid + distilled water + adhesive 2 (5 years) (7)  G_8_: 2% CHX + 37% phosphoric acid + 2% CHX + adhesive 2 (5 years) (7)  + resin | Third molars | 0.5% Chloramine + distilled water  (6 months) | Adhesive: 1 - Prime&Bond NT; 2 –Single Bond 2  Resin: Opallis | G_1_: 35.1±3.1; G_2_: 33.1±2.8;  G_3_: 40.2±3.3; G_4_: 43.5±3.5;  G_5_: 11.0±2.7; G_6_: 22.1±2.2;  G_7_: 16.1±2.1; G_8_: 31.3±2.7  G_1_/G_5_;G_6_^*^; G_2_/G_5_;G_6_^*^; G_3_/G_7_;G_8_^*^; G_4_/G_7_;G_8_^*^; G_5_/G_6_^*^; G_7_/G_8_^*^ |  |
| Oliveira et al, 2016[103] | G_1_: none (5)  G_2_: 10% doxycycline (pH 3) (5)  G_3_: 10% doxycycline (pH 6) (5)  G_4_: 2% CHX (5)  + 35% phosphoric acid + adhesive + resin | Molars | 0.5% Chloramine | Adhesive: Single Bond 2  Resin: Filtek Z100 | G_1_: 36.4±7.43; G_2_: 35.2±7.09;  G_3_: 47.9±11.73; G_4_: 40.3±3.66 |  |
| Pucci et al, 2016[121] | 35% phosphoric acid +  G_1_: adhesive 1 + resin (24h) (6)  G_2_: adhesive 2 + resin (24h) (6)  G_3_: adhesive 3 + resin (24h) (6)  G_4_: 10% NaOCl + adhesive 1 + resin (24h) (6)  G_5_: 10% NaOCl + adhesive 2 + resin (24h) (6)  G_6_: 10% NaOCl + adhesive 3 + resin (24h) (6)  G_7_: adhesive 1 + resin (1 year) (6)  G_8_: adhesive 2 + resin (1 year) (6)  G_9_: adhesive 3 + resin (1 year) (6)  G_10_: 10% NaOCl + adhesive 1 + resin (1 year) (6)  G_11_: 10% NaOCl + adhesive 2 + resin (1 year) (6)  G_12_: 10% NaOCl + adhesive 3 + resin (1 year) (6) | Molars | Distilled water (6 months) | Adhesive: 1 - DenTASTIC UNO (Pulpdent, USA); 2 - Prime & Bond NT; 3 -Single Bond  Resin: Filtek Z100 | G_1_: 22.45±8.64; G_2_: 7.90±4.55;  G_3_: 12.56±6.54; G_4_: 19.85±8.91;  G_5_: 33.73±14.31; G_6_: 20.77±10.16;  G_7_: 2.43±0.80; G_8_: 2.26±1.89;  G_9_: 4.30±3.21; G_10_: 18.80±1.61;  G_11_: 26.49±4.23; G_12_: 22.73±3.48  G_1_/G_2_;G_3_;G_5_;G_7_;G_8_;G_9_^*^; G_2_/G_4_;G_5_;G_6_;G_7_;G_8_;G_10_;G_11_;G_12_^*^; G_3_/G_5_;G_7_;G_8_;G_11_;G_12_^*^; G_4_/G_5_;G_7_;G_8_;G_9_^*^; G_5_/G_6_;G_7_;G_8_;G_9_;G_10_;G_11_^*^; G_6_/G_7_;G_8_;G_9_^*^; G_7_/G_10_;G_11_;G_12_^*^; G_8_/G_10_;G_11_;G_12_^*^; G_9_/G_10_;G_11_;G_12_^*^ |  |
| Ruschel et al, 2016[101] | 35% phosphoric acid + adhesive +  G_1_: resin (24h) (5)  G_2_: resin (3 months) (5)  G_3_: laser + adhesive + resin (24h) (5)  G_4_: laser + adhesive + resin (3 months) (5) | Third molars | 0.5% Chloramine (24h) + distilled water (24h) | Laser Nd:YAG  Adhesive: Single Bond 2  Resin: Filtek Z250 | G_1_: 31.68±5.14; G_2_: 37.88±5.04  G_3_: 35.32±8.79; G_4_: 31.53±9.01 |  |
| Sinha et al, 2016[65] | G_1_: none (20) G_2_: 2% CHX (20) G_3_: aloe vera (20)  + 37% phosphoric acid + adhesive + resin | Lower molars | 0.1% Thymol | Adhesive: Single Bond 2 Resin: Filtek Z350 | G_1_: 18.91±0,08; G_2_: 20.66±0.05;  G_3_: 20.08±0.05 |  |
| Tekçe et al, 2016[100] | G_1_: adhesive 1 (24h) (5)  G_2_: 37% phosphoric acid + adhesive 1 (24h) (5)  G_3_: 37% phosphoric acid + 1% benzalkonic chloride + adhesive 1 (24h) (5)  G_4_: 37% phosphoric acid + 2% CHX + adhesive 1 (24h) (5)  G_5_: 0.5M EDTA + adhesive 1 (24h) (5)  G_6_: adhesive 1 (12 months) (5)  G_7_: 37% phosphoric acid + adhesive 1 (12 months) (5)  G_8_: 37% phosphoric acid + 1% benzalkonic chloride + adhesive 1 (12 months) (5)  G_9_: 37% phosphoric acid + 2% CHX + adhesive 1 (12 months)  G_10_: 0.5M EDTA + adhesive 1 (12 months) (5)  G_11_: adhesive 2 (24h) (5)  G_12_: 37% phosphoric acid + adhesive 2 (24 h) (5)  G_13_: 37% phosphoric acid + 1% benzalkonic chloride + adhesive 2 (24h) (5)  G_14_: 37% phosphoric acid + 2% CHX + adhesive 2 (24h) (5)  G_15_: 0.5M EDTA + adhesive 2 (24h) (5)  G_16_: adhesive 2 (12 months) (5)  G_17_: 37% phosphoric acid + adhesive 2 (12 months) (5)  G_18_: 37% phosphoric acid + 1% benzalkonic chloride + adhesive 2 (12 months) (5)  G_19_: 37% phosphoric acid + 2% CHX + adhesive 2 (12 months) (5)  G_20_: 0.5M EDTA + adhesive 2 (12 months) (5)  + resin | Third molars | 0.5% Chloramine  (2 weeks) | Adhesive: 1 - Single Bond Universal (Bisco, USA); 2 - All Bond Universal (Bisco, USA)  Resin: Filtek Ultimate Universal (3M, USA) | G_1_: 36.09±4.46; G_2_: 34.81±7.09;  G_3_: 43.33±3.41; G_4_: 37.67±3.40;  G_5_: 45.55±2.09; G_6_: 35.07±3.64;  G_7_: 45.22±6.32; G_8_: 41.19±3.98;  G_9_: 43.60±3.78; G_10_: 43.97±5.97;  G_11_: 38.36±8.43; G_12_: 30.07±6.10;  G_13_: 43.81±3.61; G_14_: 38.54±6.19;  G_15_: 46.59±4.98; G_16_: 39.51±6.66;  G_17_: 38.92±4.01; G_18_: 31.37±5.97;  G_19_: 46.29±5.92; G_20_: 38.13±4.47  G_1_/G_5_;G_7_^*^; G_2_/G_10_^*^; G_5_/G_6_;G_11_;G_13_;G_15_;G_17_;G_19_^*^; G_6_/G_10_^*^; G_7_/G_11_;G_13_;G_15_;G_17_,G_19_^*^; G_10_/G_12_;G_14_;G_16_;G_18_;G_20_^*^;  G_11_/G_12_^*^; G_17_/G_18_^*^; G_19_/G_20_^*^ |  |
| Bravo et al, 2017[123] | 37% phosphoric acid +  G_1_: 2% CHX + adhesive 1 + resin (72h) (5)  G_2_: adhesive 1 + resin (72h) (5)  G_3_: 2% CHX + adhesive 2 + resin (72h) (5)  G_4_: adhesive 2 + resin (72h) (5)  G_5_: 2% CHX + adhesive 3 + resin (72h) (5)  G_6_: adhesive 3 + resin (72h) (5)  G_7_: 2% CHX + adhesive 1 + resin (3 months) (5)  G_8_: adhesive 1 + resin (3 months) (5)  G_9_: 2% CHX + adhesive 2 + resin (3 months) (5)  G_10_: adhesive 2 + resin (3 months) (5)  G_11_: 2% CHX + adhesive 3 + resin (3 months) (5)  G_12_: adhesive 3 + resin (3 months) (5)  G_13_: 2% CHX + adhesive 1 + resin (6 months) (5)  G_14_: adhesive 1 + resin (6 months) (5)  G_15_: 2% CHX + adhesive 2 + resin 6 months) (5)  G_16_: adhesive 2 + resin (6 months) (5)  G_17_: 2% CHX + adhesive 3 + resin (6 months) (5)  G_18_: adhesive 3 + resin (6 months) (5) | Third molars | Distilled water | Adhesive: 1 - Scotchbond 1 XT; 2 - Adper Prompt L-Pop; 3 - Single Bond Universal  Resin: Filtek Z350 | G_1_: 26.28±9.29; G_2_: 28.56±5.83;  G_3_: 24.21±7.52; G_4_: 20.14±4.87;  G_5_: 28.43±9.78; G_6_: 29.24±7.90;  G_7_: 32.26±10.33; G_8_: 19.82±7.65;  G_9_: 28.51±13.18; G_10_: 20.86±6.13;  G_11_: 44.11±12.09; G_12_: 23.54±12.09;  G_13_: 31.73±5.18; G_14_: 23.39±5.69;  G_15_: 27.37±4.40; G_16_: 20.51±5.66;  G_17_: 36.88±6.65; G_18_: 23.62±7.07  G_1_/G_4_^*^; G_2_/G_4_;G_8_;G_14_^*^; G_4_/G_5_;G_6_^*^; G_5_/G_11_;G_17_^*^; G_7_/G_8_;G_10_;G_11_^*^; G_8_/G_11_^*^; G_9_/G_11_^*^; G_10_/G_11_^*^; G_11_/G_12_^*^; G_13_/G_14_;G_16_;G_18_^*^; G_14_/G_17_^*^; G_15_/G_16_;G_17_^*^; G_16_/G_17_^*^; G_17_/G_18_^*^ |  |
| Bravo et al, 2017[124] | 37% phosphoric acid +  G_1_: 2% CHX + adhesive 1 (72h) (8) G_2_: adhesive 1 (72h) (8) G_3_: 2% CHX + adhesive 2 (72h) (8) G_4_: adhesive 2 (72h) (8) G_5_: 2% CHX + adhesive 3 (72h) (8) G_6_: adhesive 3 (72h) (8) G_7_: 37% phosphoric acid + 2% CHX + adhesive 1 (3 months) (8) G_8_: adhesive 1 (3 months) (8) G_9_: 2% CHX + adhesive 2 (3 months) (8) G_10_: adhesive 2 (3 months) (8) G_11_: 2% CHX + adhesive 3 (3 months) (8) G_12_: adhesive 3 (6 months) (8) G_13_: 37% phosphoric acid + 2% CHX + adhesive 1 (6 months) (8) G_14_: adhesive 1 (6 months) (8) G_15_: 2% CHX + adhesive 2 (6 months) (8) G_16_: adhesive 2 (6 months) (8) G_17_: 2% CHX + adhesive 3 (6 months) (8) G_18_: adhesive 3 (6 months) (8)  + resin | Third molars | Distilled water | Adhesive: 1 - Scotchbond 1 XT;2 - Adper Prompt L-Pop; 3 - Single Bond Universal  Resin: Filtek Z350 | G_1_: 28.98±4.99; G_2_: 24.87±3.20;  G_3_: 19.43±6.08; G_4_: 17.13±3.56;  G_5_: 24.61±2.68; G_6_: 25.39±2.38;  G_7_: 20.91±4.57; G_8_: 12.42±4.32;  G_9_: 22.66±5.30; G_10_: 14.26±3.66;  G_11_: 38.78±4.18; G_12_: 28.28±4.04;  G_13_: 16.57±1.19; G_14_: 8.05±2.33;  G_15_: 17.59±2.19; G_16_: 7.48±1.13;  G_17_: 22.71±1.89; G_18_: 11.70±2.21  G_1_/G_3_;G_4_;G_7_;G_13_^*^; G_2_/G_4_;G_8_;G_14_^*^; G_4_/G_5_;G_6_;G_16_^*^; G_5_/G_11_^*^; G_6_/G_18_^*^; G_7_/G_8_;G_10_;G_11_;G_12_^*^; G_8_/G_9_;G_11_;G_12_;G_14_^*^; G_9_/G_10_;G_11_^*^; G_10_/G_11_;G_12_;G_16_^*^; G_11_/G_12_;G_17_^*^; G_12_/G_18_^*^; G_13_/G_14_;G_16_;G_17_;G_18_^*^; G_14_/G_15_;G_17_;G_18_^*^; G_15_/G_16_^*^; G_16_/G_17_;G_18_^*^; G_17_/G_18_ |  |
| Karadas et al, 2017[38] | G_1_: adhesive 1 + TC (superficial dentin) (10) G_2_: laser (10 Hz, 1.2 W, 18.9 J/cm) + adhesive 1 (TC) (superficial dentin) (10) G_3_: laser (5 Hz, 0.5 W, 15.7 J/cm) + adhesive 1 (TC) (superficial dentin) (10) G_4_: adhesive 2 (TC) (superficial dentin) (10) G_5_: laser (10 Hz, 1.2 W, 18.9 J/cm) + adhesive 2 (TC) (superficial dentin) (10) G_6_: laser (5 Hz, 0.5 W, 15.7 J/cm) + adhesive 2 (TC) (superficial dentin) (10) G_7_: adhesive 1 (TC) (deep dentin) (10) G_8_: laser (10 Hz, 1.2 W, 18.9 J/cm) + adhesive 1 (TC) (deep dentin) (10) G_9_: laser (5 Hz, 0.5 W, 15.7 J/cm) + adhesive 1 (TC) (deep dentin) (10) G_10_: adhesive 2 (TC) (deep dentin) (10) G_11_: laser (10 Hz, 1.2 W, 18.9 J/cm) + adhesive 2 (TC) (deep dentin) (10) G_12_: laser (5 Hz, 0.5 W, 15.7 J/cm) + adhesive 2 (TC) (deep dentin) (10)  G_13_: adhesive 1 (superficial dentin) (24h) (10) G_14_: laser (10 Hz, 1.2 W, 18.9 J/cm) + adhesive 1 (superficial dentin) (24h) (10) G_15_: laser (5 Hz, 0.5 W, 15.7 J/cm) + adhesive 1 (superficial dentin) (24h) (10) G_16_: adhesive 2 (superficial dentin) (24h) (10) G_17_: laser (10 Hz, 1.2 W, 18.9 J/cm) + adhesive 2 (superficial dentin) (24h) (10) G_18_: laser (5 Hz, 0.5 W, 15.7 J/cm) + adhesive 2 (superficial dentin) (24h) (10) G_19_: adhesive 1 (deep dentin) (24h) (10) G_20_: laser (10 Hz, 1.2 W, 18.9 J/cm) + adhesive 1 (deep dentin) (24h) (10) G_21_: laser (5 Hz, 0.5 W, 15.7 J/cm) + adhesive 1 (deep dentin) (24h) (10) G_22_: adhesive 2 (deep dentin) (24h) (10) G_23_: laser (10 Hz, 1.2 W, 18.9 J/cm) + adhesive 2 (deep dentin) (24h) (10) G_24_: laser (5 Hz, 0.5 W, 15.7 J/cm) + adhesive 2 (deep dentin) (24h) (10) | Third molars | 0.5% Chloramine (2 weeks) | Laser Er:YAG (LightWalker; Fotona, Slovenia)  Adhesive: 1 - Clearfil SE Bond; 2 - Clearfil S3 Bond | G_1_: 37.13±8.1; G_2_: 38.61±8.1;  G_3_: 29.24±10.6; G_4_: 29.80±9.3;  G_5_: 36.02±11.5; G_6_: 31.83±8.2;  G_7_: 8.68±4.1; G_8_: 22.53±3.7;  G_9_: 23.83±7.5; G_10_: 3.31±2.5;  G_11_: 16.93±3.9; G_12_: 19.54±4.3; G_13_: 33.48±8.1; G_14_: 40.93±7.1;  G_15_: 29.49±13.4; G_16_: 23.11±5.7;  G_17_: 33.57±5.9; G_18_: 30.02±6.1;  G_19_: 17.42±4.1; G_20_: 28.51±4.3;  G_21_: 31.33±6.7; G_22_: 9.90±2.9;  G_23_: 26.20±5.8; G_24_: 28.46±4.9  G_1_/G_7_^*^; G_2_/G_8_^*^; G_3_/G_9_^*^; G_4_/G_10_^*^; G_5_/G_11_^*^; G_6_/G_12_^*^; G_7_/G_8_;G_9_;G_11_;G_12_^*^; G_8_/G_10_^*^; G_9_/G_10_^*^; G_10_/G_11_;G_12_^*^; G_13_/G_16_;G_19_^*^; G_14_/G_15_;G_16_;G_20_^*^; G_15_/G_18_^*^; G_16_/G_22_^*^; G_17_/G_23_^*^; G_19_/G_20_;G_21_;G_22_;G_23_;G_24_^*^; G_20_/G_22_^*^; G_21_/G_22_^*^; G_22_/G_23_;G_24_^*^ |  |
| Kim et al, 2017[70] | G_1_: distilled water (5)  G_2_: 2% CHX (5)  G_3_: 6% NaOCl (5)  G_4_: 0.01% urushiol (5)  + 37% phosphoric acid + adhesive + resin | Molars | 0.1% Thymol | Adhesive: Scotchbond Universal  Resin: Filtek Z350 | G_1_: 33.12±4.05; G_2_: 30.98±4.53;  G_3_: 25.86±4.46; G_4_: 32.95±6.70  G_1_/G_3_^*^; G_2_/G_4_^*^ |  |
| Kucukyilmaz et al, 2017[107] | G_1_: 37% phosphoric acid (20) G_2_: laser (MSP) (20) G_3_: laser (QSP) (20)  + adhesive + resin | Molars | Chloramine | Laser Er:YAG (LightWalker, Fotona, Slovenia)  Adhesive: Optibond FL Resin: Filtek Z250 | G_1_: 16.61±1.99; G_2_: 1.24±1.0;  G_3_: 10.77±1.07  G_1_/G_2_^*^; G_1_/G_3_^*^ |  |
| Neri et al, 2017[69] | G_1_: distilled water (24h) (3)  G_2_: 2% CHX (24h) (3)  G_3_: 1.23% sodium fluoride (24h) (3)  G_4_: distilled water (TC) (3)  G_5_: 2% CHX (TC) (3)  G_6_: 1.23% sodium fluoride (TC) (3)  + adhesive + resin | Third molars | 0.01 Thymol  (1 month) | Adhesive: Clearfil SE Bond  Resin: Filtek Z250 | G_1_: 30.98±5.96; G_2_: 30.18±4.06;  G_3_: 35.60±6.52; G_4_: 22.96±6.88;  G_5_: 21.91±4.37; G_6_: 30.17±4.23  G_1_/G_4_^*^; G_2_/G_5_^*^; G_4_/G_6_^*^; G_5_/G_6_^*^ |  |
| Rechmann et al, 2017[71] | G_1_: 35% phosphoric acid + adhesive 1 (10) G_2_: laser (7μs/0.25mm; 6.0 J/cm^2^) + 35% phosphoric acid + adhesive 1 (10) G_3_: laser (23μs/0.25mm; 17.4 J/cm^2^) + 35% phosphoric acid + adhesive 1 (10) G_4_: laser (43μs/0.25mm; 32.4 J/cm^2^) + 35% phosphoric acid + adhesive 1 (10) G_5_: laser (63μs/0.25mm; 47.7 J/cm^2^) + 35% phosphoric acid + adhesive 1 (10) G_6_: 37.5% phosphoric acid + adhesive 2 (10) G_7_: laser (7μs/0.25mm; 6.0 J/cm^2^) + 37.5% phosphoric acid + adhesive 2 (10) G_8_: laser (23μs/0.25mm; 17.4 J/cm^2^) + 37.5% phosphoric acid + adhesive 2 (10) G_9_: laser (43μs/0.25mm; 32.4 J/cm^2^) + 37.5% phosphoric acid + adhesive 2 (10) G_10_: laser (63μs/0.25mm; 47.7 J/cm^2^) + 37.5% phosphoric acid + adhesive 2 (10) G_11_: adhesive 3 (10) G_12_: laser (7μs/0.25mm; 6.0 J/cm2) + adhesive 3 (10) G_13_: laser (23μs/0.25mm; 17,4 J/cm2) + adhesive 3 (10) G_14_: laser (43μs/0.25mm; 32,4 J/cm2) + adhesive 3 (10) G_15_: laser (63μs/0.25mm; 47,7 J/cm2) + adhesive 3 (10) G_16_: adhesive 1 (10) G_17_: laser (7μs/0.25mm; 6.0 J/cm2) + adhesive 1 (10) G_18_: laser (23μs/0.25mm; 17,4 J/cm2) + adhesive 1 (10) G_19_: laser (43μs/0.25mm; 32,4 J/cm2) + adhesive 1 (10) G_20_: laser (63μs/0.25mm; 47,7 J/cm2) + adhesive 1 (10)  + resin | Molars | 0.1% Thymol | Laser CO_2_ (Solea, Convergent Dental, USA)  Adhesive: 1 - Peak Universal Bond (Ultradent, USA); 2 - OptiBond Solo Plus; 3 - Scotchbond Universal Resin: Clearfil AP-X; | G_1_: 63.02±7.56; G_2_: 40.97±7.78;  G_3_: 36.85±8.82; G_4_: 36.02±9.11;  G_5_: 36.02±7.44; G_6_: 31.58±6.02;  G_7_: 18.91±3.32; G_8_: 19.15±3.49;  G_9_: 19.98±5.25; G_10_: 20.11±3.87;  G_11_: 58.79±10.94; G_12_: 57.07±7.2;  G_13_: 38.15±5.44; G_14_: 43.90±5.71;  G_15_: 43.94±6.47; G_16_: 33.15±3.55;  G_17_: 28.46±4.65; G_18_: 20.82±5.98;  G_19_: 21.54±5.7; G_20_: 25.27±3.8  G_1_/G_2_;G_3_;G_4_;G_5_^*^; G_6_/G_7_;G_8_;G_9_;G_10_^*^; G_11_/G_13_;G_14_;G_15_^*^; G_12_/G_13_;G_14_^*^; G_13_/G_14_;G_15_^*^; G_16_/G_18_^*^; G_16_/G_19_^*^; G_17_/G_19_;G_20_^*^; G_18_/G_20_^*^; G_19_/G_20_^*^ |  |
| Sharafeddin et al, 2017[106] | G_1_: 37% phosphoric acid + adhesive 1 + resin 1 (10) G_2_: 5% NaOCl + 37% phosphoric acid + adhesive 1 + resin 1 (10) G_3_: 37% phosphoric acid + 4% TiF_4_ + adhesive 1 + resin 1 (10)  G_4_: adhesive 2 + resin 2 (10)  G_5_: 5% NaOCl + adhesive 2 + resin 2 (10)  G_6_: 4% TiF_4_ + adhesive 2 + resin 2 (10) | Maxillary premolars | 0.5% Chloramine  (24 h) + distilled water | Adhesive: 1 - Single Bond; 2 - Silorane System Adhesive  Resin: 1 – Filtek Z350; 2 – Filtek P90 (3M, USA) | G_1_: 21.43±2.03; G_2_: 18.59±2.29;  G_3_: 21.1±0.64; G_4_: 18.1±1.79;  G_5_: 15.9±1.22; G_6_: 20.7±1.19  G_1_/G_2_^*^; G_1_/G_4_;G_5_^*^; G_2_/G_3_^*^; G_2_/G_5_^*^; G_2_/G_6_^*^; G_3_/G_4_;G_5_^*^; G_4_/G_5_^*^; G_4_/G_6_^*^; G_5_/G_6_^*^ |  |
| Suma et al, 2017[155] | G_1_: none (12) G_2_: 2% CHX (12) G_3_: Ora-5 (12)  + adhesive + resin | Third molars | N/A | Adhesive: Adper Prompt Ora-5  Resin: Filtek Z350 | G_1_: 14.46±1.31; G_2_: 10.72±2.20;  G_3_: 9.76±2.02  G_1_/G_2_^*^; G_1_/G_3_^*^ |  |
| Alaghehmad et al, 2018[72] | G_1_: none (10) G_2_: 0.12% CHX (10) G_3_: none (TC) (10) G_4_: 0.12% CHX (TC) (10)  + 37% phosphoric acid + adhesive + resin | Molars | 0.2% Thymol (24h) | Adhesive: Single Bond 2 Resin: Filtek Z250 | G_1_: 15.02±4.76; G_2_: 13.45±4.7;  G_3_: 14.4±4.06; G_4_: 12.52±4.14; |  |
| Jowkar et al, 2018[108] | G_1_: none (10) G_2_: silver nanoparticles (10) G_3_: zinc oxide nanoparticles (10) G_4_: titanium nanoparticles (10)  + phosphoric acid + adhesive + resin | Molars | 0.5% Chloramine  (≤1 month) | Adhesive: Single Bond Resin: Filtek Z250 | G_1_: 13.70±5.89; G_2_: 25.60±14.61;  G_3_: 14.76±10.44; G_4_: 8.80±3.07  G_2_/G_3_^*^; G_2_/G_4_^*^ |  |
| Kalaiselvam et al, 2018[137] | G_1_: PBS + adhesive 1 (24h) (6)  G_2_: PBS + 37% phosphoric acid + adhesive 2 (24h) (6)  G_3_: 5% EGCG + adhesive 1 (24h) (6)  G_4_: 5% EGCG + 37% phosphoric acid + adhesive 2 (24h) (6)  G_5_: 2% CHX + adhesive 1 (24h) (6)  G_6_: 2% CHX + 37% phosphoric acid + adhesive 2 (24h) (6)  G_7_: catechin + adhesive 1 (24h) (6)  G_8_: catechin + 37% phosphoric acid + adhesive 2 (24h) (6)  G_9_: PBS + adhesive 1 (TC) (6)  G_10_: PBS + 37% phosphoric acid + adhesive 2 (TC) (6)  G_11_: 5% EGCG + adhesive 1 (TC) (6)  G_12_: 5% EGCG + 37% phosphoric acid + adhesive 2 (TC) (6)  G_13_: 2% CHX + adhesive 1 (TC)  G_14_: 2% CHX + 37% phosphoric acid + adhesive 2 (TC) (6)  G_15_: catechin + adhesive 1 (TC) (6)  G_16_: catechin + 37% phosphoric acid + adhesive 2 (TC) (6)  + adhesive + resin | Lower third molars | 0.9% NaCl + 0.05% sodium azide (4ºC) | Adhesive: 1 - Easy Bond; 2 - Single Bond 2  Resin: Filtek Z250 | G_1_: 37.59±5.27; G_2_: 41.56±4.56;  G_3_: 57.92±3.34; G_4_: 56.35±6.40;  G_5_: 47.40±7.41; G_6_: 51.83±3.04;  G_7_: 47.24±3.38; G_8_: 46.06±9.78;  G_9_: 28.29±4.89; G_10_: 28.67±1.46;  G_11_: 56.46±12.79; G_12_: 43.73±1.96;  G_13_: 45.81±5.56; G_14_: 34.70±7.65;  G_15_: 35.70±5.07; G_16_: 34.22±4.93 |  |
| Sinha et al, 2018[134] | G_1_: none (20) G_2_: 2% CHX (20) G_3_: aloe vera (20) G_4_: none (6 months) (20) G_5_: 2% CHX (6 months) (20) G_6_: aloe vera (6 months) (20)  + 37% phosphoric acid + adhesive + resin | Molars | Saline (4ºC,  ≤ 2 weeks) | Adhesive: Single Bond 2 Resin: Filtek Z350 | G_1_: 16.77±0.83; G_2_: 19.59±0.47;  G_3_: 19.41±0.86; G_4_: 13.91±0.83;  G_5_: 16.53±0.49; G_6_: 16.42±0.55  G_1_/G_2_;G_3_^*^; G_5_/G_6_^*^ |  |
| Sun et al, 2018[136] | 37% phosphoric acid +  G_1_: none (24h) (10)  G_2_: 0.02% EGCG (24h) (10)  G_3_: 0.1% EGCG (24h) (10)  G_4_: 0.5% EGCG (24h) (10)  G_5_: none (TC) (10)  G_6_: 0.02% EGCG (TC) (10)  G_7_: 0.1% EGCG (TC) (10)  G_8_: 0.5% EGCG (TC) (10)  adhesive + resin + TC | Third molars | 0.9% NaCl + 0.02% sodium azide (4ºC, 1 month) | Adhesive: Single Bond 2  Resin: Clearfil AP-X | G_1_: 14.2±4.6; G_2_: 14.6±4.4;  G_3_: 14.7±3.8; G_4_: 9.2±3.0;  G_5_: 7.1±3.1; G_6_: 10.7±3.7;  G_7_: 12.6±3.4; G_8_: 5.2±2.9  G_1_/G_4_;G_5_^*^; G_2_/G_4_;G_6_^*^; G_3_/G_4_;G_7_^*^; G_4_/G_8_^*^ |  |
| Akturk et al, 2019[157] | G_1_: adhesive 1 (10)  G_2_: 2% CHX + adhesive 1 (10)  G_3_: ozonated water + adhesive 1 (10)  G_4_: 5% boric acid + adhesive 1 (10)  G_5_: adhesive 2 (10)  G_6_: 2% CHX + adhesive 2 (10)  G_7_: ozonated water + adhesive 2 (10)  G_8:_ 5% boric acid + adhesive 2 (10)  G_9_: adhesive 3 (10)  G_10_: 2% CHX + adhesive 3 (10)  G_11_: ozonated water + adhesive 3 (10)  G_12_: 5% boric acid + adhesive 3 (10)  + resin | Molars | N/A | Adhesive: 1 - Clearfil SE Bond; 2 - OptiBond XTR (Kerr, USA); 3 - Tokuyama Universal (Tokuyama Dental, Japan)  Resin: Estelite Quick (Tokuyama Dental, Japan) | G_1_: 20.33±5.53; G_2_: 31.85±6.33;  G_3_: 27.05±4.03; G_4_: 23.17±4.67;  G_5_: 18.99±3.53; G_6_: 27.14±5.22;  G_7_: 22.94±2.66; G_8_: 19.00±6.34;  G_9_: 18.93±3.61; G_10_: 20.70±6.22;  G_11_: 21.99±5.02; G_12_: 15.64±2.48  G_1_/G_2_;G_3_^*^; G_2_/G_3_;G_4;_G_10_^*^; G_3_/G_4;_G_11;_G_12_^*^; G_5_/G_6_;G_7;_G_8_^*^; G_9_/G_10;_G_11;_G_12_^*^ |  |
| Hussein et al, 2019[75] | 37% phosphoric acid +  G_1_: none (24)  G_2_: 2% CHX (24)  G_3_: 100% ethanol (24)  + adhesive + resin | Maxillary second premolars | 0.1% Thymol  (2 weeks) | Adhesive: Single Bond Universal  Resin: Filtek Z350 | 24h  G_1_: 8.38±1.34; G_2_: 8.42±1.15;  G_3_: 8.66±0.96  3 months  G_1_: 7.15±0.90; G_2_: 8.00±1.57;  G_3_: 7.70±1.56  6 months  G_1_: 5.88±0.62; G_2_: 7.42±0.77;  G_3_: 7.43±1.08  G_1_/All^*^ |  |
| Jowkar et al, 2019[73] | G_1_: adhesive 1 (24h) (10)  G_2_: phosphoric acid + 2% CHX + adhesive 1 (24h) (10)  G_3_: phosphoric acid + silver nanoparticles + adhesive 1 (24h) (10)  G_4_: adhesive 2 (24h) (10)  G_5_: phosphoric acid + 2% CHX + adhesive 2 (24h) (10)  G_6_: phosphoric acid + silver nanoparticles + adhesive 2 (24h) (10)  G_7_: adhesive 1 (6 months) (10)  G_8_: phosphoric acid + 2% CHX + adhesive 1 (6 months) (10)  G_9_: phosphoric acid + silver nanoparticles + adhesive 1 (6 months) (10)  G_10_: adhesive 2 (6 months) (10)  G_11_: phosphoric acid + 2% CHX + adhesive 2 (6 months) (10)  G_12_: phosphoric acid + silver nanoparticles + adhesive 2 (6 months) (10)  + resin | Third molars | 0.5% Thymol  (1 month) | Adhesive: 1 - Single Bond 2; 2 - Clearfil SE Bond  Resin: Filtek Z250 | G_1_: 20.7±1.9; G_2_: 23.0±1.7;  G_3_: 23.7±1.8; G_4_: 21.3±1.9;  G_5_: 23.1±1.5; G_6_: 27.0±0.8;  G_7_: 14.1±0.9; G_8_: 18.8±1.1;  G_9_: 19.4±1.0; G_10_: 15.7±1.0;  G_11_: 20.3±1.5; G_12_: 25.0±1.1  G_1_/G_2_;G_3_;G_5_;G_6_;G_7_^*^; G_2_/G_4_;G_8_;G_6_^*^; G_3_/G_4_;G_6_;G_9_^*^; G_4_/G_5_;G_6_;G_10_^*^; G_5_/G_6_;G_11_^*^; G_6_/G_12_^*^; G_7_/G_8_;G_9_;G_10_;G_11_;G_12_^*^; G_8_/G_10_;G_12_^*^; G_9_/G_10_;G_12_^*^; G_10_/G_11_;G_12_^*^; G_11_/G_12_^*^ |  |
| Kasraei et al, 2019[76] | G_1_: 35% phosphoric acid + adhesive (18) G_2_: laser + 35% phosphoric acid + adhesive (18) G_3_: 35% phosphoric acid + laser + adhesive (18) G_4_: 35% phosphoric acid + adhesive + laser (18) G_5_: 35% phosphoric acid + laser + adhesive + laser (18)  + resin | Premolars | 0.2% Thymol  (1 week) | Laser diode (Epic 10, Biolase, USA)  Adhesive: Single Bond Resin: Filtek Z250 | G_1_: 18.85±4.79; G_2_: 23.39±6.07;  G_3_: 22.85±5.11; G_4_: 38.35±8.99;  G_5_: 25.16±6.14  G_1_/G_4_;G_5_^*^; G_2_/G_4_^*^; G_3_/G_4_^*^ |  |
| Rayar et al, 2019[148] | G_1_: 37% phosphoric acid + adhesive 1 (20) G_2_: adhesive 2 (20) G_3_: 37% phosphoric acid + 2% CHX + adhesive 1 (20) G_4_: 2% CHX + adhesive 2 (20)  + resin + TC | Premolars | 10% Formaldehyde | Adhesive: 1 – Total-etch; 2 – Self-etch Resin: Filtek Z350 | G_1_: 28.25±2.53; G_2_: 25.15±3.28;  G_3_: 46.2±2.55; G_4_: 29,45±1.76  G_1_/G_2;_G_3_^*^; G_2_/G_3_;G_4_^*^; G_3_/G_4_^*^ |  |
| Sharafeddin et al, 2019[74] | 37% phosphoric acid +  G_1_: none (10)  G_2_: 5% NaOCl (10)  G_3_: 4% TiF_4_ (10)  + adhesive + resin | Maxillary premolars | 0.5% Thymol | Adhesive: Single Bond  Resin: Filtek Z350 | G_1_: 15.45±3.55; G_2_: 15.35±4.49;  G_3_: 17.01±4.85 |  |
| Silva et al, 2019[109] | G_1_: adhesive 1 (8)  G_2_: grape seed extract + adhesive 1 (8)  G_3_: adhesive 2 (8)  G_4_: grape seed extract + adhesive 2 (8)  + resin + TC | Molars and premolars | 0.5% Chloramine | Adhesive: 1 - Scotchbond Universal; 2 - Clearfil SE Bond  Resin: GrandioSO (VOCO, Germany) | G_1_: 20.04±9.71; G_2_: 14.80±8.85;  G_3_: 24.27±10.28; G_4_: 22.52±10.3 |  |
| Trevelin et al, 2019[125] | G_1_: none (24h) (15)  G_2_: laser (50 µs) (24h) (15)  G_3_: laser (300 µs) (24h) (15)  G_4_: laser (600 µs) (24h) (15)  G_5_: none (12 months) (15)  G_6_: laser (50 µs) (12 months) (15)  G_7_: laser (300 µs) (12 months) (15)  G_8_: laser (600 µs) (12 months) (15)  + adhesive + resin | Molars | Distilled water (3 months) | Laser Er:YAG (Fidelis ERIII 1000 model, Fotona, Slovenia)  Adhesive: Scotchbond Universal  Resin: Filtek Z350 | G_1_: 26.17±3.78; G_2_: 22.14±2.86;  G_3_: 21.25±3.04; G_4_: 20.62±1.86;  G_5_: 24.97±4.70; G_6_: 22.85±2.95;  G_7_: 22.13±2.99; G_8_: 23.01±2.94  G_1_/G_2_;G_3_;G_4_;G_6_;G_7_;G_8_^*^; G_2_/G_5_^*^; G_3_/G_5_^*^; G_4_/G_5_^*^ |  |
| Wang et al, 2019[110] | G_1_: distilled water (5)  G_2_: 35% phosphoric acid (5)  G_3_: 5% NaOCl (5)  G_4_: 15% EDTA (5)  G_7_: 35% phosphoric acid + 5% NaOCl (5)  G_8_: 35% phosphoric acid + 10% NaOCl (5)  + adhesive + resin | Premolars | Chloramine  (1 month) | Adhesive: Easy One  Resin: Filtek Z250 | G_1_: 11.94±0.99; G_2_: 15.82±1.44;  G_3_: 12.95±1.38; G_4_: 12.23±1.03;  G_7_: 16.88±1.85; G_8_: 16.42±0.70  G_1_/G_2_;G_7_;G_8_^*^; G_2_/G_3_;G_4_^*^: G_3_/G_7_;G_8_^*^; G_4_/G_7_;G_8_^*^ |  |
| Zabeu et al, 2019[156] | G_1_: phosphoric acid + adhesive 1 + resin (5) G_2_: phosphoric acid + adhesive 1 (primer) + laser + adhesive 1 (bond) + resin (5) G_3_: phosphoric acid + adhesive 1 + laser + resin (5) G_4_: adhesive 2 + resin (5) G_5_: adhesive 2 (primer) + laser + adhesive 2 (bond) + resin (5) G_6_: adhesive 2 + laser + resin (5) G_7_: phosphoric acid + adhesive 1 + resin (12 months) (5) G_8_: phosphoric acid + adhesive 1 (primer) + laser + adhesive 1 (bond) + resin (12 months) (5) G_9_: phosphoric acid + adhesive 1 + laser + resin (12 months) (5) G_10_: adhesive 2 + resin (12 months) (5) G_11_: adhesive 2 (primer) + laser + adhesive 2 (bond) + resin (12 months) (5) G_12_: adhesive 2 + laser + resin (12 months) (5) | Third molars | N/A | Laser: diode (SiroLaser, Sirona; Germany)  Adhesive: 1 - Scotchbond Multi-Purpose; 2 - Clearfil SE Bond Resin: Filtek Z250 | G_1_: 46.55±10.69; G_2_: 44.34±6.43;  G_3_: 37.20±12.96; G_4_: 31.47±8.41;  G_5_: 39.62±11.39; G_6_: 40.10±11.36;  G_7_: 37.65±10.66; G_8_: 28.86±16.46;  G_9_: 20.06±16.90; G_10_: 25.91±10.24;  G_11_: 24.13±10.75; G_12_: 22.08±11.77  G_1_/G_3_;G_4_;G_6_;G_7_;G_12_^*^; G_2_/G_8_^*^; G_3_/G_9_^*^; G_4_/G_10_^*^; G_5_/G_11_^*^; G_6_/G_12_^*^; G_7_/G_9_;G_10_^*^ |  |
| Davalloo et al, 2020[13] | phosphoric acid +  G_1_: none (15) (6 months)  G_2_: 2% CHX (15) (6 months)  G_3_: Gluma (15) (6 months)  + adhesive + resin | Premolars | 0.5% Chloramine + distilled water (4ºC) | Adhesive: Ambar  Resin | G_1_: 9.08±1.03; G_2_: 10.67±1.03;  G_4_: 12.43±0.52  G_1_/G_3_^*^ |  |
| Deeb et al, 2020[14] | G_1_: none (10)  G_2_: 2% CHX (infected dentin) (10)  G_3_: laser (infected dentin) (10)  + adhesive + resin + TC | Lower teeth | Chloramine (4ºC, 48h) + distilled water | Laser :YSGG (Waterlase, Biolase, USA)  Adhesive: Adper Prompt  Resin: Filtek Z350 | G_1_: 24.98±1.59; G_2_: 18.25±1.29;  G_3_: 15.89±3.22  G_1_/All; G_2_/G_3_^*^ |  |
| Fernandes et al, 2020[16] | G_1_: none (5)  G_2_: 37% phosphoric acid (5)  G_3_: 2% CHX (5)  G_4_: 37% phosphoric acid + 2% CHX (5)  + adhesive + resin | Molars | N/A | Adhesive: Single Bond Universal  Resin: Filtek Z250 | G_1_: 29.26±9.06; G_2_: 32.07±11.70;  G_3_: 20.83±8.72; G_4_: 29.41±11.31 |  |
| Mapar et al, 2020[15] | 37% phosphoric acid +  G_1_: none  G_2_: 2% CHX  + adhesive + resin + TC | Premolars | 0.5% thymol | Adhesive: Single Bond 2  Resin: Filtek Z250 | G_1_: 22.15±0.91; G_2_: 17.15±1.92  G_1_/G_2_^*^ |  |
| Nima et al, 2020[18] | 37% phosphoric acid +  G_1_: adhesive 1 + resin (24h) (7)  G_2_: 10% NaOCl + adhesive 1 + resin (24h) (7)  G_3_: adhesive 1 + resin (3h) (7)  G_4_: 10% NaOCl + adhesive 1 + resin (3h) (7)  G_5_: adhesive 1 + resin (1 year) (7)  G_6_: 10% NaOCl + adhesive 1 + resin (1 year) (7)  G_7_: adhesive 2 + resin (24h) (7)  G_8_: 10% NaOCl + adhesive 2 + resin (24h) (7)  G_9_: adhesive 2 + resin (3h) (7)  G_10_: 10% NaOCl + adhesive 2 + resin (3h) (7)  G_11_: adhesive 2 + resin (1 year) (7)  G_12_: 10% NaOCl + adhesive 2 + resin (1 year) (7) | Third molars | Water with thymol crystals | Adhesive: 1 - Gluma 2 Bond (Heraeus Kulzer, Germany); 2 – One Step  Resin: Charisma | G_1_: 48.4±13.1; G_2_: 36.7±6.7;  G_3_: 36.4±8.5; G_4_: 26.3±2.9;  G_5_: 43.0±5.7; G_6_: 31.1±4.7;  G_7_: 54.2±6.2; G_8_: 49.9±9.8;  G_9_: 41.4±5.8; G_10_: 39.1±7.9;  G_11_: 49.1±7.7; G_12_: 45.2±9.5  G_1_/G_2_;G_3_;G_5_^*^; G_2_/G_4_;G_6_^*^; G_3_/G_4_;G_5_^*^; G_4_/G_6_^*^; G_5_/G_6_^*^; G_7_/G_9_;G_11_^*^; G_8_/G_10_;G_12_^*^; G_9_/G_11_^*^; G_10_/G_12_^*^ |  |
| Vivanco et al, 2020[17] | G_1_: 37% phosphoric acid + adhesive 1 (20)  G_2_: 37% phosphoric acid + 0.2% CHX + adhesive 1 (20)  G_3_: 37% phosphoric acid + adhesive 1 (TC) (20)  G_4_: 37% phosphoric acid + 0.2% CHX + adhesive 1 (TC) (20)  G_5_: adhesive 2 (20)  G_6_: 0.2% CHX + adhesive 2 (20)  G_7_: adhesive 2 (TC) (20)  G_8_: 0.2% CHX + adhesive 2 (TC) (20) | Third molars | 0.1% Thymol + distilled water (4ºC) | Adhesive: 1 - Scotchbond multi-purpose; 2 – Single Bond Universal  Resin: Filtek Z350 | G_1_: 15.2±0.8; G_2_: 36.1±6.4;  G_3_: 23.9±5.0; G_4_: 25.6±5.0;  G_5_: 23.7±0.7; G_6_: 38.1±7.1;  G_7_: 21.3±0.8; G_8_: 36.7±6.9;  G_1_/G_2_;G_3_;G_5_^*^; G_2_/G_4_^*^; G_5_/G_6_; G_8_/G_3_;G_4_;G_7_^*^ |  |

CHX: Chlorhexidine, DT: Diamond Tip, DTUS: Ultrasonic Diamond Tip, EGCG: Epigallocatechin Gallate, MSP: Medium Short Pulse, PBS: Phosphate Buffer Saline, QSP: Quantum Square Pulse, TC: Thermocycling, *Statistically Significant Difference (p<0.05)
