# Supplementary material for: Effect of Cavity Disinfectants on Dentin Bond Strength and Clinical Success of Composite Restorations—A Systematic Review of In Vitro, In Situ and Clinical Studies
Source: Int J Mol Sci. 2020 Dec 31;22(1):353. doi: 10.3390/ijms22010353 (PMC7794949; doi:10.3390/ijms22010353)
Supplement: Supplementary file 1 [file ijms-22-00353-s001.zip › Supplementary Material S2.docx]

Supplementary material S2 – Results from the clinlcal studies included in the systematic review

| **Author, year** | **Groups (n)** | **Sample characteristics** | **Follow-up** | **Materials** | **Results** |
| --- | --- | --- | --- | --- | --- |
| Saboia et  al, 2006[163] | 37% phosphoric acid +  G_1_: adhesive 1 (14) G_2_: 10% NaOCl + adhesive 1 (14)  G_3_: adhesive 2 (14)  G_4_: 10% NaOCl + adhesive 2 (14)  + resin | 11 patients (47-56 years)  41 non-carious cervical lesions  Field isolation: relative | 2 years | Adhesive: 1 - Prime & Bond 2.1; 2 - Single Bond  Resin: Filtek Z250 | 12 months Post-operative sensitivity: Absent  Retention: G_1_: 81.8%; G_2_: 90%; G_3_: 90%; G_4_: 90% Marginal discoloration: G_1_: 90.9%; G_2_: 100%; G_3_: 100%; G_4_: 90% Secondary caries: Absent  24 months Post-operative sensitivity: Absent  Retention: G_1_: 63.3%; G_2_: 90%; G_3_: 90%; G_4_: 70% Marginal discoloration: G_1_: 81.8%; G_2_: 90%; G_3_:90%; G_4_: 90% Secondary caries: Absent |
| Dutra-Correa et al, 2013[165] | G_1_: 36% phosphoric acid + adhesive 1 (30) G_2_: 36% phosphoric acid + 2% CHX + adhesive 1 (30) G_3_: adhesive 2 (30)  G_4_: 2% CHX + adhesive 2 (30)  + resin | 37 patients (27-79 years)  120 non-carious cervical lesions  Field isolation: relative | 18 months | Adhesive: 1 - XP Bond; 2 - Xeno V Resin: Esthet X | No marginal discoloration  6 months: G_1_: 100%; G_2_: 96%; G_3_: 96.3%; G_4_: 95.8%  18 months: G_1_: 100%; G_2_: 90%; G_3_: 86.9%; G_4_: 100%  Marginal adaptation  6 months: G_1_: 88.9%; G_2_: 92%; G_3_: 85.2%; G_4_: 85.7%  18 months: G_1_: 88%; G_2_: 90%; G_3_: 86.9%; G_4_: 91.7%  No pre-operative sensitivity  G_1_: 64.5%; G_2_: 64.3%; G_3_: 60%; G_4_: 64.5%  No post-operative sensitivity  Baseline: G_1_: 90.3%; G_2_: 92.9%; G_3_: 86.7%; G_4_: 87.1%  6 months: G_1_: 92.3%; G_2_: 88%; G_3_: 100%; G_4_: 96.4%  18 months: G_1_: 96%; G_2_: 95%; G_3_: 91.3%; G_4_: 95.8%  Retention  6 months: G_1_/G_3_/G_4_ :100%; G_2_: 96%  18 months: G_1_: 100%; G_2_: 98%; G_3_: 91.3%; G_4_: 95.8%  No wear  6 months: G_1_: 100%; G_2_: 96%; G_3_/G_4_: 100%  18 months: G_1_: 92%; G_2_: 95%; G_3_: 86.9%; G_4_: 91.7%  Secondary caries  6 months: G_1_/G_3_/G_4_: 100%; G_2_: 96%  18 months: G_1_: 100%; G_2_: 95%; G_3_: 91.3%; G_4_: 95.8% |
| Sartori et al, 2013[161] | 35% phosphoric acid +  G_1_: none (35)  G_2_: 2% CHX (35)    + adhesive + resin | 20 patients (33-64 years)  70 non-carious cervical lesions  Field isolation: relative | 3 years | Adhesive: Single Bond 2  Resin: Filtek Supreme XT | Retention  6 months: G_1_: 94.3%; G_2_: 100%;12 months: G_1_: 93.5%; G_2_: 100%  36 months: G_1_: 88%; G_2_: 76%  G_2_: baseline/36 months^*^  Marginal discoloration  6 months: G_1_: 93.9%; G_2_: 97.1%; 12 months: G_1_: 93.1%; G_2_: 93.5%  36 months: G_1_: 72.7%; G_2_: 68.4%  G_1_: baseline/36 months^*^; G_2_: baseline/36 months^*^; 12 / 36 months^*^  Marginal integrity  6 months: G_1_/G_2_: 100%; 12 months: G_1_: 100%; G_2_: 96.8%;  36 months: G_1_: 90.9%; G_2_: 8.9%  Post-operative sensitivity  Baseline: G_1_: 82.9%; G_2_: 74.3%; 6 months: G_1_: 94.3%; G_2_: 67.1%  12 months: G_1_: 96.6%; G_2_: 87.1%; 36 months: G_1_: 95.5%; G_2_: 89.5%  Secondary caries - 0%  Pulp vitality - 100%  Clinical success  6 months: G_1_: 94.3%; G_2_: 100%; 12 months: G_1_: 93.5%; G_2_: 100%  36 months: G_1_: 88%; G_2_: 76% G_2_: baseline/36 months^*^ |
| Torres et al, 2014[162] | 37% phosphoric acid +  G_1_: none (68)  G_2_: 10% NaOCl (60s) (68)  + adhesive + resin | 30 patients (21-60 years)  138 non-carious cervical lesions  Field isolation: absolute | 5 years | Adhesive: Prime & Bond NT  Resin: Filtek A110 (3M, USA) | Restoration completely retained  18 months: G_1_: 98%; G_2_: 97%; 3 years: G_1_: 89%; G_2_: 87%  5 years: G_1_: 77%; G_2_: 68%  Pulp vitality - 100%  No marginal discoloration  18 months: G_1_: 98%; G_2_: 95%; 3 years: G_1_: 96%; G_2_: 91%  5 years: G_1_: 75%; G_2_: 75%  Marginal integrity  18 months: G_1_: 100%; G_2_: 98%; 3 years: G_1_: 98%; G_2_: 96%  5 years: G_1_: 78%; G_2_: 72%  No post-operative sensitivity  18 months: G_1_: 88%; G_2_: 86%; 3 years: G_1_: 86%; G_2_: 87%  5 years: G_1_: 88%; G_2_: 89%  No secondary caries  18 months: G_1_: 100%; G_2_: 98%; 3 years: G_1_: 100%; G_2_: 98%  5 years: G_1_: 100%; G_2_: 94% |
| Montagner et al, 2015[160] | 35% phosphoric acid +  G_1_: placebo solution (81)  G_2_: 2% CHX (88)  + adhesive + resin | 42 patients (>20 years)  169 non-carious cervical lesions  Field isolation: relative | 6 months | Adhesive: Single Bond 2  Resin: Filtek Z350 | Success: G_1_: 98.7%; G_2_: 96.4%  No marginal discoloration: G_1_: 97.5%; G_2_: 98.9% Fracture: G_1_/G_2_: 0%  Retention: G_1_/G_2_: 100%  Marginal adaptation: G_1_: 98.8%; G_2_: 100%  Post-operative sensitivity: G_1_/G_2_: 0%  Dental integrity: G_1_/G_2_: 100%  Pulp vitality: G_1_/G_2_: 100%  Periodontal health: G_1_/G_2_: 100% |
| Favetti et al, 2017[159] | 35% phosphoric acid +  G_1_: placebo solution (56)  G_2_: 2% CHX (49)  + adhesive + resin | 42 patients  105 non-carious cervical lesions  Field isolation: relative | 3 years | Adhesive: Single Bond 2  Resin: Filtek Z350 | No significant differences between groups regarding survival rate, marginal adaptation, marginal discoloration, post-operative sensitivity, fracture, pulp vitality or dental integrity. |
| Akarsu et al, 2020[164] | G_1_: adhesive (20)  G_2_: 37% phosphoric acid + adhesive (20)  G_3_: laser + adhesive (20)  G_4_: laser + phosphoric acid + adhesive (20) | 20 patients (38-62 years)  80 non-carious cervical lesions  Field isolation: relative | 18 months | Adhesive: Single bond  Resin: GrandioSO | 6 months  Retention: G_1_: 70%; G_2_/G_3_: 85%; G_4_: 90%  No marginal discoloration: G_1_: 60%; G_2_/G_3_: 75%; G_4_: 85%  Marginal integrity (closely adapted): G_1_: 70%; G_2_/G_3_: 85%; G_4_: 90%  No secondary caries: G_1_: 70%; G_2_/G_3_: 85%; G_4_: 90%  No hypersensitivity: G_1_: 50%; G_2_: 35%; G_3_: 55%; G_4_: 65%  18 months  Retention: G_1_: 60%; G_2_/G_3_/G_4_: 80%  No marginal discoloration: G_1_: 45%; G_2_: 70%; G_4_: 65%; G_4_: 75%  Marginal integrity (closely adapted): G_1_: 50%; G_2_/G_3_: 70%; G_4_: 75%  No secondary caries: G_1_: 60%; G_2_/G_3_/G_4_: 80%  No hypersensitivity: G_1_: 40%; G_2_: 25%; G_3_/G_4_: 50% |

CHX: Chlorhexidine, *Statistically Significant Difference (p<0.05)
